# Global and regional burden of liver cancer attributable to drug use in elderly patients: a 1990–2021 analysis from the GBD study

**DOI:** 10.3389/fonc.2026.1678700

**Published:** 2026-02-24

**Authors:** Chengchi Xia, Tianshu Rong, Baoqing Wang

**Affiliations:** Department of Oncology, The Second Affiliated Hospital of Xuzhou Medical University, Xuzhou,Jiangsu, China

**Keywords:** attributable burden, disability-adjusted life years, drug use, elderly population, global burden of disease, liver cancer, mortality

## Abstract

**Background:**

The global burden of liver cancer is undergoing an etiological shift, driven by population aging and the increasing complexity of pharmacological management. While Drug-Induced Liver Injury (DILI) is a recognized carcinogenic mechanism, the population-level impact remains under-quantified. This study aims to quantify the spatiotemporal trends of liver cancer burden attributable to drug use in the elderly population (aged 55+) and elucidate the drivers behind regional disparities.

**Methods:**

Leveraging data from the Global Burden of Disease (GBD) Study 2021, we analyzed incidence, mortality, and disability-adjusted life years (DALYs) across 204 countries and territories from 1990 to 2021. Adopting the Comparative Risk Assessment (CRA) framework, we estimated the specific burden by calculating the Population Attributable Fraction (PAF) relative to a theoretical minimum risk exposure level (TMREL) of zero drug use. Temporal trends were assessed using Estimated Annual Percentage Change (EAPC), and associations with the Socio-demographic Index (SDI) were evaluated to delineate developmental disparities.

**Results:**

Globally, the absolute number of deaths has steadily increased despite stable age-standardized rates. A distinct “SDI Divergence” was observed: High-SDI regions exhibited the most rapid escalation in burden (highest EAPC), driven by the “Opioid-Polypharmacy Nexus,” whereas Low-SDI regions sustained a persistently high baseline burden due to unmet diagnostic needs. Demographic analysis revealed a stark male predominance and identified the 55–74 age group as the “active intervention window,” accounting for the largest proportion of the global burden in terms of both mortality and DALYs.

**Discussion:**

The escalating burden of liver cancer attributable to drug use in the elderly underscores the “Cumulative Impact of Prolonged Exposure,” where the intersection of physiological aging and complex drug use patterns amplifies hepatic risk. Mitigating this crisis requires stratified strategies: prioritizing “Capacity Building” (integrating screening into infectious disease programs) in resource-limited settings, and implementing strict “Stewardship” (pharmacovigilance and active deprescribing) in developed nations to curb this trajectory.

## 1 Introduction

Primary liver cancer, particularly hepatocellular carcinoma (HCC), remains the third leading cause of cancer-related mortality worldwide (1), yet its epidemiological landscape is undergoing a profound transformation. While viral hepatitis has historically dominated the etiological spectrum, global population aging and the rising prevalence of metabolic syndromes are diversifying the risk profile (2–4). In this shifting context, Drug-Induced Liver Injury (DILI) has emerged as a critical, yet often underappreciated, independent driver of carcinogenesis (5). Unlike acute toxicity, chronic DILI creates a pro-inflammatory microenvironment conducive to malignant transformation (6). Research indicates that recurrent oxidative stress and immune-mediated injury from hepatotoxic agents can facilitate mutagenesis, establishing DILI not merely as a functional impairment, but as a direct oncogenic trigger (7). This risk is disproportionately magnified in the elderly population due to the intersection of physiological senescence and therapeutic complexity. As hepatic blood flow and metabolic reserve decline with age, the liver becomes increasingly vulnerable to xenobiotic insults (8, 9). Concurrently, the management of age-related multimorbidity necessitates “polypharmacy,” exposing elderly patients to cumulative risks from complex regimens involving antibiotics, chemotherapeutics, and analgesics (10–12). This creates a “double-hit” phenomenon: a physiologically fragile liver subjected to intensifying chemical exposure, thereby lowering the threshold for drug-induced carcinogenesis. Despite this growing clinical reality, global epidemiological evidence remains fragmented. To bridge this gap, this study leverages the Global Burden of Disease (GBD) 2021database to quantify the burden of drug-induced liver cancer specifically in the elderly. Crucially, for the purpose of this analysis, “drug use” is defined within the GBD framework to encompass a dual spectrum of hepatotoxicity: it captures both the sequelae of illicit injection drug use (IDU)—a primary historical vector for viral transmission—and the increasingly prevalent iatrogenic liver injury stemming from the abuse or prolonged use of prescribed medications. By stratifying these risks across age, gender, and sociodemographic levels, this study aims to inform targeted geriatric pharmacovigilance strategies.

## 2 Methods

### 2.1 Data collection

The data sources for the Global Burden of Disease (GBD) study include census data, household surveys, disease registries, health service utilization data, and vital statistics records. The GBD 2021 disease and injury burden analysis utilized 100,893 data sources to assess the disability life years (YLD), years of life lost (YLL), disability-adjusted life years (DALYs), and mortality for 371 diseases and injuries across 204 countries and 811 subnational locations (13–15). In this study, the liver cancer diagnosis is based on ICD-10 codes ‘C22–C22.8, D13.4.’ Disease burden is assessed using two indicators: mortality rates and DALYs. Age-standardized rates were applied to adjust for population age distribution differences, facilitating comparisons.

Relevant data were obtained from the GHDx (http://ghdx.healthdata.org/) platform, covering 204 countries, 27 geographical regions, 5 Socio-demographic Index(SDI) quintiles, and global data from 1990 to 2021 on incidence, mortality, and DALYs (13–15). The SDI is a composite indicator of development status, calculated as the geometric mean of lag-distributed income per capita, average educational attainment, and total fertility rate. With scores ranging from 0 to 1, higher values indicate higher socioeconomic development. In this study, locations were stratified into five SDI quintiles (Low, Low-middle, Middle, High-middle, and High) to explore the association between development levels and the burden of liver cancer attributable to drug use (16). Additionally, the search parameters were set as follows: Disease cause is ‘Liver cancer,’ risk factor is ‘Drug use,’ and the indicators include ‘deaths, YLLs (years of life lost due to premature mortality), YLDs (years lived with disability), DALYs (disability-adjusted life years).’ Crucially, in accordance with the GBD 2021 framework, the risk factor ‘Drug use’ is explicitly defined as drug use disorders (encompassing dependence on opioids, cocaine, amphetamines, and cannabis) (17, 18). The modeling of liver cancer attributable to this risk factor is primarily based on the pathway of injection drug use (IDU), which acts as a vector for the transmission of Hepatitis B and C viruses—key oncogenic factors for hepatocellular carcinoma.

The regions selected are ‘all regions,’ the time range is ‘1990-2021,’ the units are ‘value and rates,’ and gender includes ‘male, female, and total population.’ Age stratification includes ‘age-standardized, 55 years and above, and corresponding 5-year age groups.’ The age threshold of 55 was selected to capture the early onset of burden driven by drug-induced “accelerated aging.” This cutoff rests on two rationales: (1) The Pathophysiological Inflection Point: 55 years marks the critical juncture where decades of cumulative hepatotoxicity transition into clinical malignancy, reflecting the long latency of carcinogenesis. (2) Premature Hepatic Senescence: Chronic drug exposure precipitates functional decline that outpaces chronological aging. Lowering the threshold to 55—prior to the traditional geriatric cutoff of 65—prevents the underestimation of the significant “hidden burden” emerging in late middle age. This study follows the reporting guidelines for cross-sectional studies as outlined in the ‘Accuracy and Transparency Guidelines for Health Estimation Reports (GATHER).

### 2.2 Statistical analysis

This study utilized data from the Global Burden of Disease (GBD) database to describe the global, regional, and national mortality rates and DALY rates of liver cancer attributable to drug use in elderly patients from 1990 to 2021. The Age-Standardized Mortality Rate (ASMR) and Age-Standardized Disability Rate (ASDR) were calculated for global, regional, and national levels. To quantify the burden specifically attributable to drug use, we applied the Population Attributable Fraction (PAF) methodology within the GBD Comparative Risk Assessment (CRA) framework. The PAF represents the proportion of risk that would be reduced if the exposure to the risk factor were reduced to the Theoretical Minimum Risk Exposure Level (TMREL), defined as zero exposure. The PAF was estimated using the standard integral formula (19, 20):


$$PAF=\frac{\int_{x=0}^{m}RR\left(x\right)P\left(x\right)dx-\int_{x=0}^{m}RR\left(x\right)P^{\prime }\left(x\right)dx}{\int_{x=0}^{m}RR\left(x\right)P\left(x\right)dx}$$

Where RR(x) is the relative risk at exposure level x, P(x) is the population distribution ofexposure, P’(x) is the distribution at the TMREL, and m is the maximum exposure level.World maps of ASMR and ASDR were generated to visualize the geographic distribution. The Estimated Annual Percentage Change (EAPC) was calculated to assess temporal trendsusing the formula 100 * (exp(β) - 1), with the 95% confidence interval (CI) derived fromthe linear regression model (y = α + βx + ε, where y is ln(ASMR or ASDR) and x is the calendar year).

The composition of age-related burdens due to drug-induced liver cancer in elderly patients in 1990 and 2021 was compared based on SDI quintiles. Gender differences in the age-related burden among countries were analyzed. Elderly patients were categorized into age groups: 55-59, 60-64, 65-69, 70-74, 75-79, 80-84, 85-89, 90-94, and 95+ years. All statistical analyses were conducted using R software (version 4.4.2). A p-value lower than 0.05 was deemed statistically significant.

## 3 Results

### 3.1 Global burden of drug-induced liver cancer in elderly patients from 1990 to 2021

**Figure 1:**
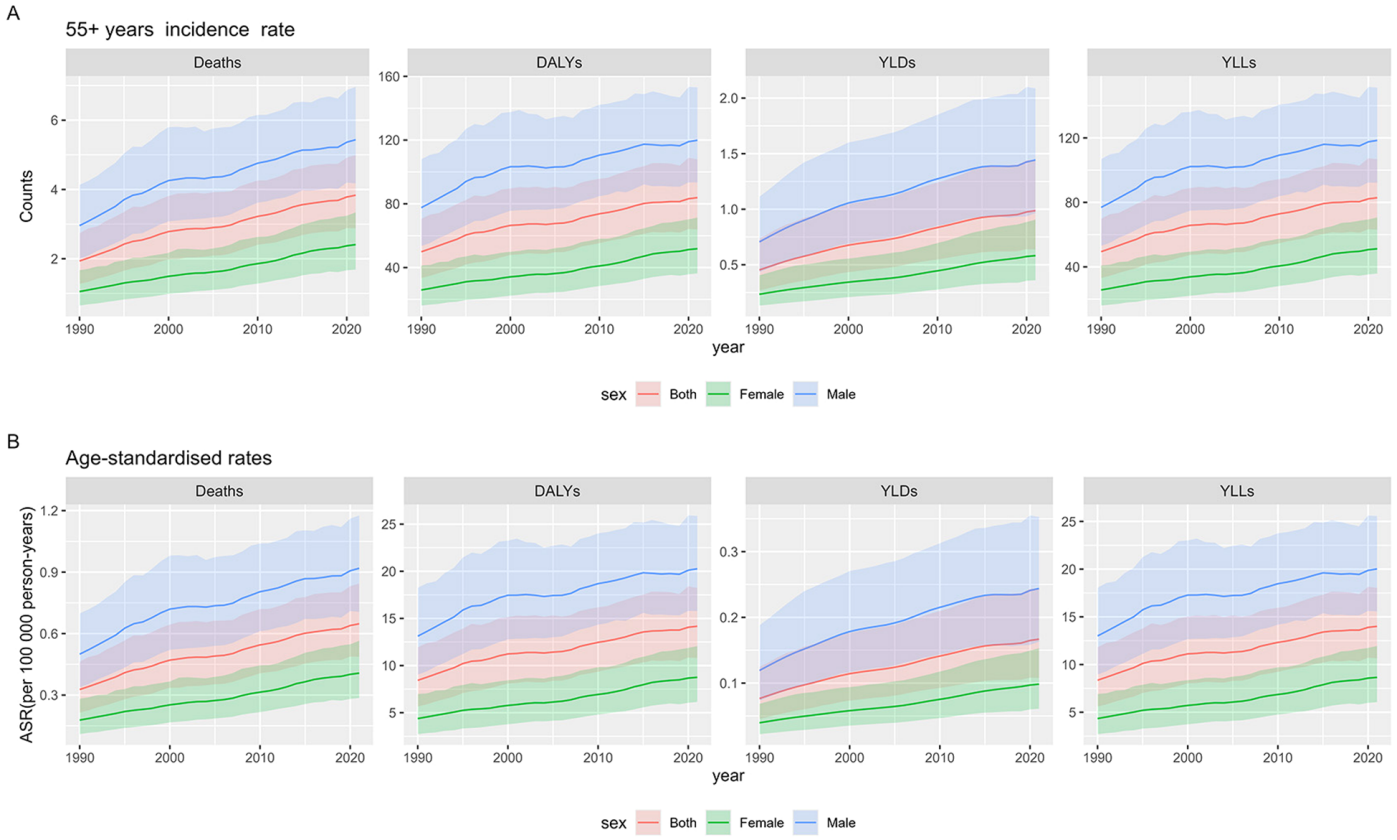
Global burden of drug-induced liver cancer in elderly patients. **(A)** 55+ years counts; **(B)** ASR, age-standardized rates.

From 1990 to 2021, the number of deaths from liver cancer attributable to drug use among elderly patients aged 55 and above showed a significant upward trend globally. Specifically, the number of deaths increased from 12,994 (95% UI: 8,542.6–18,372.0) in 1990 to 56,966 (95% CI: 42,813.5–74,178.9) in 2021. In terms of gender differences, the number of male deaths rose from 9,206 (95% UI: 6,222.1-12,875.0) in 1990 to 38,029 (95% CI: 29,202.3-48,691.7) in 2021. Throughout the study period, male deaths were consistently higher than female deaths. Specifically, in 2021, male deaths were approximately twice as high as female deaths (18,937; 95% CI: 13,287.7-26,285.8) (**Figure 1A**).

Simultaneously, DALYs, YLDs, and YLLs due to liver cancer attributable to drug use in elderly patients aged 55 and above also showed an upward trend. DALYs increased from 334,945 (95% CI: 224,326.0–474,000.0) to 1,246,150 (95% CI: 949,800.0–1,603,029.0), with males accounting for 838,767 (95% CI: 653,345.0–1,070,670.0) cases, 2.06 times higher than females, who accounted for 407,383 (95% CI: 285,949.0–561,331.0) cases. YLDs increased from 3,042 (95% CI: 1,806.9–4,943.9) to 14,674 (95% CI: 9,486.2–21,393.3), with males contributing 10,095 (95% CI: 6,746.0–14,600.1) cases, females contributed 4,579 (95% CI: 2,839.5–7,124.1) cases. YLLs increased from 331,903 (95% CI: 222,169.0–469,224.0) to 1,231,476 (95% CI: 937,063.0–1,586,394.0), with males accounting for 828,672 (95% CI: 644,652.28–1,057,748.10) cases, 2.06 times higher than females, who accounted for 402,804 (95% CI: 282,255.0–555,823.0) cases.

Furthermore, from 1990 to 2021, the Age-Standardized Mortality Rate (ASMR) due to liver cancer attributable to drug use in elderly patients aged 55 and above also showed an upward trend, increasing from 0.33 (95% CI: 0.22–0.46) per 100,000 in 1990 to 0.66 (95% CI: 0.49–0.84) per 100,000 in 2021. Regarding the specific rates by gender, the global ASMR for males in 2021 was 0.9 (95% CI: 0.7–1.1) per 100,000, significantly higher than the female ASMR of 0.4 (95% CI: 0.3–0.6) per 100,000. This corresponds to an approximate 2.25-fold difference, which aligns with the gender disparity observed in absolute death counts. Similarly, the Age-Standardized DALY Rate (ASDR) increased from 8.43 (95% CI: 5.64–11.93) per 100,000 in 1990 to 14.17 (95% CI: 10.70–18.23) per 100,000 in 2021 (**Figure 1B**).

### 3.2 Region burden of drug-induced liver cancer in elderly patients from 1990 to 2021

Based on SDI quintile analysis, from 1990 to 2021, the mortality rates and DALY rates due to drug-induced liver cancer in elderly patients showed an upward trend across global regions. Specifically, High SDI countries experienced the most significant increases in age-standardized mortality rates and DALY rates. The EAPC of deaths in High SDI countries was 3.15 (95% CI: 2.86–3.44), and the EAPC of ASDR was 2.54 (95% CI: 2.27–2.82). The age-standardized mortality rate increased from 0.4 (95% UI: 0.3–0.5) per 100,000 in 1990 to 1.1 (95% CI: 0.8–1.3) per 100,000 in 2021, while the age-standardized DALY rate increased from 9.5 (95% CI: 6.5–13.8) per 100,000 in 1990 to 22.2 (95% CI: 17.6–27.5) per 100,000 in 2021. In contrast, High-middle SDI countries experienced the smallest increases in age-standardized mortality rates and DALY rates. The EAPC of deaths was 1.34 (95% CI: 1.18–1.51), and the age-standardized mortality rate rose from 0.4 (95% CI: 0.3–0.6) per 100,000 in 1990 to 0.7 (95% CI: 0.5–1) per 100,000 in 2021. The EAPC of ASDR was 0.8 (95% CI: 0.66–0.94), and the age-standardized DALY rate increased from 11.1 (95% CI: 7.2–15.7) per 100,000 in 1990 to 15.8 (95% CI: 11.3–21) per 100,000 in 2021. By 2021, regions with High SDI countries had the highest mortality and DALY rates, whereas Middle and Low-middle SDI regions remained relatively lower.

At the regional level, from 1990 to 2021, the global burden of liver cancer attributable to drug use in elderly patients showed an overall upward trend. Southern Latin America experienced the largest increase, with its mortality rate rising from 0 (95% CI: 0–0.1) per 100,000 in 1990 to 0.2 (95% CI: 0.1–0.4) per 100,000 in 2021, with an EAPC of deaths of 7.14 (95% CI: 6.91–7.36). Australasia also experienced a significant rise, with its rate increasing from 0.1 (95% CI: 0–0.3) per 100,000 to 1 (95% CI: 0.2–1.5) per 100,000, and an EAPC of 6.06 (95% CI: 5.82–6.3). High-income North America also saw an increase, from 0.4 (95% CI: 0.3–0.5) per 100,000 in 1990 to 1.3 (95% CI: 1.1–1.5) per 100,000 in 2021, with an EAPC of deaths of 3.95 (95% CI: 3.74–4.17). On the other hand, Oceania had the smallest increase in mortality, with its rate rising slightly from 0.4 (95% CI: 0.1–1) per 100,000 in 1990 to 0.5 (95% CI: 0.2–0.9) per 100,000 in 2021, with an EAPC of deaths of 0.35 (95% CI: 0.18–0.53).

Similarly, from 1990 to 2021, the global burden of age-standardized DALYs due to liver cancer attributable to drug use in elderly patients also showed an upward trend. Southern Latin America experienced the largest increase, with its age-standardized DALY rate rising from 0.9 (95% CI: 0.3–1.9) per 100,000 in 1990 to 5.7 (95% CI: 2.1–10.4) per 100,000 in 2021, with an EAPC of 6.76 (95% CI: 6.53–7.0). Australasia also saw a significant rise, reaching an EAPC of 5.60 (95% CI: 5.39–5.8). High-income North America showed a notable increase, from 9 (95% CI: 7.2–11) per 100,000 to 28.4 (95% CI: 23.8–33.3) per 100,000, with an EAPC of 3.87 (95% CI: 3.66–4.08). In contrast, High-income Asia Pacific remained relatively stable, with an EAPC of 0.1 (95% CI: -0.34–0.53). Oceania also showed a minimal increase, with its rate rising from 10.3 to 11.5 per 100,000, and an EAPC of 0.17 (95% CI: 0.03–0.3). Western Sub-Saharan Africa’s age-standardized DALY rate increased from 1.2 (95% CI: 0.5–2.4) per 100,000 in 1990 to 2 (95% CI: 1–3.4) per 100,000 in 2021, with an EAPC of DALYs of 1.35 (95% CI: 1.25–1.46).

The study characterized the relationship between age-standardized DALY rates and the Sociodemographic Index (SDI) from 1990 to 2021. Contrary to a U-shaped pattern, the data revealed a positive association, where regions with High SDI levels experienced the highest disease burden (ASDR: 22.2 per 100,000), while regions with Low SDI levels had the lowest burden (ASDR: 4.5 per 100,000). Regional analysis highlighted that Central Asia (35.7), High-income Asia Pacific (25.1), and Australasia (20.4) exhibited particularly high DALY rates, potentially due to factors such as aging populations and higher prevalence of drug use. In contrast, regions such as Western Sub-Saharan Africa (2.0) and Southern Sub-Saharan Africa (2.5) had the lowest burdens. Notably, this burden distribution was underpinned by a consistent gender disparity: across all regions, male patients exhibited significantly higher mortality and DALY rates attributable to drug use than females (**Table 1**). These findings suggest that high-SDI regions currently face the greatest challenge from liver cancer attributable to drug use, requiring targeted strategies for risk factor management in elderly populations (**Supplementary Figure S1**).

**Table 1:** Region and sex burden of drug-induced liver cancer in elderly patients.

Location	Sex	Mortality(95%UI)		Mortality(95%UI)			DALY (95%UI)		DALY (95%UI)		
		Case,1990	ASMR(per100000),	Case,2021	ASMR(per100000),	EAPC of ASMR(95%CI),	Case,1990	ASDR(per100000),	Case,2021	ASDR(per100000),	EAPC of ASDR(95%CI),
			1990		2021	1990-2021		1990		2021	1990-2021
Global	Both	12993.9 (8542.6-18372)	0.3 (0.2-0.5)	56966.3 (42813.5-74178.9)	0.6 (0.5-0.8)	2.01 (1.87-2.16)	334945.3 (224326-474000.1)	8.4 (5.6-11.9)	1246150 (949799.9-1603029.3)	14.2 (10.8-18.2)	1.5 (1.38-1.62)
	Male	9205.8 (6222.1-12875)	0.5 (0.3-0.7)	38029 (29202.3-48691.7)	0.9 (0.7-1.1)	1.72 (1.55-1.9)	241694.1 (166647.8-336401.2)	13.1 (9-18.3)	838767.2 (653345-1070669.6)	19.7 (15.3-24.8)	1.18 (1.04-1.33)
	Female	3788.1 (2328.2-6008.9)	0.2 (0.1-0.3)	18937.4 (13287.7-26285.8)	0.4 (0.3-0.6)	2.61 (2.52-2.69)	3788.1 (2328.2-6008.9)	0.2 (0.1-0.3)	18937.4 (13287.7-26285.8)	0.4 (0.3-0.6)	2.61 (2.52-2.69)
High SDI	Both	4106.3 (2860.5-5938.1)	0.4 (0.3-0.5)	21737.9 (17108.8-26805.7)	1.1 (0.8-1.3)	3.15 (2.86-3.44)	104798.7 (72058.4-152224.4)	9.5 (6.5-13.8)	452900.9 (359746.9-561134.6)	22.2 (17.6-27.5)	2.54 (2.27-2.82)
	Male	3158.7 (2167.3-4633.1)	0.7 (0.5-1)	15363.1 (12054.8-19011.8)	1.5 (1.2-1.9)	2.56 (2.24-2.88)	82524.3 (55923.3-122098.4)	17.2 (11.7-25.5)	324585.4 (259237-403084.4)	32.8 (26.1-40.7)	1.93 (1.62-2.24)
	Female	947.6 (691.4-1296.4)	0.2 (0.1-0.2)	6374.8 (4971.1-7924.3)	0.6 (0.5-0.7)	4.34 (4.14-4.55)	947.6 (691.4-1296.4)	0.2 (0.1-0.2)	6374.8 (4971.1-7924.3)	0.6 (0.5-0.7)	4.34 (4.14-4.55)
High-middle SDI	Both	4395.1 (2828-6273.6)	0.4 (0.3-0.6)	14796.2 (10442.9-19969.8)	0.7 (0.5-1)	1.34 (1.18-1.51)	113348.1 (73544.6-159889.6)	11.1 (7.2-15.7)	323105.2 (231055-429755.1)	15.8 (11.3-21)	0.8 (0.66-0.94)
	Male	3072.4 (2068.8-4275.4)	0.7 (0.5-1)	9580.6 (6898.9-12617.5)	1 (0.7-1.3)	0.89 (0.71-1.07)	80618.1 (54730.9-111139.7)	18.1 (12.3-25)	211063.3 (154809.4-276523.8)	22 (16.2-28.6)	0.33 (0.18-0.49)
	Female	1322.7 (765.8-2095.9)	0.2 (0.1-0.4)	5215.6 (3257.6-7793.6)	0.5 (0.3-0.7)	2.1 (1.96-2.24)	1322.7 (765.8-2095.9)	0.2 (0.1-0.4)	5215.6 (3257.6-7793.6)	0.5 (0.3-0.7)	2.1 (1.96-2.24)
Middle SDI	Both	3471.1 (2075.8-5347.7)	0.3 (0.2-0.5)	15457.7 (10163.5-21611.3)	0.6 (0.4-0.8)	1.56 (1.42-1.7)	89370 (54304.1-137425.6)	8.7 (5.3-13.4)	348561.2 (232716.2-486253.2)	12.5 (8.4-17.5)	1.13 (0.98-1.28)
	Male	2245.3 (1372.7-3472.7)	0.4 (0.3-0.7)	9804.6 (6586.4-13668.4)	0.7 (0.5-1)	1.56 (1.44-1.67)	58745.5 (36620.8-88545.8)	11.7 (7.3-17.7)	222671.8 (148165.1-314045.1)	16.3 (11.1-22.8)	1.11 (0.99-1.23)
	Female	1225.8 (653.7-2169.4)	0.2 (0.1-0.4)	5653.1 (3311.9-8658.4)	0.4 (0.2-0.6)	1.62 (1.4-1.84)	1225.8 (653.7-2169.4)	0.2 (0.1-0.4)	5653.1 (3311.9-8658.4)	0.4 (0.2-0.6)	1.62 (1.4-1.84)
Low-middle SDI	Both	821.3 (465.6-1285.7)	0.1 (0.1-0.2)	4046.1 (2633.2-5747.6)	0.3 (0.2-0.4)	2.3 (2.26-2.35)	22117.3 (12632.9-34649.3)	3.7 (2.1-5.8)	98969.4 (65921.9-141469.7)	6.9 (4.6-9.9)	1.97 (1.91-2.03)
	Male	591.9 (344.4-928.2)	0.2 (0.1-0.3)	2704.4 (1851.1-3774)	0.4 (0.3-0.5)	2.19 (2.13-2.25)	16112.9 (9367.6-25269.4)	5.3 (3.1-8.3)	66383.2 (45654-93533.5)	9.5 (6.5-13.3)	1.84 (1.76-1.91)
	Female	229.4 (118.9-392.5)	0.1 (0-0.1)	1341.7 (772.7-2103.7	0.2 (0.1-0.3)	2.81 (2.75-2.88)	229.4 (118.9-392.5)	0.1 (0-0.1)	1341.7 (772.7-2103.7)	0.2 (0.1-0.3)	2.81 (2.75-2.88)
Low SDI	Both	190.7 (103.6-320.7)	0.1 (0-0.1)	896 (552-1411.6)	0.2 (0.1-0.3)	2.33 (2.2-2.46)	5070.5 (2823.9-8312)	2.3 (1.3-3.8)	21909.4 (13470.7-34349.4)	4.5 (2.8-7.1)	2.05 (1.93-2.17)
	Male	131.3 (74.5-218.6)	0.1 (0.1-0.2)	555 (340-871.3)	0.2 (0.1-0.4)	2.1 (1.95-2.26)	3535.3 (2040.9-5778.9)	3.1 (1.8-5.1)	13594.2 (8306.9-21626.6)	5.7 (3.5-9)	1.79 (1.64-1.94)
	Female	59.4 (30-110.9)	0.1 (0-0.1)	341 (195.3-569.3)	0.1 (0.1-0.2)	2.92 (2.83-3.02)	59.4 (30-110.9)	0.1 (0-0.1)	341 (195.3-569.3)	0.1 (0.1-0.2)	2.92 (2.83-3.02)
Andean Latin America	Both	9.2 (3.5-17.7)	0 (0-0.1)	73.9 (28.2-136.3)	0.1 (0-0.2)	3.21 (2.97-3.45)	234.6 (88-455.3)	1.2 (0.4-2.3)	1641.2 (620.6-2965.4)	2.8 (1.1-5.1)	2.69 (2.44-2.93)
	Male	4.5 (1.7-8.7)	0 (0-0.1)	35.8 (14.6-64.7)	0.1 (0-0.2)	3.33 (3.07-3.58)	116 (43.2-224)	1.2 (0.4-2.3)	786.1 (319.3-1385.7)	2.7 (1.1-4.6)	2.76 (2.5-3.02)
	Female	4.6 (1.6-9.5)	0 (0-0.1)	38.2 (13.1-74.3)	0.1 (0-0.2)	3.1 (2.87-3.33)	4.6 (1.6-9.5)	0 (0-0.1)	38.2 (13.1-74.3)	0.1 (0-0.2)	3.1 (2.87-3.33)
Australasia	Both	33.2 (8.8-61.3)	0.1 (0-0.3)	497.8 (78.7-780.5)	1 (0.2-1.5)	6.06 (5.82-6.3)	831.7 (221.8-1532.3)	3.6 (1-6.6)	10672.4 (1788-16526.5)	20.4 (3.4-31.6)	5.6 (5.39-5.8)
	Male	25.9 (7.1-47.9)	0.2 (0.1-0.4)	336 (57.7-531.3)	1.2 (0.2-2)	5.41 (5.17-5.64)	647.1 (180.4-1171.6)	6.1 (1.7-11)	7145.6 (1288.7-10922.7)	27 (4.8-42.2)	4.91 (4.7-5.12)
	Female	7.4 (1.6-14.5)	0.1 (0-0.1)	161.8 (23.8-275.1)	0.6 (0.1-1)	7.54 (7.21-7.87)	7.4 (1.6-14.5)	0.1 (0-0.1)	161.8 (23.8-275.1)	0.6 (0.1-1)	7.54 (7.21-7.87)
Caribbean	Both	16.2 (7.2-29.7)	0.1 (0-0.1)	83.9 (41.3-137.2)	0.2 (0.1-0.3)	2.46 (2.2-2.73)	403.6 (178.6-756.3)	1.6 (0.7-3)	1940 (949.3-3273.2)	3.5 (1.7-6)	2.23 (1.97-2.49)
	Male	10 (4.4-18.8)	0.1 (0-0.2)	55.3 (27.1-92.6)	0.2 (0.1-0.3)	2.74 (2.46-3.02)	248.8 (110.1-467.2)	2 (0.9-3.8)	1287 (627.2-2147)	4.8 (2.2-8)	2.54 (2.27-2.81)
	Female	6.2 (2.8-11.5)	0 (0-0.1)	28.6 (13.8-49.4)	0.1 (0-0.2)	2.03 (1.76-2.31)	6.2 (2.8-11.5)	0 (0-0.1)	28.6 (13.8-49.4)	0.1 (0-0.2)	2.03 (1.76-2.31)
Central Asia	Both	490.8 (250-803.8)	1 (0.5-1.7)	1231.6 (738.9-1851.2)	1.4 (0.9-2.2)	0.59 (0.38-0.8)	13002 (6588.1-21335.8)	27.5 (13.9-45.1)	30731.6 (18191.7-46522.9)	35.7 (21.1-54)	0.32 (0.17-0.46)
	Male	308.5 (152.2-509.1)	1.6 (0.8-2.7)	698.6 (426.4-1020.5)	1.8 (1.1-2.6)	-0.06 (-0.27-0.14)	8319 (4192.1-13742.6)	43.4 (21.9-71.7)	17771.7 (10561.7-26485.9)	45.2 (27.4-67.9)	-0.32 (-0.48--0.17)
	Female	182.2 (88.3-313.6)	0.6 (0.3-1.1)	533 (305.2-858)	1.1 (0.6-1.8)	1.41 (1.16-1.65)	182.2 (88.3-313.6)	0.6 (0.3-1.1)	533 (305.2-858)	1.1 (0.6-1.8)	1.41 (1.16-1.65)
Central Europe	Both	319 (169.9-518.7)	0.2 (0.1-0.3)	940.1 (600.1-1311.5)	0.4 (0.3-0.6)	2.08 (1.9-2.26)	8158 (4349.3-13091.3)	5.2 (2.8-8.3)	20174.3 (13155.1-28275)	9.2 (6-12.9)	1.52 (1.36-1.68)
	Male	182.2 (99.5-295.4)	0.3 (0.1-0.4)	551.1 (363.4-777.6)	0.6 (0.4-0.8)	2.19 (2.02-2.36)	4701.4 (2541.5-7608.7)	6.9 (3.7-11.2)	12033 (7884.7-17046.6)	12.2 (8.1-17.3)	1.66 (1.52-1.81)
	Female	136.9 (68.2-220.9)	0.2 (0.1-0.2)	389.1 (242.7-565.8)	0.3 (0.2-0.5)	1.9 (1.69-2.1)	136.9 (68.2-220.9)	0.2 (0.1-0.2)	389.1 (242.7-565.8)	0.3 (0.2-0.5)	1.9 (1.69-2.1)
Central Latin America	Both	85.6 (45.3-145.5)	0.1 (0.1-0.2)	750.8 (443.1-1133.2)	0.3 (0.2-0.4)	3.35 (3.18-3.53)	2221 (1162.5-3769.1)	2.8 (1.4-4.7)	17254.7 (10040.2-26125.4)	6.8 (4-10.3)	2.94 (2.72-3.15)
	Male	61.9 (32.8-105.7)	0.2 (0.1-0.3)	564.3 (332.9-855.5)	0.4 (0.3-0.7)	3.6 (3.38-3.83)	1611.9 (847.5-2742.9)	4.2 (2.2-7.1)	12975.4 (7641.1-19780)	10.3 (6.3-15.2)	3.18 (2.91-3.45)
	Female	23.7 (12-40.7)	0.1 (0-0.1)	186.5 (106.8-290.2)	0.1 (0.1-0.2)	2.93 (2.81-3.05)	23.7 (12-40.7)	0.1 (0-0.1)	186.5 (106.8-290.2)	0.1 (0.1-0.2)	2.93 (2.81-3.05)
Central Sub-Saharan Africa	Both	9.4 (2.7-24.5)	0 (0-0.1)	37.3 (11.9-92.6)	0.1 (0-0.2)	1.12 (0.97-1.28)	254.1 (73.1-667.6)	1.1 (0.3-3)	978.4 (321.2-2454.5)	1.8 (0.6-4.6)	1.06 (0.89-1.23)
	Male	6.5 (1.9-17.2)	0.1 (0-0.2)	23.6 (7.4-65.7)	0.1 (0-0.3)	0.77 (0.57-0.98)	176 (48.8-466.4)	1.7 (0.5-4.5)	631.5 (203.4-1734)	2.4 (0.8-6.9)	0.74 (0.54-0.95)
	Female	3 (0.6-9.3)	0 (0-0.1)	13.6 (2.7-42.4	0 (0-0.1)	1.77 (1.68-1.87)	3 (0.6-9.3)	0 (0-0.1)	13.6 (2.7-42.4)	0 (0-0.1)	1.77 (1.68-1.87)
East Asia	Both	4668.5 (2712.1-7162.5)	0.5 (0.3-0.8)	17999 (11351.3-24975.5)	0.8 (0.5-1.1)	1.04 (0.87-1.21)	119504.9 (68637.4-178858.5)	13.6 (7.8-20.3)	396310.3 (255473.8-543939.7)	17.1 (11-23.4)	0.57 (0.42-0.73)
	Male	2999.1 (1724.4-4555)	0.7 (0.4-1.1)	10718.9 (7176.6-15355.9)	0.9 (0.6-1.3)	0.82 (0.65-0.99)	78274.4 (46164.4-117790.6)	18.1 (10.7-27.2)	237208.1 (157575.5-336501)	20.6 (13.8-29.2)	0.31 (0.16-0.46)
	Female	1669.4 (855.6-3033.2)	0.4 (0.2-0.7)	7280.1 (4073.5-11581.2)	0.6 (0.3-1)	1.45 (1.25-1.64)	1669.4 (855.6-3033.2)	0.4 (0.2-0.7)	7280.1 (4073.5-11581.2)	0.6 (0.3-1)	1.45 (1.25-1.64)
Eastern Europe	Both	625.6 (416.4-907.9)	0.2 (0.1-0.3)	1717.5 (1269.4-2158.6)	0.5 (0.3-0.6)	2.51 (2.3-2.72)	15843.2 (10686.7-22686.7)	5.5 (3.7-7.8)	38074.3 (28742.4-47789.8)	10.4 (7.8-13)	2.03 (1.82-2.24)
	Male	354.9 (248.6-490.3)	0.3 (0.2-0.5)	945.3 (727.2-1167.3)	0.6 (0.5-0.8)	1.99 (1.85-2.12)	9269.5 (6561.3-12627.7)	9 (6.4-12.3)	22096.5 (17133.6-27356.6)	14.7 (11.7-18.2)	1.61 (1.47-1.76)
	Female	270.7 (158.6-417.2)	0.1 (0.1-0.2)	772.2 (513-1052.2)	0.3 (0.2-0.5)	3.02 (2.7-3.35)	270.7 (158.6-417.2)	0.1 (0.1-0.2)	772.2 (513-1052.2)	0.3 (0.2-0.5)	3.02 (2.7-3.35)
Eastern Sub-Saharan Africa	Both	43.5 (17.8-86.3)	0.1 (0-0.1)	212.9 (86.8-414.4)	0.1 (0.1-0.3)	2.35 (2.09-2.61)	1115.7 (443.7-2194.5)	1.6 (0.6-3)	5223.9 (2148.8-10305.3)	3.3 (1.3-6.4)	2.2 (1.95-2.44)
	Male	24.6 (9-51.4)	0.1 (0-0.1)	120.7 (46.1-291.5)	0.2 (0.1-0.4)	2.42 (2.02-2.82)	646.9 (227.2-1383.7)	1.8 (0.6-3.8)	3053.4 (1177.6-7163.6)	4 (1.5-9.3)	2.31 (1.93-2.69)
	Female	18.9 (8-36.5)	0.1 (0-0.1)	92.2 (38.8-166.6)	0.1 (0-0.2)	2.35 (2.29-2.41)	18.9 (8-36.5)	0.1 (0-0.1)	92.2 (38.8-166.6)	0.1 (0-0.2)	2.35 (2.29-2.41)
High-income Asia Pacific	Both	1462.2 (844.7-2373.8)	0.7 (0.4-1.1)	5149.6 (3338.7-7392.6)	1.2 (0.8-1.8)	1.08 (0.63-1.54)	40851.5 (23683.6-67065.1)	19.7 (11.4-32.4)	104816.6 (68510.4-147256.1)	25.1 (16.4-35.3)	0.1 (-0.34-0.53)
	Male	1367.4 (794.3-2239.8)	1.5 (0.9-2.5)	4592.3 (2988.7-6531)	2.2 (1.5-3.2)	0.79 (0.34-1.24)	38454 (22366.8-63180.9)	42.2 (24.6-69.4)	94256 (62639.2-130849.1)	46.6 (31.1-65.1)	-0.18 (-0.61-0.25)
	Female	94.8 (51.2-164.4)	0.1 (0-0.1)	557.3 (323.5-861.8)	0.2 (0.1-0.4)	2.95 (2.43-3.48)	94.8 (51.2-164.4)	0.1 (0-0.1)	557.3 (323.5-861.8)	0.2 (0.1-0.4)	2.95 (2.43-3.48)
High-income North America	Both	1307.3 (1032.5-1640.9)	0.4 (0.3-0.5)	8747 (7289.7-10172.9)	1.3 (1.1-1.5)	3.95 (3.74-4.17)	30883.8 (24597.6-37588.2)	9 (7.2-11)	189187.1 (158423.6-221605.8)	28.4 (23.8-33.3)	3.87 (3.66-4.08)
	Male	790.2 (635.8-968.7)	0.5 (0.4-0.7)	5330 (4494.7-6255.4)	1.6 (1.4-1.9)	3.83 (3.63-4.04)	18837.6 (15067.5-22633.7)	12.7 (10.2-15.3)	119629.6 (99868.4-139868.5)	37.2 (31.2-43.7)	3.86 (3.62-4.1)
	Female	517.1 (392.1-658)	0.3 (0.2-0.3)	3417.1 (2793.6-3985.5	1 (0.8-1.1)	3.94 (3.7-4.18)	517.1 (392.1-658)	0.3 (0.2-0.3)	3417.1 (2793.6-3985.5)	1 (0.8-1.1)	3.94 (3.7-4.18)
North Africa and Middle East	Both	68.8 (38.9-113.4)	0 (0-0.1)	589.6 (359.4-912.3)	0.1 (0.1-0.2)	3.9 (3.71-4.08)	1789.7 (1006.4-2978.5)	1.1 (0.6-1.8)	13945.9 (8009.6-22689)	3.1 (1.8-5)	3.62 (3.45-3.79)
	Male	52.9 (30.9-87.5)	0.1 (0-0.1)	485.1 (294.9-772.9)	0.2 (0.1-0.3)	4.13 (3.96-4.31)	1393.8 (807.3-2314.5)	1.6 (0.9-2.7)	11545.1 (6633.2-18998.1)	4.9 (2.8-8)	3.85 (3.69-4)
	Female	15.9 (7.7-30.4)	0 (0-0)	104.5 (60.9-161)	0 (0-0.1)	3 (2.76-3.24)	15.9 (7.7-30.4)	0 (0-0)	104.5 (60.9-161)	0 (0-0.1)	3 (2.76-3.24)
Oceania	Both	11.1 (3.9-28.3)	0.4 (0.1-1)	34.3 (15.4-65.6)	0.5 (0.2-0.9)	0.35 (0.18-0.53)	292.3 (102.8-749.5)	10.3 (3.6-26.3)	841.7 (385.9-1618.8)	11.5 (5.3-22.2)	0.17 (0.03-0.3)
	Male	7.8 (2.9-19.7)	0.5 (0.2-1.3)	20.3 (9.2-43.8)	0.5 (0.2-1.1)	-0.19 (-0.38-0)	201.2 (74-522.2)	13.6 (5-35.3)	492.6 (219.8-1094.8)	12.7 (5.7-26.6)	-0.36 (-0.51--0.21)
	Female	3.4 (0.9-9)	0.2 (0.1-0.7)	14 (5.6-28.4)	0.4 (0.2-0.8)	1.3 (1.13-1.48)	3.4 (0.9-9)	0.2 (0.1-0.7)	14 (5.6-28.4)	0.4 (0.2-0.8)	1.3 (1.13-1.48)
South Asia	Both	753.3 (482.3-1128.4)	0.1 (0.1-0.2)	4429.9 (3131.8-6032)	0.3 (0.2-0.4)	2.5 (2.39-2.6)	20410.7 (13054.4-30057.1)	3.6 (2.3-5.4)	107325.5 (74642.2-145089.4)	7.3 (5.1-9.9)	2.11 (2-2.23)
	Male	582 (374.1-849.5)	0.2 (0.1-0.3)	3027.2 (2173.9-4026.7)	0.4 (0.3-0.5)	2.29 (2.18-2.4)	15844.8 (10266.9-22782.6)	5.4 (3.5-7.7)	72932.1 (52775.3-98291.7)	10.1 (7.5-13.1)	1.88 (1.76-1.99)
	Female	171.3 (97.8-282.1)	0.1 (0-0.1)	1402.6 (867.5-2101.3)	0.2 (0.1-0.3)	3.43 (3.3-3.56)	171.3 (97.8-282.1)	0.1 (0-0.1)	1402.6 (867.5-2101.3)	0.2 (0.1-0.3)	3.43 (3.3-3.56)
Southeast Asia	Both	811 (397.9-1417)	0.3 (0.2-0.6)	3278.1 (1816.2-5421.2)	0.5 (0.3-0.8)	1.14 (0.99-1.3)	21425.7 (10385.3-37679.7)	8.6 (4.1-15)	78329.2 (42444.8-128792.5)	11.6 (6.3-19)	0.82 (0.72-0.92)
	Male	596.8 (284.5-1057.8)	0.5 (0.2-0.9)	2367 (1298.7-3844.8)	0.7 (0.4-1.2)	1.08 (0.94-1.22)	15978 (7492.3-28529.2)	13.6 (6.4-24.3)	57902.3 (31731.5-94631.3)	17.8 (9.8-28.7)	0.81 (0.72-0.9)
	Female	214.2 (103-399.3)	0.2 (0.1-0.3)	911.1 (414-1604.5)	0.3 (0.1-0.4)	1.3 (1.07-1.53)	214.2 (103-399.3)	0.2 (0.1-0.3)	911.1 (414-1604.5)	0.3 (0.1-0.4)	1.3 (1.07-1.53)
Southern Latin America	Both	15.7 (4.8-33.6)	0 (0-0.1)	211.6 (71.3-386.5)	0.2 (0.1-0.4)	7.14 (6.91-7.36)	412.1 (127.9-888)	0.9 (0.3-1.9)	4959.9 (1787-9044.1)	5.7 (2.1-10.4)	6.76 (6.53-7)
	Male	11.9 (3.9-25.1)	0.1 (0-0.1)	146.9 (53.9-264.2)	0.3 (0.1-0.6)	6.81 (6.52-7.09)	314.9 (103.3-684)	1.5 (0.5-3.3)	3443.6 (1308.6-6230.9)	8.1 (3.1-14.6)	6.42 (6.13-6.72)
	Female	3.8 (1-8.2)	0 (0-0)	64.7 (14.4-127.1)	0.1 (0-0.3)	7.99 (7.7-8.29)	3.8 (1-8.2)	0 (0-0)	64.7 (14.4-127.1)	0.1 (0-0.3)	7.99 (7.7-8.29)
Southern Sub-Saharan Africa	Both	7.8 (3.3-15.6)	0 (0-0.1)	60.4 (32.5-103.4)	0.1 (0.1-0.2)	3.49 (2.78-4.21)	193 (78.2-380.3)	0.7 (0.3-1.5)	1461.5 (790.3-2541.6)	2.5 (1.4-4.4)	3.45 (2.76-4.15)
	Male	3.9 (1.3-8.6)	0 (0-0.1)	34 (17.9-57.8)	0.1 (0.1-0.2)	3.52 (2.61-4.43)	99.7 (30.6-218.1)	0.9 (0.3-1.9)	837.8 (442.2-1442.1)	3.3 (1.7-5.7)	3.45 (2.56-4.35)
	Female	3.9 (1.6-7.9)	0 (0-0.1)	26.4 (13-45.3)	0.1 (0-0.1)	3.53 (3.03-4.03)	3.9 (1.6-7.9)	0 (0-0.1)	26.4 (13-45.3)	0.1 (0-0.1)	3.53 (3.03-4.03)
Tropical Latin America	Both	82.4 (45.3-145.1)	0.1 (0.1-0.2)	760 (446.7-1119.9)	0.3 (0.2-0.4)	4.38 (4.12-4.64)	2149 (1155.9-3922.6)	2.4 (1.3-4.4)	17705.2 (10776.2-26044.3)	6.8 (4.1-9.9)	4.02 (3.75-4.29)
	Male	45.3 (24.1-78.9)	0.1 (0.1-0.2)	455.2 (274-661.8)	0.4 (0.2-0.5)	4.89 (4.59-5.19)	1193.6 (620.7-2079.1)	2.9 (1.5-5)	10798.3 (6558.7-15551.3)	8.8 (5.3-13)	4.56 (4.25-4.87)
	Female	37.1 (20.5-64.9)	0.1 (0-0.1)	304.8 (178.9-463.5)	0.2 (0.1-0.3)	3.73 (3.51-3.95)	37.1 (20.5-64.9)	0.1 (0-0.1)	304.8 (178.9-463.5)	0.2 (0.1-0.3)	3.73 (3.51-3.95)
Western Europe	Both	2132.9 (1284.1-3193.1)	0.4 (0.2-0.6)	10006.5 (6836-13273.3)	1.1 (0.8-1.5)	3.52 (3.34-3.71)	53954.5 (32798.4-81451.5)	9.4 (5.7-14.2)	200848.7 (139424-264210.6)	22.8 (15.8-29.9)	2.75 (2.58-2.92)
	Male	1748.1 (1046.6-2620.3)	0.7 (0.4-1.1)	7445.3 (5094.7-9908.5)	1.7 (1.2-2.3)	2.87 (2.68-3.06)	44804.5 (27053.6-67619.3)	18.2 (11-27.5)	150066.6 (106005.1-198106.9)	35.6 (24.9-47.1)	2.07 (1.89-2.26)
	Female	384.8 (224.5-588)	0.1 (0.1-0.2)	2561.2 (1686.2-3672)	0.5 (0.4-0.8)	5.2 (5.01-5.38)	384.8 (224.5-588)	0.1 (0.1-0.2)	2561.2 (1686.2-3672)	0.5 (0.4-0.8)	5.2 (5.01-5.38)
Western Sub-Saharan Africa	Both	40.4 (17.6-80)	0 (0-0.1)	154.7 (78.2-270.8)	0.1 (0-0.1)	1.5 (1.39-1.61)	1014.3 (444.8-2027.6)	1.2 (0.5-2.4)	3727.5 (1868.9-6540.2)	2 (1-3.4)	1.35 (1.25-1.46)
	Male	21.5 (9.2-41.2)	0 (0-0.1)	76.2 (36.2-141.5)	0.1 (0-0.1)	1.62 (1.5-1.74)	560.9 (240.2-1085.5)	1.3 (0.5-2.5)	1874.8 (856.8-3482.3)	2 (0.9-3.6)	1.42 (1.3-1.54)
	Female	18.9 (7.4-43)	0 (0-0.1)	78.5 (41.2-132.8)	0.1 (0-0.1)	1.4 (1.29-1.51)	18.9 (7.4-43)	0 (0-0.1)	78.5 (41.2-132.8)	0.1 (0-0.1)	1.4 (1.29-1.51)

### 3.3 Country burden of drug-induced liver cancer in elderly patients

In 2021, there were significant regional differences in the number of drug-induced liver cancer deaths among elderly patients worldwide. China, the United States, and Japan had the highest death tolls, with China reporting 17,460 (95% CI: 10,989.3–24,425.2) deaths, the United States 8,652 (95% CI: 7,223.8–10,059.6) deaths, and Japan 4,457 (95% CI: 2,893.0–6,327.8) deaths (**Appendix pp. 1–11**). However, when examining the age-standardized mortality rates (ASMR), the situation differed. Mongolia, Tonga, and Spain had the highest ASMRs, with rates of 12.2 per 100,000 (95% CI: 0.7–25.6), 2.7 per 100,000 (95% CI: 0.3–4.8), and 2.0 per 100,000 (95% CI: 0.4–2.9), respectively. Conversely, Uganda, São Tomé and Príncipe, and Bangladesh had the lowest rates, with values of 0.005 per 100,000 (95% CI: 0.00–0.01), 0.02 per 100,000 (95% CI: 0.00–0.05), and 0.02 per 100,000 (95% CI: 0.00–0.05) (**Appendix pp. 12–26**).

**Figure 2:**
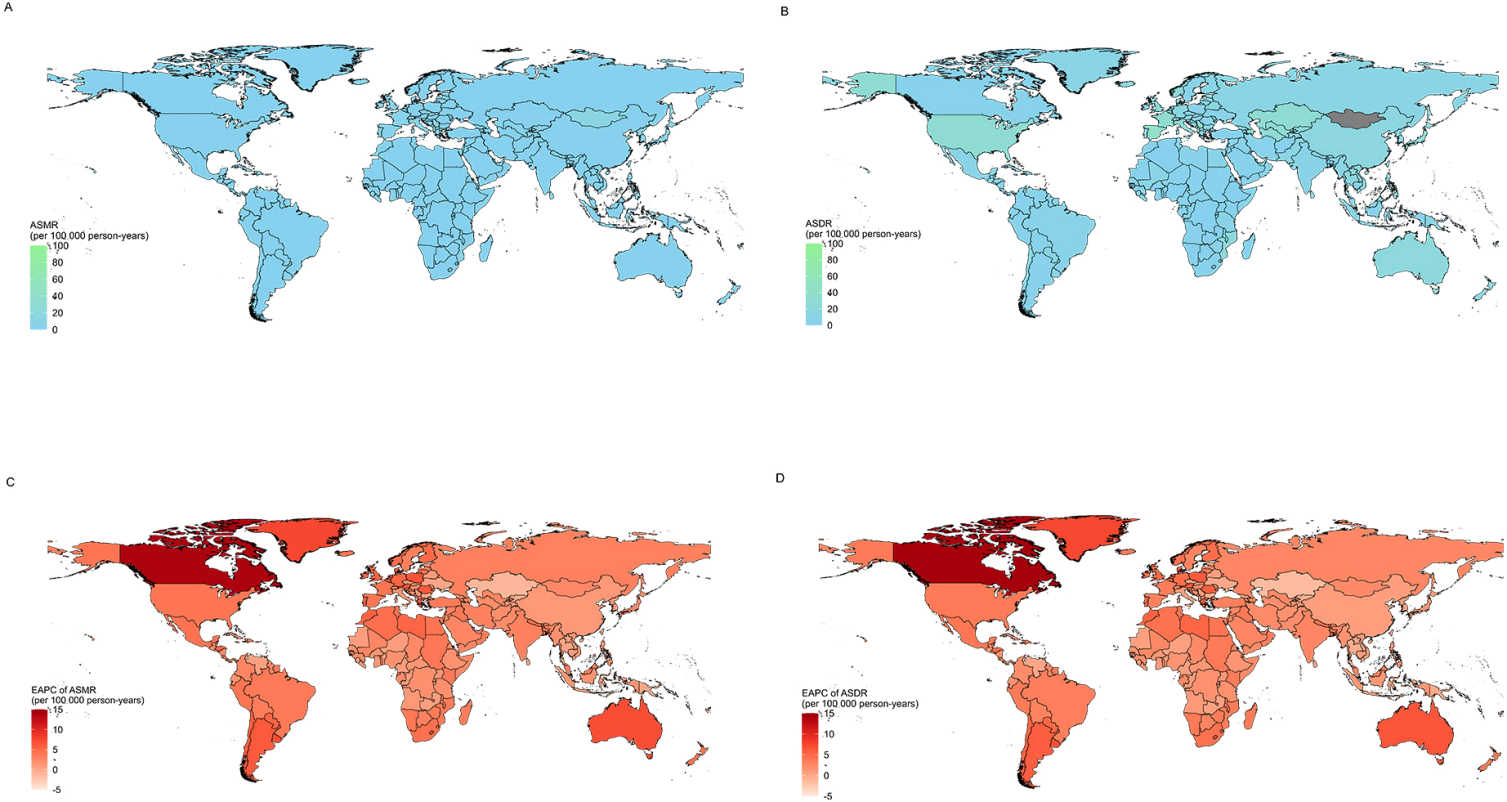
Country trends of drug-induced liver cancer in elderly patients; **(A)** Age-standardized incidence rate in 2021; **(B)** Age-standardized DALYs rate in 2021; **(C)** EAPC of ASMR from 1990 to 2021; **(D)** EAPC of ASDR from 1990 to 2021;EAPC, estimated annual percentage change.

In terms of drug-induced liver cancer DALYs in elderly patients, significant differences were also observed across countries. In 2021, China, the United States, and India had the highest DALY counts, with China recording 383,709 (95% CI: 246,147.3–533,510.4) DALYs, the United States 186,618 (95% CI: 156,865.6–218,054.2) DALYs, and India 89,570 (95% CI: 64,317.9–119,720.7) DALYs (**Appendix pp. 27–50**). Mongolia, Tonga, and Spain had the highest age-standardized DALY rates, with rates of 312 per 100,000 (95% CI: 20.7–599.9), 62 per 100,000 (95% CI: 8.3–108.5), and 40 per 100,000 (95% CI: 9.1–55.7), respectively. In contrast, Uganda, São Tomé and Príncipe, and Bangladesh had lower age-standardized DALY rates, with values of 0.12 per 100,000 (95% CI: 0.01–0.30), 0.40 per 100,000 (95% CI: 0.05–1.11), and 0.60 per 100,000 (95% CI: 0.02–1.40), respectively (**Figure 2B**, **Appendix pp. 51–69**).

Between 1990 and 2021, among 204 countries and territories, 198 countries and territories saw an increase in age-standardized mortality rates due to drug-induced liver cancer in elderly patients. The largest increase was observed in Canada, where the rate rose from 0.002 (95% CI: 0.0002–0.004) per 100,000 in 1990 to 0.130 (95% CI: 0.019–0.265) per 100,000 in 2021, with an EAPC of 13.82 (95% CI: 9.01–18.84). Conversely, only six countries and territories showed a downward trend in age-standardized mortality rates. However, Mauritius was the only country where this decrease was statistically significant. In Mauritius, the rate decreased from 0.576 (95% CI: 0.086–1.003) per 100,000 in 1990 to 0.141 (95% CI: 0.030–0.209) per 100,000 in 2021, with an EAPC of -4.44 (95% CI: -4.68 to -4.20). Notably, despite the generally lower economic development in the African region, Mauritius stands out as a high-income outlier, and its relatively better healthcare system may be a key factor contributing to this favorable trend (**Figure 2A**, **Appendix pp. 70–84**). The other five countries showing non-significant downward trends included Kuwait (EAPC: -1.46 [95% CI: -5.04 to 2.26]), Kazakhstan (EAPC: -1.12 [95% CI: -3.14 to 0.94]), Seychelles (EAPC: -0.35 [95% CI: -3.52 to 2.92]), Kyrgyzstan (EAPC: -0.19 [95% CI: -1.99 to 1.64]), and Bulgaria (EAPC: -0.01 [95% CI: -1.20 to 1.19]).

In 196 of the countries and territories, the age-standardized DALY rate due to drug-induced liver cancer in elderly patients also showed an upward trend. The largest increase was again observed in Canada, where the rate rose from 0.04 (95% CI: 0.003–0.10) per 100,000 in 1990 to 3.52 (95% CI: 0.50–7.06) per 100,000 in 2021, with an EAPC of 14.27 (95% CI: 9.35–19.42). Conversely, only eight countries and territories showed a downward trend in age-standardized DALY rates. Similar to the mortality trends, Mauritius was the only country to exhibit a statistically significant decrease. In Mauritius, the age-standardized DALY rate decreased from 13.86 (95% CI: 2.20–22.76) per 100,000 in 1990 to 3.16 (95% CI: 0.76–4.71) per 100,000 in 2021, with an EAPC of -4.66 (95% CI: -5.08 to -4.24). The other seven countries showing non-significant downward trends included Kuwait (EAPC: -1.87 [95% CI: -5.64 to 2.04]), Kazakhstan (EAPC: -1.47 [95% CI: -3.62 to 0.73]), Maldives (EAPC: -0.72 [95% CI: -4.42 to 3.12]), Bulgaria (EAPC: -0.55 [95% CI: -1.81 to 0.72]), Seychelles (EAPC: -0.52 [95% CI: -3.96 to 3.03]), Kyrgyzstan (EAPC: -0.41 [95% CI: -2.35 to 1.57]), and Thailand (EAPC: -0.23 [95% CI: -3.16 to 2.79]) (**Figure 2**; **Appendix pp. 85–99**).

### 3.4 The global trend of drug-induced liver cancer in elderly patients mortality and DALYs based on gender

From 1990 to 2021, the age-standardized mortality rates and DALY rates due to drug-induced liver cancer in elderly patients globally showed a consistent upward trend. In terms of gender differences, males generally had higher rates than females, with the relevant indicators in more than half of the global regions being more than twice as high in males as in females. Among females, the increase in age-standardized mortality and DALY rates was relatively slower.

The High-income Asia Pacific region exhibited the most substantial gender disparity in these burdens. In this region, the age-standardized mortality and DALY rates for males were the highest globally, whereas the rates for females remained relatively low. By 2021, the age-standardized mortality rate for males in this region was approximately 10 times that of females, at 2.4 per 100,000 (95% CI: 1.6–3.4) for males compared to 0.2 per 100,000 (95% CI: 0.1–0.4) for females. Meanwhile, the DALY rate for males was 10.5 times higher than that for females, with rates of 49.2 per 100,000 (95% CI: 32.7–68.4) for males and 4.7 per 100,000 (95% CI: 2.7–7.3) for females.

**Figure 3:**
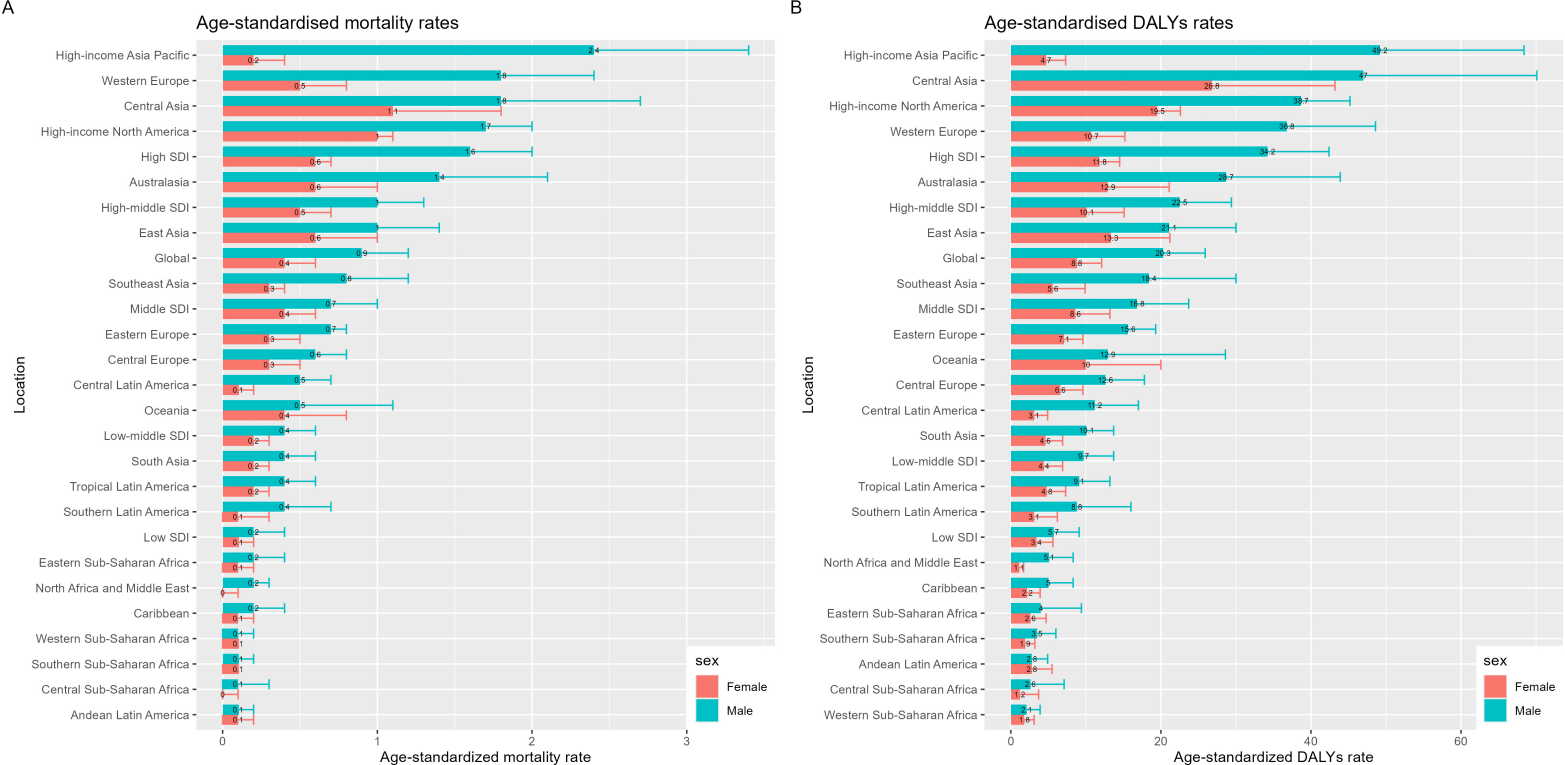
Age-specific mortality rates and contributions of drug-induced liver cancer in elderly patients by SDI quintile (1990–2021). **(A)** Contribution of Drug-Induced Liver Cancer in Elderly Patients deaths 1900 by country; **(B)** Contribution of Drug-Induced Liver Cancer in Elderly Patients Deaths 2021 by country.

In contrast, in Western Sub-Saharan Africa, and Andean Latin America regions, the age-standardized mortality rates for males and females were nearly identical. Additionally, in the Andean Latin America region, the age-standardized DALY rates for males and females were also similar (**Figure 3**). These regional variations suggest that factors such as healthcare disparities, gender-specific behavioral patterns regarding drug use, and regional health policies may significantly influence the liver cancer burden in elderly patients.

### 3.5 Country burden of drug-induced liver cancer in elderly patients on age group

**Figure 4:**
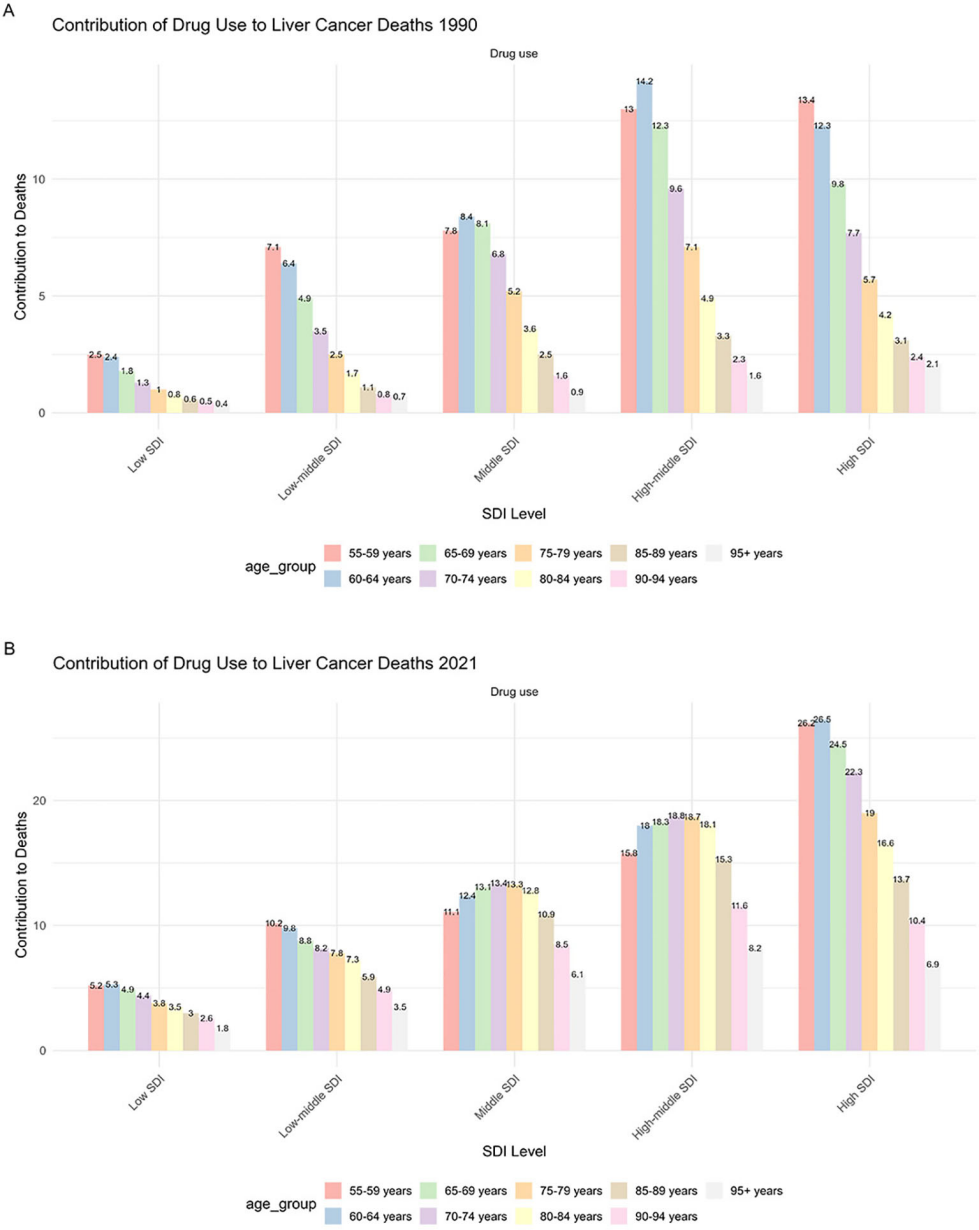
**(A)** Mortality rate of drug-induced liver cancer in elderly patients by sex; **(B)** DALYs rate of drug-induced liver cancer in elderly patients by sex.

From 1990 to 2021, High SDI countries consistently exhibited the highest mortality rates among elderly patients. The burden in these regions was predominantly concentrated in the 55–74 age group (**Figure 4**), with a distinct peak contribution observed in the 60–64 age group. In contrast, the contribution in the oldest age group (95+ years) was minimal. Low SDI countries showed the lowest overall mortality rates, characterized by a relatively flat distribution of burden across the 55–74 age groups. Notably, a significant disparity was observed in the oldest elderly population (95+ years): the mortality rate in High-middle SDI countries was approximately 4.6 times higher than that in Low SDI countries. Globally, while mortality rates for patients aged 70 and above have shown a decreasing trend over the past three decades, the absolute burden continues to rise due to population aging.

In terms of general age and gender patterns (**Supplementary Figure S2**), the 55–59 age group represents a critical turning point where both mortality and DALY rates begin to surge, eventually reaching a global peak in the 65–69 age group. This trend is consistent across both sexes; however, male mortality rates remain significantly higher than those of females, and the decline in DALY rates among older males is notably slower than in females (**Supplementary Figure S3**).

## 4 Discussion

### 4.1 Global temporal trends and socioeconomic disparities

To the best of our knowledge, this study provides the most comprehensive assessment of the global liver cancer burden attributable to drug use in the elderly (1990–2021), revealing a persistent upward trajectory. Crucially, our analysis uncovers a divergence between burden intensity and temporal trends. While the absolute mortality burden demonstrates a positive association with the Socio-demographic Index (SDI)—concentrating heavily in affluent regions—the pace of escalation follows a distinct U-shaped pattern, with rapid accelerations occurring in both high- and low-SDI settings. Compounding these socioeconomic disparities is a profound demographic divide: particularly in high-income regions, male mortality rates significantly exceed those of females. These results highlight a complex epidemiological landscape driven by the intersection of illicit substance use, viral hepatitis, and metabolic aging.

### 4.2 Synergistic drivers: viral sequelae, metabolic dysfunction, and iatrogenic injury

The clinical significance of our findings lies in the distinct etiology of the burden, which originates from the severe sequelae of opioid dependence and injection drug use (IDU) but is significantly amplified by the physiological vulnerabilities of aging. This progression follows a lethal “toxic cascade.” First, unsafe injection practices represent a historical pathogenic legacy: the “cohort effect” of past injection drug use has resulted in decades of transmission of blood-borne viruses. While Hepatitis C (HCV) remains the predominant driver in this demographic, the contribution of Hepatitis B (HBV)—and particularly HCV-HBV coinfection—cannot be overlooked. These viral pathogens act as synergistic accelerators of carcinogenesis, compounding the direct hepatotoxicity of opioid dependence (21–23). Second, the management of opioid dependence and its associated comorbidities introduces a secondary “Iatrogenic Hit”. Older individuals with a history of substance use disproportionately suffer from HIV coinfection or bacterial complications, necessitating the prolonged use of hepatotoxic medications—specifically anti-tuberculosis agents, antibiotics, or antiretrovirals. In the aging liver, the biotransformation of these drugs generates reactive oxygen species (ROS) that accumulate due to declining detoxification capacity, causing persistent oxidative DNA damage (24–26). Crucially, this drug-induced volatility is catalyzed by metabolic dysfunction (27). As highlighted by Caturano et al. (28), the hyperinsulinemia associated with insulin resistance activates the PI3K-AKT pathway, forcing these chemically damaged cells to proliferate rather than repair. Therefore, effective clinical management must transcend traditional oncology and adopt a multidisciplinary approach. We recommend a three-pronged strategy: (1) Source Control: Integrating Opioid Substitution Therapy (OST) with aggressive Direct-Acting Antiviral (DAA) treatment to eliminate the viral driver; (2) Hepatotoxicity Stewardship: Implementing strict medication reconciliation to minimize the use of non-essential hepatotoxic antibiotics and analgesics; and (3) Metabolic & Lifestyle Intervention: Prioritizing broad lifestyle modifications—such as dietary optimization and physical activity—for the general elderly population to improve metabolic homeostasis and attenuate pro-oncogenic signaling.

### 4.3 Gender disparity: biological susceptibility and iatrogenic exposure

A salient epidemiological hallmark unmasked by this analysis is the stark sexual dichotomy in mortality burden. In high-risk zones like the High-income Asia Pacific, male mortality rates exceed those of females by approximately tenfold, a disparity that warrants a deep exploration of physiological and environmental drivers. Physiologically, this gap is underpinned by sexual dimorphism in metabolic and hormonal regulation (29, 30). The male liver exhibits higher metabolic activity but lower resilience to toxic insults. Hormonal regulation plays a pivotal role: androgens promote hepatocyte proliferation, whereas estrogens exert a protective effect by mitigating drug-induced injury (31, 32). Crucially, a study by Alessandro Allegra et al (33). underscores that differences in antioxidant enzyme activity are a significant determinant; males typically exhibit lower antioxidant capacity, making their hepatocytes more susceptible to drug-induced oxidative stress. Immunologically, the disparity is equally striking. Research by Sabra L. Klein (34) and colleagues demonstrates that male immune responses are generally more vulnerable than those of females, leading to higher susceptibility to infections. This susceptibility is epitomized by tuberculosis (TB), where the notification rate is typically twice as high in males (35). This higher infectious burden necessitates the prolonged use of hepatotoxic anti-TB regimens (e.g., isoniazid), directly increasing the cumulative risk of drug-induced liver carcinogenesis in males via iatrogenic exposure. Synergizing with these biological factors are lifestyle and metabolic risks (36). Males historically have higher rates of smoking and alcohol consumption, which drive underlying comorbidities (37). Moreover, studies indicate that male dietary habits are often less healthy, characterized by a higher intake of salt, sugar, and fat, and a lower consumption of protein and fiber (38). These dietary patterns induce metabolic dysfunction, rendering the liver “fragile” and significantly amplifying the tumorigenic potential of hepatotoxic drugs.

### 4.4 Age-specific distribution and high-risk populations

Complementing the gender dichotomy is a distinct age-dependent susceptibility pattern, culminating in the 55–74 cohort—a demographic we designate as the “active intervention window”. This pronounced peak reflects a “Pharmacological Paradox” inherent to modern geriatric care: while aggressive pharmacotherapy successfully extends life expectancy, it simultaneously exposes the aging liver to cumulative hepatotoxicity (39). Unlike the 95+ age group, where therapeutic conservatism limits exposure, the 55–74 cohort is frequently subjected to intensive regimens. Mechanistically, this vulnerability is driven by the hepatotoxicity of common geriatric pharmacotherapies. First, broad-spectrum antibiotics and long-term analgesics (e.g., NSAIDs)—routinely prescribed for age-related infections and Chronic pain syndromes—act as occult inducers of oxidative stress (24, 40). In the aging liver, the biotransformation of these agents generates excessive reactive oxygen species (ROS) that exceed the capacity of diminishing antioxidant defenses. This leads to persistent oxidative damage to hepatocyte DNA, creating a mutagenic environment that favors *de novo* oncogenesis (41). Second, the long-term use of corticosteroids for autoimmune conditions can induce hepatic steatosis. This steroid-induced fatty liver creates a pro-inflammatory microenvironment that, over time, progresses to cirrhosis and malignancy (42–44). To mitigate this iatrogenic risk, clinical protocols must evolve. We advocate for: (1) Integrating Comprehensive Geriatric Assessment (CGA) to evaluate hepatic reserve before initiating high-risk regimens; (2) Implementing “Active Deprescribing” to systematically taper non-essential hepatotoxic drugs; and (3) Mandating enhanced liver function monitoring for patients on unavoidable high-risk therapies to detect toxicity before irreversible damage occurs.

### 4.5 Regional heterogeneity: divergent drivers and stratified mitigation strategies

Finally, the regional heterogeneity in disease burden unveils a “Divergent Driver Hypothesis” across the socioeconomic spectrum. From a macro perspective, Low and Middle-SDI regions are characterized by a “Crisis of Unmet Needs” and “Unmonitored Toxicity.” In these resource-constrained settings, limited healthcare infrastructure often precludes routine toxicological monitoring, implying that drug-induced liver injury is frequently superimposed on an unmanaged viral hepatitis background (45, 46). This complex dynamic is most critically illustrated by the case of Mongolia. Although the country is estimated to have the highest age-standardized mortality rate globally (12.2/100k), the estimate exhibits a substantial statistical disparity: the 95% confidence interval is exceptionally wide (0.7–25.6/100,000). This numerical instability points to a deeper underlying cause: the “Small Population Bias” compounded by a scarcity of robust drug surveillance data (47, 48). Therefore, while the *exact magnitude* is statistically uncertain, the *signal* is clinically valid, reflecting the severe reality of hyper-endemic HDV/HCV co-infection lacking adequate toxicological support. To mitigate risks in such settings, we propose a strategy of “Resource Integration”: specifically, integrating mandatory liver function testing (LFT) into existing national infectious disease control programs (e.g., the DOTS framework for TB) to capture toxicity early without extensive new infrastructure.

In stark contrast, the rapid escalation in High-SDI regions (e.g., Canada) is iatrogenic, driven by “Over-medicalization” and the “Modern Opioid Crisis.” Here, advanced healthcare systems paradoxically facilitate an “Accessibility Paradox”: widespread opioid availability, combined with illicit injection drug use, fuels a resurgence of HCV-related liver cancer (11, 39, 49). Furthermore, the extensive use of polypharmacy in the elderly creates a “saturated” metabolic environment. Consequently, management in these regions must shift from access to “Proactive Prevention.” We recommend implementing Electronic Health Record (EHR)-based pharmacovigilance to trigger automated alerts for high-risk drug combinations, thereby facilitating the “active deprescribing” of non-essential medications to flatten the rising curve.

## 5 Strengths and limitations

This study provides the most comprehensive stratification of the global liver cancer burden attributable to drug use in the elderly. Spanning three decades, our analysis fills a critical gap in geriatric epidemiology by delineating the “active intervention window” (55–74 years) and the “SDI Divergence,” offering a robust framework for precision prevention. However, inherent limitations exist. Regional disparities in surveillance quality may lead to underreporting, particularly in lower-SDI settings, while the lack of pharmacological granularity precludes distinguishing between illicit and prescribed agents. Furthermore, as an aggregate analysis, findings are subject to ecological fallacy and data lags, necessitating clinical validation of specific causal mechanisms. Nonetheless, these constraints do not undermine the study’s utility in establishing a critical global baseline for strategic planning.

## 6 Conclusion

In summary, the escalating burden of drug-induced liver cancer in the elderly is not merely a demographic inevitability but a reflection of the “Pharmacological Paradox”: while modern medicine successfully manages chronic multimorbidity, the resulting necessity for prolonged, complex medication regimens imposes a cumulative strain on the aging liver. Our study highlights a critical divergence: the burden is driven by “unmet needs” and viral endemicity in lower-SDI regions, whereas it is fueled by the challenges of “therapeutic complexity” and polypharmacy in high-SDI nations. Therefore, mitigating this global crisis requires a paradigm shift from passive treatment to proactive stewardship. We advocate for a stratified intervention strategy that integrates liver function screening into infectious disease programs in resource-limited settings, while implementing strict pharmacovigilance and “active deprescribing” protocols in developed healthcare systems. Ultimately, future efforts must leverage pharmacogenomics to personalize medication management, ensuring that the benefits of chronic disease management are balanced with the preservation of liver health in the aging population.

## Data Availability

The original contributions presented in the study are included in the article/**Supplementary Material**. Further inquiries can be directed to the corresponding author.
