# Supplementary figures and images for: Global and regional burden of liver cancer attributable to drug use in elderly patients: a 1990–2021 analysis from the GBD study
Source: Front Oncol. 2026 Feb 24;16:1678700. doi: 10.3389/fonc.2026.1678700 (PMC12971456; doi:10.3389/fonc.2026.1678700)

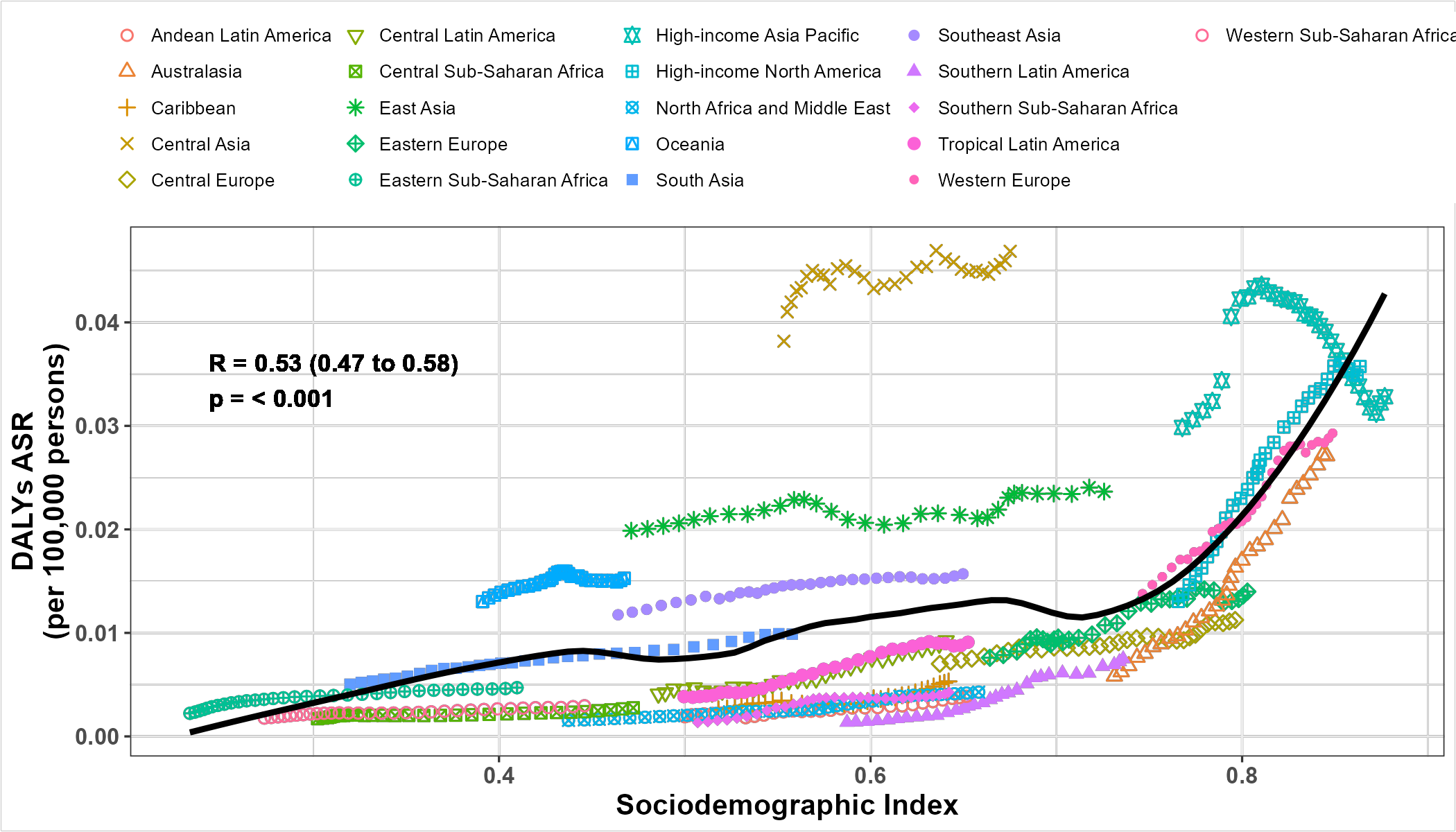

Supplement: Supplementary file 1 [file Image1.tiff]

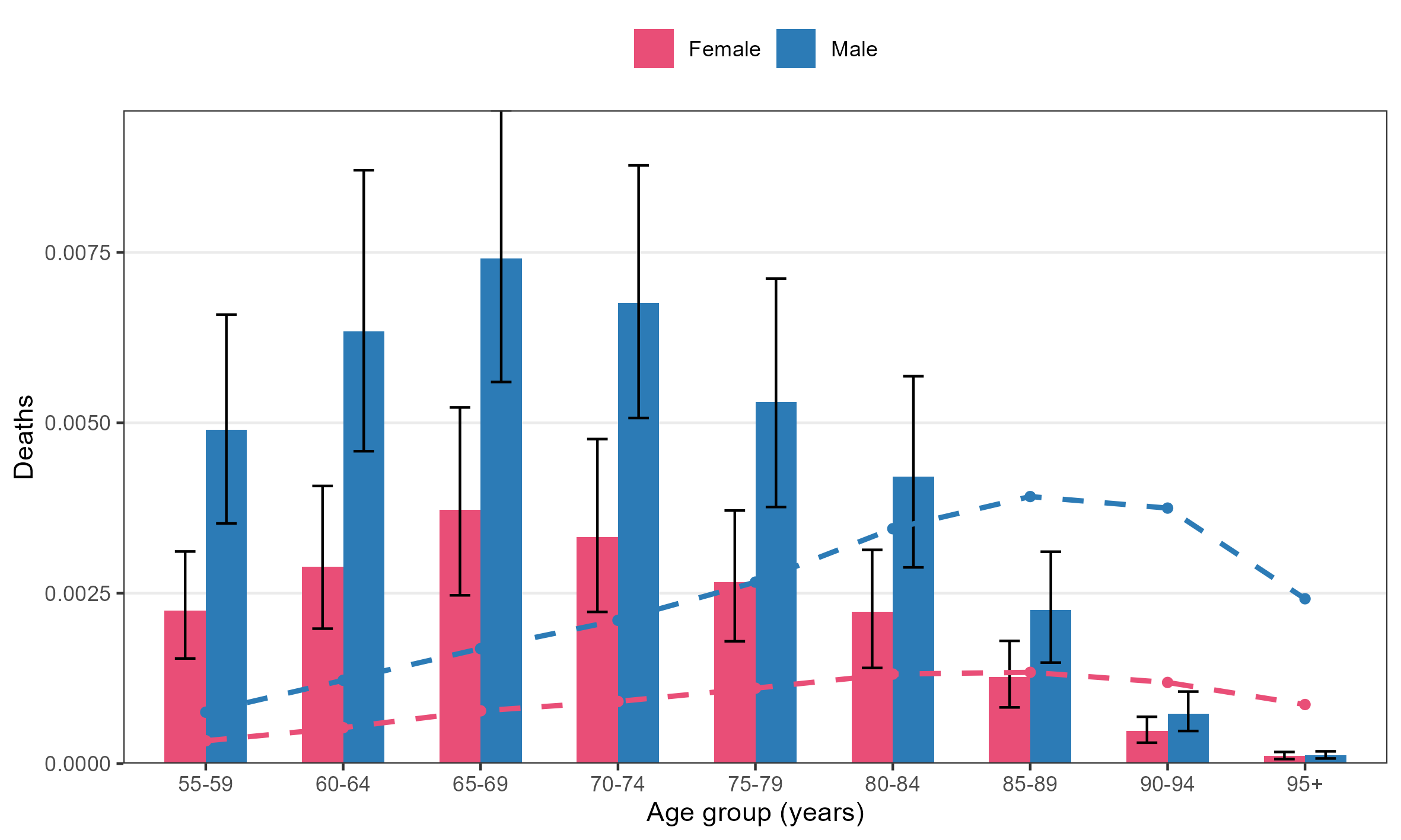

Supplement: Supplementary file 2 [file Image2.tiff]

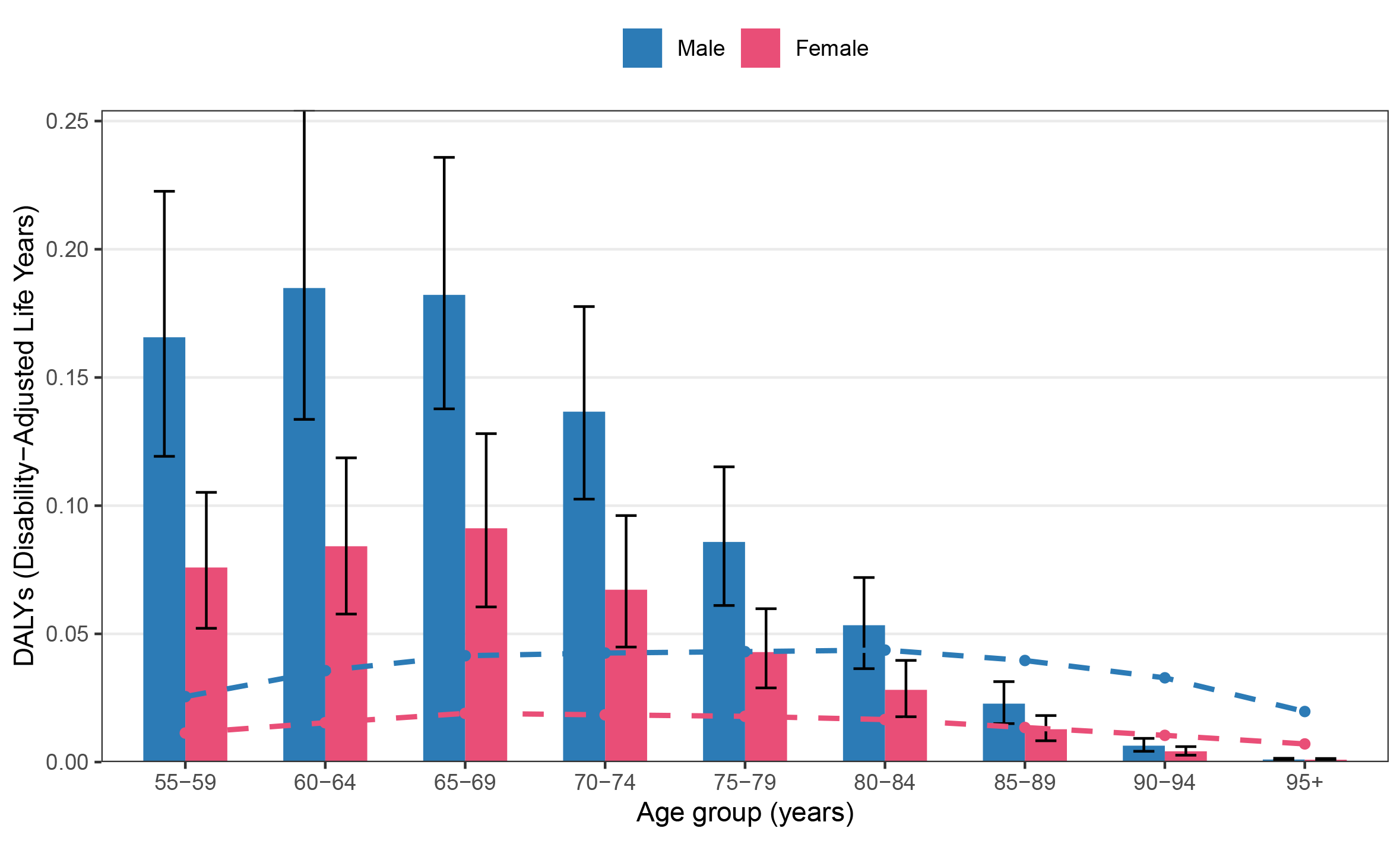

Supplement: Supplementary file 3 [file Image3.tif]
