# Supplementary material for: Global and regional burden of liver cancer attributable to drug use in elderly patients: a 1990–2021 analysis from the GBD study
Source: Front Oncol. 2026 Feb 24;16:1678700. doi: 10.3389/fonc.2026.1678700 (PMC12971456; doi:10.3389/fonc.2026.1678700)
Supplement: Supplementary file 4 [file Table1.docx]

Appendix

| measure | location | sex | age | cause | rei | metric | year | val | upper | lower |
| --- | --- | --- | --- | --- | --- | --- | --- | --- | --- | --- |
| Deaths | People's Republic of China | Both | 55+ years | Liver cancer | Drug use | Number | 2021 | 17460.22 | 24425.16 | 10989.3 |
| Deaths | United States of America | Both | 55+ years | Liver cancer | Drug use | Number | 2021 | 8652.23 | 10059.64 | 7223.818 |
| Deaths | Japan | Both | 55+ years | Liver cancer | Drug use | Number | 2021 | 4456.546 | 6327.796 | 2893.04 |
| Deaths | Republic of India | Both | 55+ years | Liver cancer | Drug use | Number | 2021 | 3743.594 | 4982.66 | 2676.414 |
| Deaths | French Republic | Both | 55+ years | Liver cancer | Drug use | Number | 2021 | 2261.08 | 3509.347 | 205.2634 |
| Deaths | Kingdom of Spain | Both | 55+ years | Liver cancer | Drug use | Number | 2021 | 1808.561 | 2632.118 | 370.2994 |
| Deaths | United Kingdom of Great Britain and Northern Ireland | Both | 55+ years | Liver cancer | Drug use | Number | 2021 | 1754.451 | 2187.408 | 1332.192 |
| Deaths | Republic of Italy | Both | 55+ years | Liver cancer | Drug use | Number | 2021 | 1664.628 | 2279.836 | 1084.908 |
| Deaths | Federal Republic of Germany | Both | 55+ years | Liver cancer | Drug use | Number | 2021 | 1370.246 | 2355.849 | 249.4018 |
| Deaths | Russian Federation | Both | 55+ years | Liver cancer | Drug use | Number | 2021 | 1319.139 | 1645.951 | 968.7774 |
| Deaths | Socialist Republic of Viet Nam | Both | 55+ years | Liver cancer | Drug use | Number | 2021 | 969.7134 | 1911.899 | 99.80686 |
| Deaths | Kingdom of Thailand | Both | 55+ years | Liver cancer | Drug use | Number | 2021 | 896.8884 | 1647.039 | 85.7131 |
| Deaths | Federative Republic of Brazil | Both | 55+ years | Liver cancer | Drug use | Number | 2021 | 733.1124 | 1079.109 | 428.7066 |
| Deaths | Republic of Korea | Both | 55+ years | Liver cancer | Drug use | Number | 2021 | 652.6278 | 1299.973 | 110.3066 |
| Deaths | Islamic Republic of Pakistan | Both | 55+ years | Liver cancer | Drug use | Number | 2021 | 578.4166 | 1124.916 | 195.3199 |
| Deaths | Republic of the Philippines | Both | 55+ years | Liver cancer | Drug use | Number | 2021 | 471.1368 | 661.8679 | 309.844 |
| Deaths | United Mexican States | Both | 55+ years | Liver cancer | Drug use | Number | 2021 | 458.284 | 697.1594 | 270.7591 |
| Deaths | Republic of Indonesia | Both | 55+ years | Liver cancer | Drug use | Number | 2021 | 457.8428 | 881.3113 | 201.2002 |
| Deaths | Australia | Both | 55+ years | Liver cancer | Drug use | Number | 2021 | 437.7639 | 717.9729 | 17.91559 |
| Deaths | Taiwan (Province of China) | Both | 55+ years | Liver cancer | Drug use | Number | 2021 | 353.7194 | 770.6119 | 23.21956 |
| Deaths | Republic of Uzbekistan | Both | 55+ years | Liver cancer | Drug use | Number | 2021 | 303.3156 | 503.2003 | 20.14894 |
| Deaths | Republic of Serbia | Both | 55+ years | Liver cancer | Drug use | Number | 2021 | 302.2143 | 469.7493 | 74.55244 |
| Deaths | Mongolia | Both | 55+ years | Liver cancer | Drug use | Number | 2021 | 284.9144 | 550.1102 | 17.13905 |
| Deaths | Republic of Kazakhstan | Both | 55+ years | Liver cancer | Drug use | Number | 2021 | 251.9894 | 372.1671 | 38.95272 |
| Deaths | Republic of the Union of Myanmar | Both | 55+ years | Liver cancer | Drug use | Number | 2021 | 232.873 | 542.4633 | 12.68223 |
| Deaths | Swiss Confederation | Both | 55+ years | Liver cancer | Drug use | Number | 2021 | 193.3533 | 292.6436 | 35.9954 |
| Deaths | Islamic Republic of Iran | Both | 55+ years | Liver cancer | Drug use | Number | 2021 | 191.1251 | 260.7454 | 131.1972 |
| Deaths | Democratic People's Republic of Korea | Both | 55+ years | Liver cancer | Drug use | Number | 2021 | 185.045 | 375.979 | 12.02377 |
| Deaths | Ukraine | Both | 55+ years | Liver cancer | Drug use | Number | 2021 | 177.3907 | 299.4564 | 56.90965 |
| Deaths | Portuguese Republic | Both | 55+ years | Liver cancer | Drug use | Number | 2021 | 171.0163 | 305.1813 | 13.47397 |
| Deaths | Republic of Poland | Both | 55+ years | Liver cancer | Drug use | Number | 2021 | 169.4789 | 222.3633 | 118.3248 |
| Deaths | Republic of Azerbaijan | Both | 55+ years | Liver cancer | Drug use | Number | 2021 | 166.6258 | 358.9316 | 14.66314 |
| Deaths | Arab Republic of Egypt | Both | 55+ years | Liver cancer | Drug use | Number | 2021 | 159.2583 | 352.6296 | 10.56892 |
| Deaths | Republic of Chile | Both | 55+ years | Liver cancer | Drug use | Number | 2021 | 137.0873 | 261.3033 | 9.7643 |
| Deaths | Kingdom of Belgium | Both | 55+ years | Liver cancer | Drug use | Number | 2021 | 133.6932 | 251.4838 | 8.532509 |
| Deaths | Malaysia | Both | 55+ years | Liver cancer | Drug use | Number | 2021 | 123.2729 | 231.4661 | 11.80096 |
| Deaths | Republic of Belarus | Both | 55+ years | Liver cancer | Drug use | Number | 2021 | 121.3319 | 183.8923 | 45.2603 |
| Deaths | Kingdom of the Netherlands | Both | 55+ years | Liver cancer | Drug use | Number | 2021 | 108.3528 | 195.8389 | 10.79314 |
| Deaths | Republic of Mozambique | Both | 55+ years | Liver cancer | Drug use | Number | 2021 | 99.64076 | 276.2686 | 6.948778 |
| Deaths | Kingdom of Norway | Both | 55+ years | Liver cancer | Drug use | Number | 2021 | 96.78664 | 124.7905 | 68.06581 |
| Deaths | Canada | Both | 55+ years | Liver cancer | Drug use | Number | 2021 | 94.38116 | 192.4536 | 13.54035 |
| Deaths | Romania | Both | 55+ years | Liver cancer | Drug use | Number | 2021 | 93.80359 | 190.4544 | 5.754902 |
| Deaths | Republic of Colombia | Both | 55+ years | Liver cancer | Drug use | Number | 2021 | 88.00603 | 181.4828 | 7.05137 |
| Deaths | Bolivarian Republic of Venezuela | Both | 55+ years | Liver cancer | Drug use | Number | 2021 | 77.07056 | 159.4598 | 4.460193 |
| Deaths | Federal Democratic Republic of Nepal | Both | 55+ years | Liver cancer | Drug use | Number | 2021 | 73.40794 | 158.7831 | 9.241364 |
| Deaths | Republic of Turkey | Both | 55+ years | Liver cancer | Drug use | Number | 2021 | 71.66286 | 167.7375 | 7.17988 |
| Deaths | United Republic of Tanzania | Both | 55+ years | Liver cancer | Drug use | Number | 2021 | 68.67351 | 150.6778 | 7.600174 |
| Deaths | State of Israel | Both | 55+ years | Liver cancer | Drug use | Number | 2021 | 68.11563 | 115.0119 | 6.408913 |
| Deaths | Republic of Austria | Both | 55+ years | Liver cancer | Drug use | Number | 2021 | 68.00023 | 131.2122 | 8.142786 |
| Deaths | Argentine Republic | Both | 55+ years | Liver cancer | Drug use | Number | 2021 | 66.12391 | 133.262 | 5.639273 |
| Deaths | Hellenic Republic | Both | 55+ years | Liver cancer | Drug use | Number | 2021 | 63.33536 | 122.4125 | 8.7061 |
| Deaths | Kingdom of Sweden | Both | 55+ years | Liver cancer | Drug use | Number | 2021 | 63.3102 | 114.8442 | 20.68522 |
| Deaths | Republic of Tajikistan | Both | 55+ years | Liver cancer | Drug use | Number | 2021 | 63.22174 | 125.5479 | 6.127158 |
| Deaths | New Zealand | Both | 55+ years | Liver cancer | Drug use | Number | 2021 | 60.04852 | 82.65814 | 21.62868 |
| Deaths | Czech Republic | Both | 55+ years | Liver cancer | Drug use | Number | 2021 | 58.69708 | 105.6907 | 9.360708 |
| Deaths | Republic of Bulgaria | Both | 55+ years | Liver cancer | Drug use | Number | 2021 | 54.68981 | 103.1291 | 8.863793 |
| Deaths | Georgia | Both | 55+ years | Liver cancer | Drug use | Number | 2021 | 53.03979 | 85.89573 | 6.935529 |
| Deaths | Republic of Finland | Both | 55+ years | Liver cancer | Drug use | Number | 2021 | 53.02132 | 109.8577 | 6.064008 |
| Deaths | Ireland | Both | 55+ years | Liver cancer | Drug use | Number | 2021 | 48.67147 | 82.62018 | 6.227121 |
| Deaths | Republic of Albania | Both | 55+ years | Liver cancer | Drug use | Number | 2021 | 48.15748 | 97.14812 | 4.485681 |
| Deaths | Kyrgyz Republic | Both | 55+ years | Liver cancer | Drug use | Number | 2021 | 47.26181 | 75.28269 | 6.710988 |
| Deaths | Turkmenistan | Both | 55+ years | Liver cancer | Drug use | Number | 2021 | 46.69906 | 84.61219 | 4.243564 |
| Deaths | Slovak Republic | Both | 55+ years | Liver cancer | Drug use | Number | 2021 | 44.51598 | 85.51357 | 5.691524 |
| Deaths | Republic of South Africa | Both | 55+ years | Liver cancer | Drug use | Number | 2021 | 43.84587 | 72.80976 | 24.16217 |
| Deaths | Democratic Socialist Republic of Sri Lanka | Both | 55+ years | Liver cancer | Drug use | Number | 2021 | 43.30897 | 88.0099 | 3.626731 |
| Deaths | Republic of Guatemala | Both | 55+ years | Liver cancer | Drug use | Number | 2021 | 42.18501 | 94.20951 | 3.212844 |
| Deaths | Bosnia and Herzegovina | Both | 55+ years | Liver cancer | Drug use | Number | 2021 | 41.3369 | 82.01169 | 6.347766 |
| Deaths | Republic of Moldova | Both | 55+ years | Liver cancer | Drug use | Number | 2021 | 40.78431 | 63.73656 | 8.176792 |
| Deaths | Kingdom of Denmark | Both | 55+ years | Liver cancer | Drug use | Number | 2021 | 40.59854 | 79.41384 | 3.15777 |
| Deaths | Federal Republic of Nigeria | Both | 55+ years | Liver cancer | Drug use | Number | 2021 | 39.00822 | 67.60866 | 21.20124 |
| Deaths | North Macedonia | Both | 55+ years | Liver cancer | Drug use | Number | 2021 | 38.47885 | 68.97042 | 2.779448 |
| Deaths | Republic of Singapore | Both | 55+ years | Liver cancer | Drug use | Number | 2021 | 37.18815 | 73.74078 | 5.946404 |
| Deaths | Kingdom of Cambodia | Both | 55+ years | Liver cancer | Drug use | Number | 2021 | 37.11761 | 102.7915 | 1.85014 |
| Deaths | Republic of Peru | Both | 55+ years | Liver cancer | Drug use | Number | 2021 | 35.86437 | 77.07268 | 6.293102 |
| Deaths | Republic of Slovenia | Both | 55+ years | Liver cancer | Drug use | Number | 2021 | 34.84419 | 58.44401 | 3.788817 |
| Deaths | Republic of Costa Rica | Both | 55+ years | Liver cancer | Drug use | Number | 2021 | 33.6298 | 66.98019 | 4.396077 |
| Deaths | People's Republic of Bangladesh | Both | 55+ years | Liver cancer | Drug use | Number | 2021 | 32.6707 | 73.15777 | 0.977807 |
| Deaths | Lao People's Democratic Republic | Both | 55+ years | Liver cancer | Drug use | Number | 2021 | 31.83612 | 64.2127 | 4.497186 |
| Deaths | Republic of Mali | Both | 55+ years | Liver cancer | Drug use | Number | 2021 | 30.02687 | 71.73826 | 3.573786 |
| Deaths | Republic of Cuba | Both | 55+ years | Liver cancer | Drug use | Number | 2021 | 28.87427 | 55.95331 | 4.970479 |
| Deaths | Republic of Iraq | Both | 55+ years | Liver cancer | Drug use | Number | 2021 | 27.36714 | 62.09913 | 2.991143 |
| Deaths | Republic of Paraguay | Both | 55+ years | Liver cancer | Drug use | Number | 2021 | 26.89361 | 53.75146 | 3.770388 |
| Deaths | Republic of Estonia | Both | 55+ years | Liver cancer | Drug use | Number | 2021 | 25.30936 | 39.33531 | 6.065853 |
| Deaths | Republic of Ecuador | Both | 55+ years | Liver cancer | Drug use | Number | 2021 | 23.79912 | 48.84157 | 3.256248 |
| Deaths | Kingdom of Saudi Arabia | Both | 55+ years | Liver cancer | Drug use | Number | 2021 | 19.97929 | 48.90574 | 1.845943 |
| Deaths | Syrian Arab Republic | Both | 55+ years | Liver cancer | Drug use | Number | 2021 | 18.33218 | 42.32547 | 2.413251 |
| Deaths | Republic of Lithuania | Both | 55+ years | Liver cancer | Drug use | Number | 2021 | 17.82583 | 33.80198 | 2.383635 |
| Deaths | People's Democratic Republic of Algeria | Both | 55+ years | Liver cancer | Drug use | Number | 2021 | 17.62162 | 39.69873 | 2.254453 |
| Deaths | Republic of Honduras | Both | 55+ years | Liver cancer | Drug use | Number | 2021 | 17.59101 | 39.37621 | 1.125728 |
| Deaths | Democratic Republic of the Congo | Both | 55+ years | Liver cancer | Drug use | Number | 2021 | 17.49781 | 50.6394 | 0.747133 |
| Deaths | Independent State of Papua New Guinea | Both | 55+ years | Liver cancer | Drug use | Number | 2021 | 16.85467 | 45.40951 | 0.779209 |
| Deaths | Republic of Croatia | Both | 55+ years | Liver cancer | Drug use | Number | 2021 | 16.71879 | 30.84172 | 2.055489 |
| Deaths | Puerto Rico | Both | 55+ years | Liver cancer | Drug use | Number | 2021 | 16.49008 | 34.32793 | 2.342301 |
| Deaths | Republic of Latvia | Both | 55+ years | Liver cancer | Drug use | Number | 2021 | 15.68273 | 30.41046 | 2.238677 |
| Deaths | Republic of Panama | Both | 55+ years | Liver cancer | Drug use | Number | 2021 | 14.86597 | 29.33223 | 1.407411 |
| Deaths | Hungary | Both | 55+ years | Liver cancer | Drug use | Number | 2021 | 14.5552 | 32.73872 | 1.061667 |
| Deaths | Republic of Armenia | Both | 55+ years | Liver cancer | Drug use | Number | 2021 | 14.48422 | 30.43809 | 1.335028 |
| Deaths | Plurinational State of Bolivia | Both | 55+ years | Liver cancer | Drug use | Number | 2021 | 14.28493 | 31.73829 | 2.44529 |
| Deaths | United Arab Emirates | Both | 55+ years | Liver cancer | Drug use | Number | 2021 | 14.12592 | 35.0323 | 1.237058 |
| Deaths | Republic of Sudan | Both | 55+ years | Liver cancer | Drug use | Number | 2021 | 13.21178 | 33.27569 | 1.086265 |
| Deaths | Dominican Republic | Both | 55+ years | Liver cancer | Drug use | Number | 2021 | 13.01108 | 27.58176 | 2.146336 |
| Deaths | Republic of Angola | Both | 55+ years | Liver cancer | Drug use | Number | 2021 | 12.77405 | 41.87025 | 0.695348 |
| Deaths | Federal Democratic Republic of Ethiopia | Both | 55+ years | Liver cancer | Drug use | Number | 2021 | 12.44786 | 24.44244 | 5.633184 |
| Deaths | Republic of Zimbabwe | Both | 55+ years | Liver cancer | Drug use | Number | 2021 | 11.81513 | 27.38797 | 0.637875 |
| Deaths | Republic of Tunisia | Both | 55+ years | Liver cancer | Drug use | Number | 2021 | 11.57966 | 28.75627 | 1.037963 |
| Deaths | Burkina Faso | Both | 55+ years | Liver cancer | Drug use | Number | 2021 | 11.53397 | 30.58705 | 1.259108 |
| Deaths | Grand Duchy of Luxembourg | Both | 55+ years | Liver cancer | Drug use | Number | 2021 | 11.20478 | 17.2812 | 0.765156 |
| Deaths | Republic of Cameroon | Both | 55+ years | Liver cancer | Drug use | Number | 2021 | 10.9496 | 27.69095 | 1.568059 |
| Deaths | Republic of Nicaragua | Both | 55+ years | Liver cancer | Drug use | Number | 2021 | 10.66713 | 22.88602 | 0.789294 |
| Deaths | Kingdom of Morocco | Both | 55+ years | Liver cancer | Drug use | Number | 2021 | 10.58757 | 23.20162 | 1.034604 |
| Deaths | Republic of Ghana | Both | 55+ years | Liver cancer | Drug use | Number | 2021 | 9.721576 | 23.47598 | 1.107306 |
| Deaths | Republic of Guinea | Both | 55+ years | Liver cancer | Drug use | Number | 2021 | 9.045675 | 21.6121 | 1.443864 |
| Deaths | Montenegro | Both | 55+ years | Liver cancer | Drug use | Number | 2021 | 8.944456 | 15.81238 | 1.470975 |
| Deaths | Republic of El Salvador | Both | 55+ years | Liver cancer | Drug use | Number | 2021 | 8.507727 | 17.42416 | 1.43384 |
| Deaths | Eastern Republic of Uruguay | Both | 55+ years | Liver cancer | Drug use | Number | 2021 | 8.329692 | 19.05285 | 1.041124 |
| Deaths | State of Libya | Both | 55+ years | Liver cancer | Drug use | Number | 2021 | 7.504421 | 16.6487 | 0.696131 |
| Deaths | Republic of the Niger | Both | 55+ years | Liver cancer | Drug use | Number | 2021 | 7.118623 | 18.21834 | 0.777245 |
| Deaths | Republic of Kenya | Both | 55+ years | Liver cancer | Drug use | Number | 2021 | 6.571823 | 11.0822 | 3.591392 |
| Deaths | Republic of Malta | Both | 55+ years | Liver cancer | Drug use | Number | 2021 | 6.44814 | 10.59242 | 0.617329 |
| Deaths | Republic of Haiti | Both | 55+ years | Liver cancer | Drug use | Number | 2021 | 6.397218 | 16.98195 | 0.625083 |
| Deaths | Republic of Chad | Both | 55+ years | Liver cancer | Drug use | Number | 2021 | 6.346776 | 16.13201 | 1.060692 |
| Deaths | Republic of Yemen | Both | 55+ years | Liver cancer | Drug use | Number | 2021 | 6.008525 | 14.9494 | 0.762632 |
| Deaths | Republic of Senegal | Both | 55+ years | Liver cancer | Drug use | Number | 2021 | 5.719257 | 12.37048 | 0.804426 |
| Deaths | Republic of Fiji | Both | 55+ years | Liver cancer | Drug use | Number | 2021 | 5.661637 | 9.635447 | 0.824726 |
| Deaths | Republic of Benin | Both | 55+ years | Liver cancer | Drug use | Number | 2021 | 5.434151 | 12.93688 | 0.723038 |
| Deaths | Republic of Iceland | Both | 55+ years | Liver cancer | Drug use | Number | 2021 | 5.362393 | 8.364385 | 0.952547 |
| Deaths | Jamaica | Both | 55+ years | Liver cancer | Drug use | Number | 2021 | 5.133605 | 10.25361 | 0.873776 |
| Deaths | Federal Republic of Somalia | Both | 55+ years | Liver cancer | Drug use | Number | 2021 | 5.087036 | 14.0852 | 0.402781 |
| Deaths | Republic of Madagascar | Both | 55+ years | Liver cancer | Drug use | Number | 2021 | 4.740553 | 11.28914 | 0.551336 |
| Deaths | Republic of Malawi | Both | 55+ years | Liver cancer | Drug use | Number | 2021 | 3.814387 | 8.500645 | 0.364469 |
| Deaths | Republic of Trinidad and Tobago | Both | 55+ years | Liver cancer | Drug use | Number | 2021 | 3.757921 | 7.945757 | 0.546267 |
| Deaths | Islamic Republic of Afghanistan | Both | 55+ years | Liver cancer | Drug use | Number | 2021 | 3.686752 | 7.95717 | 0.537534 |
| Deaths | Hashemite Kingdom of Jordan | Both | 55+ years | Liver cancer | Drug use | Number | 2021 | 3.678592 | 8.971964 | 0.485045 |
| Deaths | Islamic Republic of Mauritania | Both | 55+ years | Liver cancer | Drug use | Number | 2021 | 3.675775 | 8.938449 | 0.361186 |
| Deaths | Republic of C么te d'Ivoire | Both | 55+ years | Liver cancer | Drug use | Number | 2021 | 3.618101 | 8.507754 | 0.308066 |
| Deaths | Republic of Cyprus | Both | 55+ years | Liver cancer | Drug use | Number | 2021 | 3.382355 | 7.40417 | 0.16848 |
| Deaths | Lebanese Republic | Both | 55+ years | Liver cancer | Drug use | Number | 2021 | 3.371158 | 7.723527 | 0.416098 |
| Deaths | Palestine | Both | 55+ years | Liver cancer | Drug use | Number | 2021 | 3.300367 | 7.311558 | 0.221984 |
| Deaths | Republic of Maldives | Both | 55+ years | Liver cancer | Drug use | Number | 2021 | 3.21279 | 4.939009 | 0.935926 |
| Deaths | Brunei Darussalam | Both | 55+ years | Liver cancer | Drug use | Number | 2021 | 3.19828 | 6.275201 | 0.382871 |
| Deaths | Republic of Sierra Leone | Both | 55+ years | Liver cancer | Drug use | Number | 2021 | 3.046457 | 7.086404 | 0.306977 |
| Deaths | Democratic Republic of Timor-Leste | Both | 55+ years | Liver cancer | Drug use | Number | 2021 | 2.973826 | 6.053589 | 0.355212 |
| Deaths | Republic of Zambia | Both | 55+ years | Liver cancer | Drug use | Number | 2021 | 2.959168 | 9.230063 | 0.26267 |
| Deaths | Republic of South Sudan | Both | 55+ years | Liver cancer | Drug use | Number | 2021 | 2.862583 | 6.730562 | 0.310038 |
| Deaths | Republic of Mauritius | Both | 55+ years | Liver cancer | Drug use | Number | 2021 | 2.827641 | 4.186918 | 0.59168 |
| Deaths | Republic of the Congo | Both | 55+ years | Liver cancer | Drug use | Number | 2021 | 2.75619 | 7.446745 | 0.20874 |
| Deaths | Togolese Republic | Both | 55+ years | Liver cancer | Drug use | Number | 2021 | 2.70534 | 6.734069 | 0.281236 |
| Deaths | State of Qatar | Both | 55+ years | Liver cancer | Drug use | Number | 2021 | 2.703818 | 6.081074 | 0.240881 |
| Deaths | Republic of the Gambia | Both | 55+ years | Liver cancer | Drug use | Number | 2021 | 2.6532 | 6.237735 | 0.443769 |
| Deaths | Central African Republic | Both | 55+ years | Liver cancer | Drug use | Number | 2021 | 2.565218 | 7.305729 | 0.245446 |
| Deaths | Republic of Liberia | Both | 55+ years | Liver cancer | Drug use | Number | 2021 | 2.44085 | 5.845272 | 0.194388 |
| Deaths | Republic of Rwanda | Both | 55+ years | Liver cancer | Drug use | Number | 2021 | 2.410964 | 5.703836 | 0.224137 |
| Deaths | Principality of Andorra | Both | 55+ years | Liver cancer | Drug use | Number | 2021 | 2.293975 | 4.494521 | 0.340633 |
| Deaths | Kingdom of Tonga | Both | 55+ years | Liver cancer | Drug use | Number | 2021 | 2.161419 | 3.809782 | 0.248227 |
| Deaths | Kingdom of Lesotho | Both | 55+ years | Liver cancer | Drug use | Number | 2021 | 2.001187 | 5.898224 | 0.142184 |
| Deaths | Sultanate of Oman | Both | 55+ years | Liver cancer | Drug use | Number | 2021 | 1.856972 | 4.270734 | 0.150916 |
| Deaths | Solomon Islands | Both | 55+ years | Liver cancer | Drug use | Number | 2021 | 1.817417 | 3.595097 | 0.123676 |
| Deaths | Kingdom of Bhutan | Both | 55+ years | Liver cancer | Drug use | Number | 2021 | 1.783368 | 4.41793 | 0.074078 |
| Deaths | Guam | Both | 55+ years | Liver cancer | Drug use | Number | 2021 | 1.473352 | 2.284161 | 0.205079 |
| Deaths | Principality of Monaco | Both | 55+ years | Liver cancer | Drug use | Number | 2021 | 1.436941 | 2.71607 | 0.083782 |
| Deaths | Republic of Suriname | Both | 55+ years | Liver cancer | Drug use | Number | 2021 | 1.410468 | 2.942423 | 0.193182 |
| Deaths | Gabonese Republic | Both | 55+ years | Liver cancer | Drug use | Number | 2021 | 1.293903 | 3.157287 | 0.085308 |
| Deaths | Republic of Burundi | Both | 55+ years | Liver cancer | Drug use | Number | 2021 | 1.199745 | 3.117006 | 0.147234 |
| Deaths | Kingdom of Eswatini | Both | 55+ years | Liver cancer | Drug use | Number | 2021 | 1.173651 | 3.557945 | 0.132644 |
| Deaths | Barbados | Both | 55+ years | Liver cancer | Drug use | Number | 2021 | 1.154566 | 2.397098 | 0.204266 |
| Deaths | Republic of Guinea-Bissau | Both | 55+ years | Liver cancer | Drug use | Number | 2021 | 1.119207 | 2.43621 | 0.130716 |
| Deaths | Commonwealth of the Bahamas | Both | 55+ years | Liver cancer | Drug use | Number | 2021 | 1.052819 | 2.170384 | 0.138537 |
| Deaths | Kingdom of Bahrain | Both | 55+ years | Liver cancer | Drug use | Number | 2021 | 1.038932 | 2.505589 | 0.069612 |
| Deaths | State of Eritrea | Both | 55+ years | Liver cancer | Drug use | Number | 2021 | 1.023358 | 2.506155 | 0.14669 |
| Deaths | State of Kuwait | Both | 55+ years | Liver cancer | Drug use | Number | 2021 | 1.004704 | 2.165706 | 0.095964 |
| Deaths | Republic of Botswana | Both | 55+ years | Liver cancer | Drug use | Number | 2021 | 0.99123 | 2.691216 | 0.126013 |
| Deaths | Republic of Vanuatu | Both | 55+ years | Liver cancer | Drug use | Number | 2021 | 0.96565 | 1.927119 | 0.088997 |
| Deaths | Independent State of Samoa | Both | 55+ years | Liver cancer | Drug use | Number | 2021 | 0.963489 | 1.732068 | 0.091344 |
| Deaths | Republic of Guyana | Both | 55+ years | Liver cancer | Drug use | Number | 2021 | 0.950824 | 2.134515 | 0.096858 |
| Deaths | Republic of Uganda | Both | 55+ years | Liver cancer | Drug use | Number | 2021 | 0.675227 | 1.716282 | 0.076612 |
| Deaths | Republic of Kiribati | Both | 55+ years | Liver cancer | Drug use | Number | 2021 | 0.635143 | 1.175813 | 0.067881 |
| Deaths | Republic of Namibia | Both | 55+ years | Liver cancer | Drug use | Number | 2021 | 0.553441 | 1.25396 | 0.057685 |
| Deaths | Belize | Both | 55+ years | Liver cancer | Drug use | Number | 2021 | 0.533922 | 1.06933 | 0.090032 |
| Deaths | Republic of Seychelles | Both | 55+ years | Liver cancer | Drug use | Number | 2021 | 0.521129 | 1.012543 | 0.054547 |
| Deaths | Republic of Cabo Verde | Both | 55+ years | Liver cancer | Drug use | Number | 2021 | 0.513851 | 1.190741 | 0.063132 |
| Deaths | Federated States of Micronesia | Both | 55+ years | Liver cancer | Drug use | Number | 2021 | 0.496268 | 0.928684 | 0.058314 |
| Deaths | Northern Mariana Islands | Both | 55+ years | Liver cancer | Drug use | Number | 2021 | 0.448541 | 0.734061 | 0.077495 |
| Deaths | United States Virgin Islands | Both | 55+ years | Liver cancer | Drug use | Number | 2021 | 0.428218 | 0.899457 | 0.044194 |
| Deaths | American Samoa | Both | 55+ years | Liver cancer | Drug use | Number | 2021 | 0.418989 | 0.703972 | 0.063584 |
| Deaths | Republic of Equatorial Guinea | Both | 55+ years | Liver cancer | Drug use | Number | 2021 | 0.379882 | 0.924259 | 0.033571 |
| Deaths | Cook Islands | Both | 55+ years | Liver cancer | Drug use | Number | 2021 | 0.368256 | 0.618081 | 0.056495 |
| Deaths | Republic of Djibouti | Both | 55+ years | Liver cancer | Drug use | Number | 2021 | 0.354004 | 0.899496 | 0.03485 |
| Deaths | Grenada | Both | 55+ years | Liver cancer | Drug use | Number | 2021 | 0.339297 | 0.697564 | 0.057279 |
| Deaths | Saint Vincent and the Grenadines | Both | 55+ years | Liver cancer | Drug use | Number | 2021 | 0.322707 | 0.641448 | 0.03865 |
| Deaths | Republic of San Marino | Both | 55+ years | Liver cancer | Drug use | Number | 2021 | 0.319318 | 0.616276 | 0.024707 |
| Deaths | Greenland | Both | 55+ years | Liver cancer | Drug use | Number | 2021 | 0.300834 | 0.605956 | 0.043486 |
| Deaths | Saint Lucia | Both | 55+ years | Liver cancer | Drug use | Number | 2021 | 0.281039 | 0.563029 | 0.038827 |
| Deaths | Union of the Comoros | Both | 55+ years | Liver cancer | Drug use | Number | 2021 | 0.275485 | 0.643595 | 0.024099 |
| Deaths | Commonwealth of Dominica | Both | 55+ years | Liver cancer | Drug use | Number | 2021 | 0.266133 | 0.565974 | 0.041542 |
| Deaths | Antigua and Barbuda | Both | 55+ years | Liver cancer | Drug use | Number | 2021 | 0.263272 | 0.559189 | 0.033564 |
| Deaths | Saint Kitts and Nevis | Both | 55+ years | Liver cancer | Drug use | Number | 2021 | 0.209636 | 0.443293 | 0.038486 |
| Deaths | Bermuda | Both | 55+ years | Liver cancer | Drug use | Number | 2021 | 0.189626 | 0.361048 | 0.031418 |
| Deaths | Republic of Palau | Both | 55+ years | Liver cancer | Drug use | Number | 2021 | 0.185453 | 0.309598 | 0.042459 |
| Deaths | Republic of the Marshall Islands | Both | 55+ years | Liver cancer | Drug use | Number | 2021 | 0.173107 | 0.36521 | 0.017495 |
| Deaths | Tuvalu | Both | 55+ years | Liver cancer | Drug use | Number | 2021 | 0.071251 | 0.13343 | 0.011158 |
| Deaths | Republic of Nauru | Both | 55+ years | Liver cancer | Drug use | Number | 2021 | 0.033224 | 0.060411 | 0.005091 |
| Deaths | Democratic Republic of Sao Tome and Principe | Both | 55+ years | Liver cancer | Drug use | Number | 2021 | 0.020015 | 0.05144 | 0.002198 |
| Deaths | Republic of Niue | Both | 55+ years | Liver cancer | Drug use | Number | 2021 | 0.016227 | 0.028505 | 0.003032 |
| Deaths | Tokelau | Both | 55+ years | Liver cancer | Drug use | Number | 2021 | 0.009966 | 0.020343 | 0.00073 |

| measure | location | sex | age | cause | rei | metric | year | val | upper | lower |
| --- | --- | --- | --- | --- | --- | --- | --- | --- | --- | --- |
| Deaths | Mongolia | Both | Age-standardized | Liver cancer | Drug use | Rate | 2021 | 12.19816 | 23.5521 | 0.733782 |
| Deaths | Kingdom of Tonga | Both | Age-standardized | Liver cancer | Drug use | Rate | 2021 | 2.720259 | 4.794809 | 0.312406 |
| Deaths | Kingdom of Spain | Both | Age-standardized | Liver cancer | Drug use | Rate | 2021 | 1.975441 | 2.87499 | 0.404468 |
| Deaths | Republic of Serbia | Both | Age-standardized | Liver cancer | Drug use | Rate | 2021 | 1.821008 | 2.8305 | 0.44922 |
| Deaths | French Republic | Both | Age-standardized | Liver cancer | Drug use | Rate | 2021 | 1.728576 | 2.682866 | 0.156922 |
| Deaths | Principality of Monaco | Both | Age-standardized | Liver cancer | Drug use | Rate | 2021 | 1.552449 | 2.9344 | 0.090517 |
| Deaths | Republic of Azerbaijan | Both | Age-standardized | Liver cancer | Drug use | Rate | 2021 | 1.470131 | 3.166836 | 0.129372 |
| Deaths | Principality of Andorra | Both | Age-standardized | Liver cancer | Drug use | Rate | 2021 | 1.465324 | 2.870969 | 0.217587 |
| Deaths | United States of America | Both | Age-standardized | Liver cancer | Drug use | Rate | 2021 | 1.458872 | 1.696178 | 1.218024 |
| Deaths | Japan | Both | Age-standardized | Liver cancer | Drug use | Rate | 2021 | 1.443059 | 2.048982 | 0.936785 |
| Deaths | United Kingdom of Great Britain and Northern Ireland | Both | Age-standardized | Liver cancer | Drug use | Rate | 2021 | 1.410139 | 1.758128 | 1.070749 |
| Deaths | Republic of Kazakhstan | Both | Age-standardized | Liver cancer | Drug use | Rate | 2021 | 1.342193 | 1.982306 | 0.207477 |
| Deaths | Cook Islands | Both | Age-standardized | Liver cancer | Drug use | Rate | 2021 | 1.320753 | 2.216754 | 0.202621 |
| Deaths | Republic of Italy | Both | Age-standardized | Liver cancer | Drug use | Rate | 2021 | 1.238608 | 1.696369 | 0.807253 |
| Deaths | Turkmenistan | Both | Age-standardized | Liver cancer | Drug use | Rate | 2021 | 1.115378 | 2.02091 | 0.101355 |
| Deaths | Swiss Confederation | Both | Age-standardized | Liver cancer | Drug use | Rate | 2021 | 1.115126 | 1.687763 | 0.207596 |
| Deaths | Republic of Uzbekistan | Both | Age-standardized | Liver cancer | Drug use | Rate | 2021 | 1.102889 | 1.829691 | 0.073264 |
| Deaths | North Macedonia | Both | Age-standardized | Liver cancer | Drug use | Rate | 2021 | 1.096984 | 1.96626 | 0.079239 |
| Deaths | Grand Duchy of Luxembourg | Both | Age-standardized | Liver cancer | Drug use | Rate | 2021 | 1.065797 | 1.643786 | 0.072782 |
| Deaths | Republic of Tajikistan | Both | Age-standardized | Liver cancer | Drug use | Rate | 2021 | 1.040396 | 2.066054 | 0.10083 |
| Deaths | Republic of Albania | Both | Age-standardized | Liver cancer | Drug use | Rate | 2021 | 1.035244 | 2.088398 | 0.096429 |
| Deaths | Kingdom of Norway | Both | Age-standardized | Liver cancer | Drug use | Rate | 2021 | 1.008787 | 1.300665 | 0.709435 |
| Deaths | Australia | Both | Age-standardized | Liver cancer | Drug use | Rate | 2021 | 0.998879 | 1.638253 | 0.040879 |
| Deaths | Republic of Maldives | Both | Age-standardized | Liver cancer | Drug use | Rate | 2021 | 0.998334 | 1.534735 | 0.290827 |
| Deaths | Republic of Estonia | Both | Age-standardized | Liver cancer | Drug use | Rate | 2021 | 0.977893 | 1.519822 | 0.23437 |
| Deaths | Kyrgyz Republic | Both | Age-standardized | Liver cancer | Drug use | Rate | 2021 | 0.944565 | 1.504585 | 0.134124 |
| Deaths | Republic of Mozambique | Both | Age-standardized | Liver cancer | Drug use | Rate | 2021 | 0.941365 | 2.610072 | 0.065649 |
| Deaths | Socialist Republic of Viet Nam | Both | Age-standardized | Liver cancer | Drug use | Rate | 2021 | 0.938316 | 1.849996 | 0.096575 |
| Deaths | Republic of Iceland | Both | Age-standardized | Liver cancer | Drug use | Rate | 2021 | 0.930733 | 1.451779 | 0.165331 |
| Deaths | Brunei Darussalam | Both | Age-standardized | Liver cancer | Drug use | Rate | 2021 | 0.902896 | 1.771532 | 0.108087 |
| Deaths | Montenegro | Both | Age-standardized | Liver cancer | Drug use | Rate | 2021 | 0.867038 | 1.532785 | 0.14259 |
| Deaths | Georgia | Both | Age-standardized | Liver cancer | Drug use | Rate | 2021 | 0.853947 | 1.382931 | 0.111663 |
| Deaths | Republic of Kiribati | Both | Age-standardized | Liver cancer | Drug use | Rate | 2021 | 0.853619 | 1.580266 | 0.09123 |
| Deaths | American Samoa | Both | Age-standardized | Liver cancer | Drug use | Rate | 2021 | 0.838503 | 1.408826 | 0.127248 |
| Deaths | Republic of Slovenia | Both | Age-standardized | Liver cancer | Drug use | Rate | 2021 | 0.804398 | 1.349214 | 0.087467 |
| Deaths | Taiwan (Province of China) | Both | Age-standardized | Liver cancer | Drug use | Rate | 2021 | 0.79402 | 1.729849 | 0.052123 |
| Deaths | Northern Mariana Islands | Both | Age-standardized | Liver cancer | Drug use | Rate | 2021 | 0.779981 | 1.276482 | 0.134758 |
| Deaths | Kingdom of Thailand | Both | Age-standardized | Liver cancer | Drug use | Rate | 2021 | 0.779922 | 1.432242 | 0.074535 |
| Deaths | People's Republic of China | Both | Age-standardized | Liver cancer | Drug use | Rate | 2021 | 0.7788 | 1.089465 | 0.490169 |
| Deaths | Republic of Palau | Both | Age-standardized | Liver cancer | Drug use | Rate | 2021 | 0.748615 | 1.24975 | 0.171393 |
| Deaths | Portuguese Republic | Both | Age-standardized | Liver cancer | Drug use | Rate | 2021 | 0.742985 | 1.325868 | 0.058538 |
| Deaths | Federal Republic of Germany | Both | Age-standardized | Liver cancer | Drug use | Rate | 2021 | 0.734954 | 1.263598 | 0.133771 |
| Deaths | Republic of Belarus | Both | Age-standardized | Liver cancer | Drug use | Rate | 2021 | 0.71295 | 1.080556 | 0.265951 |
| Deaths | New Zealand | Both | Age-standardized | Liver cancer | Drug use | Rate | 2021 | 0.71157 | 0.979493 | 0.256298 |
| Deaths | Republic of Niue | Both | Age-standardized | Liver cancer | Drug use | Rate | 2021 | 0.701033 | 1.231467 | 0.130989 |
| Deaths | Republic of Fiji | Both | Age-standardized | Liver cancer | Drug use | Rate | 2021 | 0.699855 | 1.191072 | 0.101947 |
| Deaths | Lao People's Democratic Republic | Both | Age-standardized | Liver cancer | Drug use | Rate | 2021 | 0.696018 | 1.403853 | 0.09832 |
| Deaths | Republic of Malta | Both | Age-standardized | Liver cancer | Drug use | Rate | 2021 | 0.690689 | 1.134601 | 0.066125 |
| Deaths | Tokelau | Both | Age-standardized | Liver cancer | Drug use | Rate | 2021 | 0.66648 | 1.360504 | 0.048799 |
| Deaths | Independent State of Samoa | Both | Age-standardized | Liver cancer | Drug use | Rate | 2021 | 0.661448 | 1.189087 | 0.062709 |
| Deaths | Tuvalu | Both | Age-standardized | Liver cancer | Drug use | Rate | 2021 | 0.660892 | 1.237641 | 0.1035 |
| Deaths | Guam | Both | Age-standardized | Liver cancer | Drug use | Rate | 2021 | 0.660841 | 1.024512 | 0.091984 |
| Deaths | Republic of Korea | Both | Age-standardized | Liver cancer | Drug use | Rate | 2021 | 0.659623 | 1.313906 | 0.111489 |
| Deaths | Republic of Moldova | Both | Age-standardized | Liver cancer | Drug use | Rate | 2021 | 0.652923 | 1.020369 | 0.130904 |
| Deaths | Bosnia and Herzegovina | Both | Age-standardized | Liver cancer | Drug use | Rate | 2021 | 0.637942 | 1.265666 | 0.097963 |
| Deaths | Federated States of Micronesia | Both | Age-standardized | Liver cancer | Drug use | Rate | 2021 | 0.637458 | 1.192899 | 0.074905 |
| Deaths | Ireland | Both | Age-standardized | Liver cancer | Drug use | Rate | 2021 | 0.626877 | 1.064129 | 0.080204 |
| Deaths | Republic of Nauru | Both | Age-standardized | Liver cancer | Drug use | Rate | 2021 | 0.60759 | 1.104782 | 0.0931 |
| Deaths | Kingdom of Belgium | Both | Age-standardized | Liver cancer | Drug use | Rate | 2021 | 0.597713 | 1.124329 | 0.038147 |
| Deaths | Republic of Costa Rica | Both | Age-standardized | Liver cancer | Drug use | Rate | 2021 | 0.593699 | 1.182465 | 0.077608 |
| Deaths | State of Israel | Both | Age-standardized | Liver cancer | Drug use | Rate | 2021 | 0.576099 | 0.972731 | 0.054204 |
| Deaths | Republic of the Philippines | Both | Age-standardized | Liver cancer | Drug use | Rate | 2021 | 0.571071 | 0.802259 | 0.375566 |
| Deaths | Republic of Vanuatu | Both | Age-standardized | Liver cancer | Drug use | Rate | 2021 | 0.569226 | 1.135987 | 0.052461 |
| Deaths | Solomon Islands | Both | Age-standardized | Liver cancer | Drug use | Rate | 2021 | 0.566308 | 1.120234 | 0.038537 |
| Deaths | Democratic People's Republic of Korea | Both | Age-standardized | Liver cancer | Drug use | Rate | 2021 | 0.554994 | 1.12765 | 0.036062 |
| Deaths | Russian Federation | Both | Age-standardized | Liver cancer | Drug use | Rate | 2021 | 0.52458 | 0.654543 | 0.385253 |
| Deaths | Republic of Chile | Both | Age-standardized | Liver cancer | Drug use | Rate | 2021 | 0.517497 | 0.986405 | 0.03686 |
| Deaths | Republic of the Marshall Islands | Both | Age-standardized | Liver cancer | Drug use | Rate | 2021 | 0.499407 | 1.05362 | 0.050473 |
| Deaths | Islamic Republic of Pakistan | Both | Age-standardized | Liver cancer | Drug use | Rate | 2021 | 0.493139 | 0.959067 | 0.166523 |
| Deaths | Republic of the Union of Myanmar | Both | Age-standardized | Liver cancer | Drug use | Rate | 2021 | 0.466263 | 1.086132 | 0.025393 |
| Deaths | Republic of Paraguay | Both | Age-standardized | Liver cancer | Drug use | Rate | 2021 | 0.459126 | 0.917641 | 0.064368 |
| Deaths | Slovak Republic | Both | Age-standardized | Liver cancer | Drug use | Rate | 2021 | 0.458736 | 0.881215 | 0.058651 |
| Deaths | Republic of San Marino | Both | Age-standardized | Liver cancer | Drug use | Rate | 2021 | 0.456366 | 0.880774 | 0.035311 |
| Deaths | Republic of Finland | Both | Age-standardized | Liver cancer | Drug use | Rate | 2021 | 0.445289 | 0.922619 | 0.050927 |
| Deaths | Malaysia | Both | Age-standardized | Liver cancer | Drug use | Rate | 2021 | 0.424999 | 0.798009 | 0.040685 |
| Deaths | Republic of Seychelles | Both | Age-standardized | Liver cancer | Drug use | Rate | 2021 | 0.424658 | 0.825101 | 0.044449 |
| Deaths | Republic of Singapore | Both | Age-standardized | Liver cancer | Drug use | Rate | 2021 | 0.413866 | 0.82066 | 0.066177 |
| Deaths | Republic of Latvia | Both | Age-standardized | Liver cancer | Drug use | Rate | 2021 | 0.402853 | 0.781175 | 0.057506 |
| Deaths | Republic of Guatemala | Both | Age-standardized | Liver cancer | Drug use | Rate | 2021 | 0.389482 | 0.869809 | 0.029663 |
| Deaths | Republic of Austria | Both | Age-standardized | Liver cancer | Drug use | Rate | 2021 | 0.388948 | 0.750508 | 0.046575 |
| Deaths | Republic of Bulgaria | Both | Age-standardized | Liver cancer | Drug use | Rate | 2021 | 0.388918 | 0.733386 | 0.063033 |
| Deaths | Greenland | Both | Age-standardized | Liver cancer | Drug use | Rate | 2021 | 0.374558 | 0.754455 | 0.054143 |
| Deaths | United Mexican States | Both | Age-standardized | Liver cancer | Drug use | Rate | 2021 | 0.359453 | 0.546814 | 0.212369 |
| Deaths | Kingdom of Denmark | Both | Age-standardized | Liver cancer | Drug use | Rate | 2021 | 0.356446 | 0.697235 | 0.027724 |
| Deaths | Democratic Republic of Timor-Leste | Both | Age-standardized | Liver cancer | Drug use | Rate | 2021 | 0.353913 | 0.720433 | 0.042274 |
| Deaths | Republic of Mali | Both | Age-standardized | Liver cancer | Drug use | Rate | 2021 | 0.350666 | 0.837788 | 0.041736 |
| Deaths | Independent State of Papua New Guinea | Both | Age-standardized | Liver cancer | Drug use | Rate | 2021 | 0.346881 | 0.93456 | 0.016037 |
| Deaths | Republic of Panama | Both | Age-standardized | Liver cancer | Drug use | Rate | 2021 | 0.337939 | 0.666792 | 0.031994 |
| Deaths | United Arab Emirates | Both | Age-standardized | Liver cancer | Drug use | Rate | 2021 | 0.332513 | 0.824632 | 0.029119 |
| Deaths | Kingdom of Sweden | Both | Age-standardized | Liver cancer | Drug use | Rate | 2021 | 0.31788 | 0.576631 | 0.10386 |
| Deaths | Republic of India | Both | Age-standardized | Liver cancer | Drug use | Rate | 2021 | 0.314745 | 0.41892 | 0.225021 |
| Deaths | Federal Democratic Republic of Nepal | Both | Age-standardized | Liver cancer | Drug use | Rate | 2021 | 0.313507 | 0.678123 | 0.039468 |
| Deaths | Kingdom of the Netherlands | Both | Age-standardized | Liver cancer | Drug use | Rate | 2021 | 0.313028 | 0.565773 | 0.031181 |
| Deaths | Republic of Lithuania | Both | Age-standardized | Liver cancer | Drug use | Rate | 2021 | 0.311341 | 0.590377 | 0.041632 |
| Deaths | Republic of Armenia | Both | Age-standardized | Liver cancer | Drug use | Rate | 2021 | 0.311239 | 0.654059 | 0.028687 |
| Deaths | Kingdom of Bhutan | Both | Age-standardized | Liver cancer | Drug use | Rate | 2021 | 0.302683 | 0.749836 | 0.012573 |
| Deaths | State of Qatar | Both | Age-standardized | Liver cancer | Drug use | Rate | 2021 | 0.298351 | 0.671012 | 0.02658 |
| Deaths | Commonwealth of Dominica | Both | Age-standardized | Liver cancer | Drug use | Rate | 2021 | 0.29825 | 0.634276 | 0.046556 |
| Deaths | Hellenic Republic | Both | Age-standardized | Liver cancer | Drug use | Rate | 2021 | 0.291273 | 0.562962 | 0.040038 |
| Deaths | Republic of the Gambia | Both | Age-standardized | Liver cancer | Drug use | Rate | 2021 | 0.290009 | 0.681818 | 0.048506 |
| Deaths | Kingdom of Cambodia | Both | Age-standardized | Liver cancer | Drug use | Rate | 2021 | 0.288617 | 0.79928 | 0.014386 |
| Deaths | Federative Republic of Brazil | Both | Age-standardized | Liver cancer | Drug use | Rate | 2021 | 0.286134 | 0.421176 | 0.167324 |
| Deaths | Grenada | Both | Age-standardized | Liver cancer | Drug use | Rate | 2021 | 0.285513 | 0.586989 | 0.048199 |
| Deaths | Czech Republic | Both | Age-standardized | Liver cancer | Drug use | Rate | 2021 | 0.284004 | 0.511381 | 0.045291 |
| Deaths | Republic of Honduras | Both | Age-standardized | Liver cancer | Drug use | Rate | 2021 | 0.282732 | 0.632876 | 0.018093 |
| Deaths | United Republic of Tanzania | Both | Age-standardized | Liver cancer | Drug use | Rate | 2021 | 0.281596 | 0.617854 | 0.031165 |
| Deaths | Saint Kitts and Nevis | Both | Age-standardized | Liver cancer | Drug use | Rate | 2021 | 0.275129 | 0.581781 | 0.050509 |
| Deaths | Romania | Both | Age-standardized | Liver cancer | Drug use | Rate | 2021 | 0.264025 | 0.536064 | 0.016198 |
| Deaths | Bolivarian Republic of Venezuela | Both | Age-standardized | Liver cancer | Drug use | Rate | 2021 | 0.248983 | 0.515148 | 0.014409 |
| Deaths | Islamic Republic of Iran | Both | Age-standardized | Liver cancer | Drug use | Rate | 2021 | 0.24868 | 0.339266 | 0.170706 |
| Deaths | Commonwealth of the Bahamas | Both | Age-standardized | Liver cancer | Drug use | Rate | 2021 | 0.247306 | 0.509821 | 0.032542 |
| Deaths | Arab Republic of Egypt | Both | Age-standardized | Liver cancer | Drug use | Rate | 2021 | 0.243266 | 0.538639 | 0.016144 |
| Deaths | Puerto Rico | Both | Age-standardized | Liver cancer | Drug use | Rate | 2021 | 0.23877 | 0.497056 | 0.033916 |
| Deaths | Republic of Poland | Both | Age-standardized | Liver cancer | Drug use | Rate | 2021 | 0.236452 | 0.310235 | 0.165083 |
| Deaths | Antigua and Barbuda | Both | Age-standardized | Liver cancer | Drug use | Rate | 2021 | 0.235858 | 0.500962 | 0.030069 |
| Deaths | United States Virgin Islands | Both | Age-standardized | Liver cancer | Drug use | Rate | 2021 | 0.224863 | 0.472317 | 0.023207 |
| Deaths | Republic of Nicaragua | Both | Age-standardized | Liver cancer | Drug use | Rate | 2021 | 0.221498 | 0.475217 | 0.016389 |
| Deaths | Ukraine | Both | Age-standardized | Liver cancer | Drug use | Rate | 2021 | 0.220829 | 0.372786 | 0.070845 |
| Deaths | Kingdom of Eswatini | Both | Age-standardized | Liver cancer | Drug use | Rate | 2021 | 0.219111 | 0.664241 | 0.024764 |
| Deaths | Saint Vincent and the Grenadines | Both | Age-standardized | Liver cancer | Drug use | Rate | 2021 | 0.214536 | 0.426435 | 0.025695 |
| Deaths | Barbados | Both | Age-standardized | Liver cancer | Drug use | Rate | 2021 | 0.214178 | 0.444674 | 0.037892 |
| Deaths | Republic of Suriname | Both | Age-standardized | Liver cancer | Drug use | Rate | 2021 | 0.213434 | 0.445251 | 0.029233 |
| Deaths | Republic of Croatia | Both | Age-standardized | Liver cancer | Drug use | Rate | 2021 | 0.189494 | 0.349566 | 0.023297 |
| Deaths | Kingdom of Lesotho | Both | Age-standardized | Liver cancer | Drug use | Rate | 2021 | 0.187346 | 0.552175 | 0.013311 |
| Deaths | Republic of Indonesia | Both | Age-standardized | Liver cancer | Drug use | Rate | 2021 | 0.18479 | 0.355706 | 0.081206 |
| Deaths | Republic of Trinidad and Tobago | Both | Age-standardized | Liver cancer | Drug use | Rate | 2021 | 0.182352 | 0.385565 | 0.026507 |
| Deaths | Republic of Zimbabwe | Both | Age-standardized | Liver cancer | Drug use | Rate | 2021 | 0.180363 | 0.41809 | 0.009737 |
| Deaths | Belize | Both | Age-standardized | Liver cancer | Drug use | Rate | 2021 | 0.180186 | 0.360874 | 0.030384 |
| Deaths | Islamic Republic of Mauritania | Both | Age-standardized | Liver cancer | Drug use | Rate | 2021 | 0.173185 | 0.421138 | 0.017017 |
| Deaths | Republic of Guinea | Both | Age-standardized | Liver cancer | Drug use | Rate | 2021 | 0.167367 | 0.399875 | 0.026715 |
| Deaths | Republic of Guinea-Bissau | Both | Age-standardized | Liver cancer | Drug use | Rate | 2021 | 0.16469 | 0.358485 | 0.019235 |
| Deaths | Jamaica | Both | Age-standardized | Liver cancer | Drug use | Rate | 2021 | 0.164066 | 0.327698 | 0.027925 |
| Deaths | Republic of Cyprus | Both | Age-standardized | Liver cancer | Drug use | Rate | 2021 | 0.163581 | 0.358089 | 0.008148 |
| Deaths | Plurinational State of Bolivia | Both | Age-standardized | Liver cancer | Drug use | Rate | 2021 | 0.156236 | 0.347125 | 0.026744 |
| Deaths | Eastern Republic of Uruguay | Both | Age-standardized | Liver cancer | Drug use | Rate | 2021 | 0.155698 | 0.356135 | 0.019461 |
| Deaths | Republic of Colombia | Both | Age-standardized | Liver cancer | Drug use | Rate | 2021 | 0.155546 | 0.320762 | 0.012463 |
| Deaths | Democratic Socialist Republic of Sri Lanka | Both | Age-standardized | Liver cancer | Drug use | Rate | 2021 | 0.151994 | 0.308874 | 0.012728 |
| Deaths | State of Libya | Both | Age-standardized | Liver cancer | Drug use | Rate | 2021 | 0.151674 | 0.336491 | 0.01407 |
| Deaths | Republic of Ecuador | Both | Age-standardized | Liver cancer | Drug use | Rate | 2021 | 0.145356 | 0.298307 | 0.019888 |
| Deaths | Republic of Cuba | Both | Age-standardized | Liver cancer | Drug use | Rate | 2021 | 0.142831 | 0.276783 | 0.024587 |
| Deaths | Republic of Guyana | Both | Age-standardized | Liver cancer | Drug use | Rate | 2021 | 0.142711 | 0.320374 | 0.014538 |
| Deaths | Republic of Mauritius | Both | Age-standardized | Liver cancer | Drug use | Rate | 2021 | 0.141268 | 0.209178 | 0.02956 |
| Deaths | Republic of El Salvador | Both | Age-standardized | Liver cancer | Drug use | Rate | 2021 | 0.14063 | 0.288016 | 0.023701 |
| Deaths | Bermuda | Both | Age-standardized | Liver cancer | Drug use | Rate | 2021 | 0.137618 | 0.262025 | 0.022801 |
| Deaths | Dominican Republic | Both | Age-standardized | Liver cancer | Drug use | Rate | 2021 | 0.131612 | 0.278999 | 0.021711 |
| Deaths | Syrian Arab Republic | Both | Age-standardized | Liver cancer | Drug use | Rate | 2021 | 0.13115 | 0.3028 | 0.017265 |
| Deaths | Canada | Both | Age-standardized | Liver cancer | Drug use | Rate | 2021 | 0.130006 | 0.265096 | 0.018651 |
| Deaths | Burkina Faso | Both | Age-standardized | Liver cancer | Drug use | Rate | 2021 | 0.129677 | 0.343893 | 0.014156 |
| Deaths | Palestine | Both | Age-standardized | Liver cancer | Drug use | Rate | 2021 | 0.129657 | 0.287238 | 0.008721 |
| Deaths | Republic of Liberia | Both | Age-standardized | Liver cancer | Drug use | Rate | 2021 | 0.124977 | 0.299292 | 0.009953 |
| Deaths | Gabonese Republic | Both | Age-standardized | Liver cancer | Drug use | Rate | 2021 | 0.122353 | 0.298557 | 0.008067 |
| Deaths | Central African Republic | Both | Age-standardized | Liver cancer | Drug use | Rate | 2021 | 0.121219 | 0.345231 | 0.011599 |
| Deaths | Argentine Republic | Both | Age-standardized | Liver cancer | Drug use | Rate | 2021 | 0.119753 | 0.241343 | 0.010213 |
| Deaths | Republic of Iraq | Both | Age-standardized | Liver cancer | Drug use | Rate | 2021 | 0.119064 | 0.27017 | 0.013013 |
| Deaths | Republic of Chad | Both | Age-standardized | Liver cancer | Drug use | Rate | 2021 | 0.115538 | 0.29367 | 0.019309 |
| Deaths | Republic of Cabo Verde | Both | Age-standardized | Liver cancer | Drug use | Rate | 2021 | 0.113321 | 0.262597 | 0.013923 |
| Deaths | Saint Lucia | Both | Age-standardized | Liver cancer | Drug use | Rate | 2021 | 0.112885 | 0.226152 | 0.015595 |
| Deaths | Kingdom of Saudi Arabia | Both | Age-standardized | Liver cancer | Drug use | Rate | 2021 | 0.11217 | 0.274572 | 0.010364 |
| Deaths | Republic of Angola | Both | Age-standardized | Liver cancer | Drug use | Rate | 2021 | 0.111666 | 0.366012 | 0.006078 |
| Deaths | Republic of Benin | Both | Age-standardized | Liver cancer | Drug use | Rate | 2021 | 0.11106 | 0.264396 | 0.014777 |
| Deaths | Republic of Peru | Both | Age-standardized | Liver cancer | Drug use | Rate | 2021 | 0.10838 | 0.232908 | 0.019017 |
| Deaths | Kingdom of Bahrain | Both | Age-standardized | Liver cancer | Drug use | Rate | 2021 | 0.108153 | 0.260832 | 0.007247 |
| Deaths | Republic of the Congo | Both | Age-standardized | Liver cancer | Drug use | Rate | 2021 | 0.107215 | 0.289676 | 0.00812 |
| Deaths | Sultanate of Oman | Both | Age-standardized | Liver cancer | Drug use | Rate | 2021 | 0.10049 | 0.23111 | 0.008167 |
| Deaths | Republic of South Africa | Both | Age-standardized | Liver cancer | Drug use | Rate | 2021 | 0.09395 | 0.156011 | 0.051773 |
| Deaths | Republic of Haiti | Both | Age-standardized | Liver cancer | Drug use | Rate | 2021 | 0.091244 | 0.242215 | 0.008916 |
| Deaths | Federal Republic of Somalia | Both | Age-standardized | Liver cancer | Drug use | Rate | 2021 | 0.091178 | 0.252457 | 0.007219 |
| Deaths | Republic of Cameroon | Both | Age-standardized | Liver cancer | Drug use | Rate | 2021 | 0.090928 | 0.229951 | 0.013021 |
| Deaths | Republic of the Niger | Both | Age-standardized | Liver cancer | Drug use | Rate | 2021 | 0.086428 | 0.22119 | 0.009437 |
| Deaths | Republic of Sierra Leone | Both | Age-standardized | Liver cancer | Drug use | Rate | 2021 | 0.085244 | 0.198286 | 0.00859 |
| Deaths | Republic of Tunisia | Both | Age-standardized | Liver cancer | Drug use | Rate | 2021 | 0.083748 | 0.207976 | 0.007507 |
| Deaths | Republic of Equatorial Guinea | Both | Age-standardized | Liver cancer | Drug use | Rate | 2021 | 0.081181 | 0.197514 | 0.007174 |
| Deaths | Republic of South Sudan | Both | Age-standardized | Liver cancer | Drug use | Rate | 2021 | 0.077701 | 0.182692 | 0.008416 |
| Deaths | Hungary | Both | Age-standardized | Liver cancer | Drug use | Rate | 2021 | 0.077364 | 0.174013 | 0.005643 |
| Deaths | Republic of Senegal | Both | Age-standardized | Liver cancer | Drug use | Rate | 2021 | 0.074872 | 0.161945 | 0.010531 |
| Deaths | Republic of Turkey | Both | Age-standardized | Liver cancer | Drug use | Rate | 2021 | 0.073319 | 0.171613 | 0.007346 |
| Deaths | Togolese Republic | Both | Age-standardized | Liver cancer | Drug use | Rate | 2021 | 0.073202 | 0.182213 | 0.00761 |
| Deaths | Republic of Sudan | Both | Age-standardized | Liver cancer | Drug use | Rate | 2021 | 0.071718 | 0.180632 | 0.005897 |
| Deaths | Republic of Botswana | Both | Age-standardized | Liver cancer | Drug use | Rate | 2021 | 0.069114 | 0.187645 | 0.008786 |
| Deaths | Republic of Ghana | Both | Age-standardized | Liver cancer | Drug use | Rate | 2021 | 0.060254 | 0.145504 | 0.006863 |
| Deaths | Lebanese Republic | Both | Age-standardized | Liver cancer | Drug use | Rate | 2021 | 0.058144 | 0.133211 | 0.007177 |
| Deaths | Republic of Djibouti | Both | Age-standardized | Liver cancer | Drug use | Rate | 2021 | 0.0577 | 0.146612 | 0.00568 |
| Deaths | Union of the Comoros | Both | Age-standardized | Liver cancer | Drug use | Rate | 2021 | 0.057608 | 0.134586 | 0.005039 |
| Deaths | Republic of Malawi | Both | Age-standardized | Liver cancer | Drug use | Rate | 2021 | 0.055148 | 0.122901 | 0.005269 |
| Deaths | Islamic Republic of Afghanistan | Both | Age-standardized | Liver cancer | Drug use | Rate | 2021 | 0.050716 | 0.109461 | 0.007394 |
| Deaths | Hashemite Kingdom of Jordan | Both | Age-standardized | Liver cancer | Drug use | Rate | 2021 | 0.049457 | 0.120624 | 0.006521 |
| Deaths | People's Democratic Republic of Algeria | Both | Age-standardized | Liver cancer | Drug use | Rate | 2021 | 0.049043 | 0.110487 | 0.006274 |
| Deaths | Democratic Republic of the Congo | Both | Age-standardized | Liver cancer | Drug use | Rate | 2021 | 0.048969 | 0.141719 | 0.002091 |
| Deaths | Republic of Zambia | Both | Age-standardized | Liver cancer | Drug use | Rate | 2021 | 0.046307 | 0.144438 | 0.00411 |
| Deaths | Republic of Yemen | Both | Age-standardized | Liver cancer | Drug use | Rate | 2021 | 0.04516 | 0.11236 | 0.005732 |
| Deaths | Federal Republic of Nigeria | Both | Age-standardized | Liver cancer | Drug use | Rate | 2021 | 0.043953 | 0.076178 | 0.023889 |
| Deaths | Republic of Madagascar | Both | Age-standardized | Liver cancer | Drug use | Rate | 2021 | 0.043391 | 0.103332 | 0.005046 |
| Deaths | Republic of Namibia | Both | Age-standardized | Liver cancer | Drug use | Rate | 2021 | 0.041403 | 0.093808 | 0.004315 |
| Deaths | State of Eritrea | Both | Age-standardized | Liver cancer | Drug use | Rate | 2021 | 0.039259 | 0.096143 | 0.005627 |
| Deaths | Republic of Rwanda | Both | Age-standardized | Liver cancer | Drug use | Rate | 2021 | 0.038538 | 0.091173 | 0.003583 |
| Deaths | State of Kuwait | Both | Age-standardized | Liver cancer | Drug use | Rate | 2021 | 0.036426 | 0.078518 | 0.003479 |
| Deaths | Republic of C么te d'Ivoire | Both | Age-standardized | Liver cancer | Drug use | Rate | 2021 | 0.034105 | 0.080196 | 0.002904 |
| Deaths | Federal Democratic Republic of Ethiopia | Both | Age-standardized | Liver cancer | Drug use | Rate | 2021 | 0.030736 | 0.060353 | 0.013909 |
| Deaths | Kingdom of Morocco | Both | Age-standardized | Liver cancer | Drug use | Rate | 2021 | 0.029787 | 0.065276 | 0.002911 |
| Deaths | Republic of Kenya | Both | Age-standardized | Liver cancer | Drug use | Rate | 2021 | 0.029695 | 0.050076 | 0.016228 |
| Deaths | Republic of Burundi | Both | Age-standardized | Liver cancer | Drug use | Rate | 2021 | 0.025332 | 0.065815 | 0.003109 |
| Deaths | People's Republic of Bangladesh | Both | Age-standardized | Liver cancer | Drug use | Rate | 2021 | 0.023634 | 0.052922 | 0.000707 |
| Deaths | Democratic Republic of Sao Tome and Principe | Both | Age-standardized | Liver cancer | Drug use | Rate | 2021 | 0.018504 | 0.047555 | 0.002032 |
| Deaths | Republic of Uganda | Both | Age-standardized | Liver cancer | Drug use | Rate | 2021 | 0.004813 | 0.012233 | 0.000546 |

| measure | location | sex | age | cause | rei | metric | year | val | upper | lower |
| --- | --- | --- | --- | --- | --- | --- | --- | --- | --- | --- |
| DALYs (Disability-Adjusted Life Years) | People's Republic of China | Both | 55+ years | Liver cancer | Drug use | Number | 2021 | 383708.8 | 533510.4 | 246147.3 |
| DALYs (Disability-Adjusted Life Years) | United States of America | Both | 55+ years | Liver cancer | Drug use | Number | 2021 | 186618.1 | 218054.2 | 156865.6 |
| DALYs (Disability-Adjusted Life Years) | Republic of India | Both | 55+ years | Liver cancer | Drug use | Number | 2021 | 89569.54 | 119720.7 | 64317.91 |
| DALYs (Disability-Adjusted Life Years) | Japan | Both | 55+ years | Liver cancer | Drug use | Number | 2021 | 88252.02 | 122472.5 | 57870.89 |
| DALYs (Disability-Adjusted Life Years) | French Republic | Both | 55+ years | Liver cancer | Drug use | Number | 2021 | 45975.16 | 69600.76 | 4756.456 |
| DALYs (Disability-Adjusted Life Years) | Kingdom of Spain | Both | 55+ years | Liver cancer | Drug use | Number | 2021 | 36172.33 | 50993.99 | 8348.205 |
| DALYs (Disability-Adjusted Life Years) | Republic of Italy | Both | 55+ years | Liver cancer | Drug use | Number | 2021 | 33724.07 | 44825.14 | 23054.72 |
| DALYs (Disability-Adjusted Life Years) | United Kingdom of Great Britain and Northern Ireland | Both | 55+ years | Liver cancer | Drug use | Number | 2021 | 33025.64 | 40773.43 | 25463.64 |
| DALYs (Disability-Adjusted Life Years) | Russian Federation | Both | 55+ years | Liver cancer | Drug use | Number | 2021 | 29091.23 | 36433.27 | 22002.75 |
| DALYs (Disability-Adjusted Life Years) | Federal Republic of Germany | Both | 55+ years | Liver cancer | Drug use | Number | 2021 | 28013.84 | 46379.64 | 5775.576 |
| DALYs (Disability-Adjusted Life Years) | Socialist Republic of Viet Nam | Both | 55+ years | Liver cancer | Drug use | Number | 2021 | 24464.93 | 48151.37 | 2639.504 |
| DALYs (Disability-Adjusted Life Years) | Kingdom of Thailand | Both | 55+ years | Liver cancer | Drug use | Number | 2021 | 19515.41 | 35321.09 | 2167.97 |
| DALYs (Disability-Adjusted Life Years) | Federative Republic of Brazil | Both | 55+ years | Liver cancer | Drug use | Number | 2021 | 17073.83 | 25064.68 | 10045.08 |
| DALYs (Disability-Adjusted Life Years) | Republic of Korea | Both | 55+ years | Liver cancer | Drug use | Number | 2021 | 15602.19 | 30804.82 | 2698.596 |
| DALYs (Disability-Adjusted Life Years) | Islamic Republic of Pakistan | Both | 55+ years | Liver cancer | Drug use | Number | 2021 | 14996.79 | 29840.38 | 4891.098 |
| DALYs (Disability-Adjusted Life Years) | Republic of Indonesia | Both | 55+ years | Liver cancer | Drug use | Number | 2021 | 11422.6 | 22031.74 | 4999.209 |
| DALYs (Disability-Adjusted Life Years) | Republic of the Philippines | Both | 55+ years | Liver cancer | Drug use | Number | 2021 | 11368.81 | 15861.46 | 7519.873 |
| DALYs (Disability-Adjusted Life Years) | United Mexican States | Both | 55+ years | Liver cancer | Drug use | Number | 2021 | 10717.48 | 16056.1 | 6346.853 |
| DALYs (Disability-Adjusted Life Years) | Australia | Both | 55+ years | Liver cancer | Drug use | Number | 2021 | 9383.783 | 14944.39 | 421.1216 |
| DALYs (Disability-Adjusted Life Years) | Taiwan (Province of China) | Both | 55+ years | Liver cancer | Drug use | Number | 2021 | 8150.899 | 17489.73 | 575.7574 |
| DALYs (Disability-Adjusted Life Years) | Republic of Uzbekistan | Both | 55+ years | Liver cancer | Drug use | Number | 2021 | 7756.634 | 12850.76 | 541.3899 |
| DALYs (Disability-Adjusted Life Years) | Mongolia | Both | 55+ years | Liver cancer | Drug use | Number | 2021 | 7297.393 | 14011.81 | 482.9939 |
| DALYs (Disability-Adjusted Life Years) | Republic of Serbia | Both | 55+ years | Liver cancer | Drug use | Number | 2021 | 6247.004 | 9750.412 | 1767.43 |
| DALYs (Disability-Adjusted Life Years) | Republic of Kazakhstan | Both | 55+ years | Liver cancer | Drug use | Number | 2021 | 5892.388 | 8834.442 | 1027.06 |
| DALYs (Disability-Adjusted Life Years) | Republic of the Union of Myanmar | Both | 55+ years | Liver cancer | Drug use | Number | 2021 | 5612.481 | 13177.41 | 329.059 |
| DALYs (Disability-Adjusted Life Years) | Democratic People's Republic of Korea | Both | 55+ years | Liver cancer | Drug use | Number | 2021 | 4450.657 | 8891.638 | 308.263 |
| DALYs (Disability-Adjusted Life Years) | Republic of Azerbaijan | Both | 55+ years | Liver cancer | Drug use | Number | 2021 | 4221.334 | 9094.351 | 402.3087 |
| DALYs (Disability-Adjusted Life Years) | Arab Republic of Egypt | Both | 55+ years | Liver cancer | Drug use | Number | 2021 | 4105.404 | 9162.279 | 298.4471 |
| DALYs (Disability-Adjusted Life Years) | Ukraine | Both | 55+ years | Liver cancer | Drug use | Number | 2021 | 4096.395 | 6936.89 | 1350.62 |
| DALYs (Disability-Adjusted Life Years) | Islamic Republic of Iran | Both | 55+ years | Liver cancer | Drug use | Number | 2021 | 3906.74 | 5431.181 | 2672.458 |
| DALYs (Disability-Adjusted Life Years) | Portuguese Republic | Both | 55+ years | Liver cancer | Drug use | Number | 2021 | 3732.283 | 6563.283 | 315.9148 |
| DALYs (Disability-Adjusted Life Years) | Swiss Confederation | Both | 55+ years | Liver cancer | Drug use | Number | 2021 | 3689.71 | 5533.817 | 800.6425 |
| DALYs (Disability-Adjusted Life Years) | Republic of Poland | Both | 55+ years | Liver cancer | Drug use | Number | 2021 | 3637.426 | 4780.899 | 2580.585 |
| DALYs (Disability-Adjusted Life Years) | Republic of Chile | Both | 55+ years | Liver cancer | Drug use | Number | 2021 | 3160.927 | 5911.256 | 223.4333 |
| DALYs (Disability-Adjusted Life Years) | Malaysia | Both | 55+ years | Liver cancer | Drug use | Number | 2021 | 2952.307 | 5585.93 | 296.3464 |
| DALYs (Disability-Adjusted Life Years) | Kingdom of Belgium | Both | 55+ years | Liver cancer | Drug use | Number | 2021 | 2896.417 | 5336.212 | 188.8928 |
| DALYs (Disability-Adjusted Life Years) | Republic of Belarus | Both | 55+ years | Liver cancer | Drug use | Number | 2021 | 2723.361 | 4233.083 | 1139.824 |
| DALYs (Disability-Adjusted Life Years) | Canada | Both | 55+ years | Liver cancer | Drug use | Number | 2021 | 2557.489 | 5125.104 | 360.2166 |
| DALYs (Disability-Adjusted Life Years) | Republic of Mozambique | Both | 55+ years | Liver cancer | Drug use | Number | 2021 | 2422.58 | 6730.422 | 166.248 |
| DALYs (Disability-Adjusted Life Years) | Kingdom of the Netherlands | Both | 55+ years | Liver cancer | Drug use | Number | 2021 | 2331.247 | 4113.854 | 246.0775 |
| DALYs (Disability-Adjusted Life Years) | Romania | Both | 55+ years | Liver cancer | Drug use | Number | 2021 | 2143.768 | 4296.191 | 143.4959 |
| DALYs (Disability-Adjusted Life Years) | Kingdom of Norway | Both | 55+ years | Liver cancer | Drug use | Number | 2021 | 1909.718 | 2423.071 | 1402.746 |
| DALYs (Disability-Adjusted Life Years) | Republic of Colombia | Both | 55+ years | Liver cancer | Drug use | Number | 2021 | 1870.171 | 3846.219 | 156.09 |
| DALYs (Disability-Adjusted Life Years) | Federal Democratic Republic of Nepal | Both | 55+ years | Liver cancer | Drug use | Number | 2021 | 1864.579 | 3907.839 | 244.5256 |
| DALYs (Disability-Adjusted Life Years) | Bolivarian Republic of Venezuela | Both | 55+ years | Liver cancer | Drug use | Number | 2021 | 1776.148 | 3586.473 | 115.1483 |
| DALYs (Disability-Adjusted Life Years) | Republic of Turkey | Both | 55+ years | Liver cancer | Drug use | Number | 2021 | 1694.778 | 4018.361 | 183.6098 |
| DALYs (Disability-Adjusted Life Years) | United Republic of Tanzania | Both | 55+ years | Liver cancer | Drug use | Number | 2021 | 1682.632 | 3744.94 | 196.0793 |
| DALYs (Disability-Adjusted Life Years) | Argentine Republic | Both | 55+ years | Liver cancer | Drug use | Number | 2021 | 1593.319 | 3156.83 | 140.4587 |
| DALYs (Disability-Adjusted Life Years) | Republic of Tajikistan | Both | 55+ years | Liver cancer | Drug use | Number | 2021 | 1577.928 | 3101.481 | 158.2514 |
| DALYs (Disability-Adjusted Life Years) | Republic of Austria | Both | 55+ years | Liver cancer | Drug use | Number | 2021 | 1538.732 | 2977.289 | 196.0059 |
| DALYs (Disability-Adjusted Life Years) | State of Israel | Both | 55+ years | Liver cancer | Drug use | Number | 2021 | 1420.371 | 2316.296 | 141.771 |
| DALYs (Disability-Adjusted Life Years) | Kingdom of Sweden | Both | 55+ years | Liver cancer | Drug use | Number | 2021 | 1307.36 | 2291.026 | 431.3283 |
| DALYs (Disability-Adjusted Life Years) | Hellenic Republic | Both | 55+ years | Liver cancer | Drug use | Number | 2021 | 1290.249 | 2448.504 | 193.0983 |
| DALYs (Disability-Adjusted Life Years) | New Zealand | Both | 55+ years | Liver cancer | Drug use | Number | 2021 | 1288.655 | 1721.232 | 536.0482 |
| DALYs (Disability-Adjusted Life Years) | Czech Republic | Both | 55+ years | Liver cancer | Drug use | Number | 2021 | 1243.398 | 2237.446 | 205.9224 |
| DALYs (Disability-Adjusted Life Years) | Georgia | Both | 55+ years | Liver cancer | Drug use | Number | 2021 | 1240.961 | 1992.263 | 174.5367 |
| DALYs (Disability-Adjusted Life Years) | Turkmenistan | Both | 55+ years | Liver cancer | Drug use | Number | 2021 | 1219.072 | 2245.976 | 117.3958 |
| DALYs (Disability-Adjusted Life Years) | Republic of Bulgaria | Both | 55+ years | Liver cancer | Drug use | Number | 2021 | 1198.064 | 2235.841 | 212.4408 |
| DALYs (Disability-Adjusted Life Years) | Kyrgyz Republic | Both | 55+ years | Liver cancer | Drug use | Number | 2021 | 1177.66 | 1889.655 | 180.1861 |
| DALYs (Disability-Adjusted Life Years) | Republic of Finland | Both | 55+ years | Liver cancer | Drug use | Number | 2021 | 1114.081 | 2242.476 | 129.7468 |
| DALYs (Disability-Adjusted Life Years) | Republic of South Africa | Both | 55+ years | Liver cancer | Drug use | Number | 2021 | 1055.094 | 1755.048 | 580.7907 |
| DALYs (Disability-Adjusted Life Years) | Republic of Albania | Both | 55+ years | Liver cancer | Drug use | Number | 2021 | 1042.433 | 2008.656 | 103.7768 |
| DALYs (Disability-Adjusted Life Years) | Ireland | Both | 55+ years | Liver cancer | Drug use | Number | 2021 | 1018.858 | 1664.906 | 141.2884 |
| DALYs (Disability-Adjusted Life Years) | Slovak Republic | Both | 55+ years | Liver cancer | Drug use | Number | 2021 | 994.5365 | 1839.596 | 135.7174 |
| DALYs (Disability-Adjusted Life Years) | Democratic Socialist Republic of Sri Lanka | Both | 55+ years | Liver cancer | Drug use | Number | 2021 | 986.4656 | 2012.882 | 91.67163 |
| DALYs (Disability-Adjusted Life Years) | Republic of Guatemala | Both | 55+ years | Liver cancer | Drug use | Number | 2021 | 957.85 | 2178.836 | 77.84762 |
| DALYs (Disability-Adjusted Life Years) | Bosnia and Herzegovina | Both | 55+ years | Liver cancer | Drug use | Number | 2021 | 934.8835 | 1782.357 | 151 |
| DALYs (Disability-Adjusted Life Years) | Federal Republic of Nigeria | Both | 55+ years | Liver cancer | Drug use | Number | 2021 | 923.4668 | 1621.926 | 498.7757 |
| DALYs (Disability-Adjusted Life Years) | Kingdom of Cambodia | Both | 55+ years | Liver cancer | Drug use | Number | 2021 | 913.7934 | 2492.281 | 49.52666 |
| DALYs (Disability-Adjusted Life Years) | Republic of Moldova | Both | 55+ years | Liver cancer | Drug use | Number | 2021 | 911.0429 | 1448.507 | 208.0015 |
| DALYs (Disability-Adjusted Life Years) | Kingdom of Denmark | Both | 55+ years | Liver cancer | Drug use | Number | 2021 | 886.1038 | 1660.997 | 74.27298 |
| DALYs (Disability-Adjusted Life Years) | Republic of Singapore | Both | 55+ years | Liver cancer | Drug use | Number | 2021 | 878.4416 | 1725.5 | 145.1576 |
| DALYs (Disability-Adjusted Life Years) | North Macedonia | Both | 55+ years | Liver cancer | Drug use | Number | 2021 | 857.4843 | 1491.228 | 64.82857 |
| DALYs (Disability-Adjusted Life Years) | People's Republic of Bangladesh | Both | 55+ years | Liver cancer | Drug use | Number | 2021 | 850.2147 | 1946.066 | 25.76389 |
| DALYs (Disability-Adjusted Life Years) | Republic of Peru | Both | 55+ years | Liver cancer | Drug use | Number | 2021 | 790.5897 | 1687.208 | 133.363 |
| DALYs (Disability-Adjusted Life Years) | Lao People's Democratic Republic | Both | 55+ years | Liver cancer | Drug use | Number | 2021 | 774.0862 | 1539.565 | 115.1568 |
| DALYs (Disability-Adjusted Life Years) | Republic of Mali | Both | 55+ years | Liver cancer | Drug use | Number | 2021 | 754.0289 | 1814.195 | 91.26668 |
| DALYs (Disability-Adjusted Life Years) | Republic of Costa Rica | Both | 55+ years | Liver cancer | Drug use | Number | 2021 | 750.7815 | 1447.597 | 101.5682 |
| DALYs (Disability-Adjusted Life Years) | Republic of Slovenia | Both | 55+ years | Liver cancer | Drug use | Number | 2021 | 697.2315 | 1120.6 | 87.18425 |
| DALYs (Disability-Adjusted Life Years) | Republic of Iraq | Both | 55+ years | Liver cancer | Drug use | Number | 2021 | 678.6951 | 1547.225 | 76.61926 |
| DALYs (Disability-Adjusted Life Years) | Republic of Cuba | Both | 55+ years | Liver cancer | Drug use | Number | 2021 | 660.5955 | 1261.304 | 112.9933 |
| DALYs (Disability-Adjusted Life Years) | Republic of Paraguay | Both | 55+ years | Liver cancer | Drug use | Number | 2021 | 631.4168 | 1219.718 | 96.37848 |
| DALYs (Disability-Adjusted Life Years) | Kingdom of Saudi Arabia | Both | 55+ years | Liver cancer | Drug use | Number | 2021 | 519.8375 | 1264.051 | 47.40954 |
| DALYs (Disability-Adjusted Life Years) | Republic of Ecuador | Both | 55+ years | Liver cancer | Drug use | Number | 2021 | 519.5648 | 1060.605 | 73.23455 |
| DALYs (Disability-Adjusted Life Years) | Republic of Estonia | Both | 55+ years | Liver cancer | Drug use | Number | 2021 | 497.5187 | 765.5088 | 138.4415 |
| DALYs (Disability-Adjusted Life Years) | Syrian Arab Republic | Both | 55+ years | Liver cancer | Drug use | Number | 2021 | 460.2884 | 1074.195 | 63.1698 |
| DALYs (Disability-Adjusted Life Years) | Democratic Republic of the Congo | Both | 55+ years | Liver cancer | Drug use | Number | 2021 | 455.4633 | 1320.423 | 20.06329 |
| DALYs (Disability-Adjusted Life Years) | People's Democratic Republic of Algeria | Both | 55+ years | Liver cancer | Drug use | Number | 2021 | 426.4932 | 972.8513 | 54.9085 |
| DALYs (Disability-Adjusted Life Years) | Independent State of Papua New Guinea | Both | 55+ years | Liver cancer | Drug use | Number | 2021 | 419.5057 | 1123.111 | 22.10605 |
| DALYs (Disability-Adjusted Life Years) | Republic of Lithuania | Both | 55+ years | Liver cancer | Drug use | Number | 2021 | 412.6434 | 803.8203 | 64.65705 |
| DALYs (Disability-Adjusted Life Years) | Republic of Honduras | Both | 55+ years | Liver cancer | Drug use | Number | 2021 | 410.1982 | 896.119 | 27.50202 |
| DALYs (Disability-Adjusted Life Years) | United Arab Emirates | Both | 55+ years | Liver cancer | Drug use | Number | 2021 | 402.3681 | 1007.146 | 35.89559 |
| DALYs (Disability-Adjusted Life Years) | Puerto Rico | Both | 55+ years | Liver cancer | Drug use | Number | 2021 | 359.1713 | 743.6537 | 53.7024 |
| DALYs (Disability-Adjusted Life Years) | Republic of Croatia | Both | 55+ years | Liver cancer | Drug use | Number | 2021 | 350.5239 | 638.7963 | 47.52495 |
| DALYs (Disability-Adjusted Life Years) | Republic of Armenia | Both | 55+ years | Liver cancer | Drug use | Number | 2021 | 348.1938 | 732.7888 | 33.76114 |
| DALYs (Disability-Adjusted Life Years) | Republic of Latvia | Both | 55+ years | Liver cancer | Drug use | Number | 2021 | 342.1286 | 640.3747 | 50.08094 |
| DALYs (Disability-Adjusted Life Years) | Republic of Angola | Both | 55+ years | Liver cancer | Drug use | Number | 2021 | 337.5684 | 1114.384 | 19.39165 |
| DALYs (Disability-Adjusted Life Years) | Hungary | Both | 55+ years | Liver cancer | Drug use | Number | 2021 | 335.1761 | 765.4022 | 24.85405 |
| DALYs (Disability-Adjusted Life Years) | Plurinational State of Bolivia | Both | 55+ years | Liver cancer | Drug use | Number | 2021 | 331.0606 | 731.4177 | 58.85896 |
| DALYs (Disability-Adjusted Life Years) | Republic of Panama | Both | 55+ years | Liver cancer | Drug use | Number | 2021 | 324.6324 | 623.919 | 32.15445 |
| DALYs (Disability-Adjusted Life Years) | Republic of Sudan | Both | 55+ years | Liver cancer | Drug use | Number | 2021 | 321.3722 | 818.3618 | 26.23311 |
| DALYs (Disability-Adjusted Life Years) | Dominican Republic | Both | 55+ years | Liver cancer | Drug use | Number | 2021 | 310.268 | 653.0803 | 49.96369 |
| DALYs (Disability-Adjusted Life Years) | Federal Democratic Republic of Ethiopia | Both | 55+ years | Liver cancer | Drug use | Number | 2021 | 298.0269 | 586.7869 | 133.9118 |
| DALYs (Disability-Adjusted Life Years) | Republic of Zimbabwe | Both | 55+ years | Liver cancer | Drug use | Number | 2021 | 288.042 | 656.1555 | 16.11728 |
| DALYs (Disability-Adjusted Life Years) | Republic of Tunisia | Both | 55+ years | Liver cancer | Drug use | Number | 2021 | 285.1156 | 723.75 | 25.76987 |
| DALYs (Disability-Adjusted Life Years) | Burkina Faso | Both | 55+ years | Liver cancer | Drug use | Number | 2021 | 276.9469 | 714.0914 | 32.20776 |
| DALYs (Disability-Adjusted Life Years) | Kingdom of Morocco | Both | 55+ years | Liver cancer | Drug use | Number | 2021 | 275.5552 | 609.1374 | 28.07588 |
| DALYs (Disability-Adjusted Life Years) | Republic of Cameroon | Both | 55+ years | Liver cancer | Drug use | Number | 2021 | 265.8721 | 688.9058 | 38.14591 |
| DALYs (Disability-Adjusted Life Years) | Republic of Nicaragua | Both | 55+ years | Liver cancer | Drug use | Number | 2021 | 254.5079 | 557.3433 | 20.51362 |
| DALYs (Disability-Adjusted Life Years) | Republic of Ghana | Both | 55+ years | Liver cancer | Drug use | Number | 2021 | 223.9145 | 535.4548 | 25.92967 |
| DALYs (Disability-Adjusted Life Years) | Grand Duchy of Luxembourg | Both | 55+ years | Liver cancer | Drug use | Number | 2021 | 220.684 | 331.5539 | 17.75866 |
| DALYs (Disability-Adjusted Life Years) | Republic of Guinea | Both | 55+ years | Liver cancer | Drug use | Number | 2021 | 217.8216 | 533.0744 | 35.55724 |
| DALYs (Disability-Adjusted Life Years) | Eastern Republic of Uruguay | Both | 55+ years | Liver cancer | Drug use | Number | 2021 | 205.3611 | 472.9138 | 25.15487 |
| DALYs (Disability-Adjusted Life Years) | Montenegro | Both | 55+ years | Liver cancer | Drug use | Number | 2021 | 198.7161 | 350.2411 | 34.39434 |
| DALYs (Disability-Adjusted Life Years) | Republic of El Salvador | Both | 55+ years | Liver cancer | Drug use | Number | 2021 | 192.9129 | 392.2711 | 32.52366 |
| DALYs (Disability-Adjusted Life Years) | State of Libya | Both | 55+ years | Liver cancer | Drug use | Number | 2021 | 189.6691 | 425.7253 | 17.58881 |
| DALYs (Disability-Adjusted Life Years) | Republic of the Niger | Both | 55+ years | Liver cancer | Drug use | Number | 2021 | 172.8474 | 443.5709 | 18.48472 |
| DALYs (Disability-Adjusted Life Years) | Republic of Kenya | Both | 55+ years | Liver cancer | Drug use | Number | 2021 | 164.8697 | 280.829 | 89.09381 |
| DALYs (Disability-Adjusted Life Years) | Republic of Haiti | Both | 55+ years | Liver cancer | Drug use | Number | 2021 | 158.5474 | 432.2837 | 15.41208 |
| DALYs (Disability-Adjusted Life Years) | Republic of Chad | Both | 55+ years | Liver cancer | Drug use | Number | 2021 | 152.7803 | 388.8693 | 25.91343 |
| DALYs (Disability-Adjusted Life Years) | Republic of Yemen | Both | 55+ years | Liver cancer | Drug use | Number | 2021 | 149.3977 | 372.7509 | 20.16635 |
| DALYs (Disability-Adjusted Life Years) | Republic of Fiji | Both | 55+ years | Liver cancer | Drug use | Number | 2021 | 138.5388 | 234.5318 | 22.64598 |
| DALYs (Disability-Adjusted Life Years) | Republic of Senegal | Both | 55+ years | Liver cancer | Drug use | Number | 2021 | 135.0231 | 303.5171 | 19.27146 |
| DALYs (Disability-Adjusted Life Years) | Republic of Malta | Both | 55+ years | Liver cancer | Drug use | Number | 2021 | 132.5729 | 209.9815 | 13.06636 |
| DALYs (Disability-Adjusted Life Years) | Federal Republic of Somalia | Both | 55+ years | Liver cancer | Drug use | Number | 2021 | 131.1109 | 373.4944 | 10.36901 |
| DALYs (Disability-Adjusted Life Years) | Republic of Benin | Both | 55+ years | Liver cancer | Drug use | Number | 2021 | 128.3962 | 309.3913 | 17.07441 |
| DALYs (Disability-Adjusted Life Years) | Republic of Madagascar | Both | 55+ years | Liver cancer | Drug use | Number | 2021 | 126.667 | 303.9426 | 14.92912 |
| DALYs (Disability-Adjusted Life Years) | Jamaica | Both | 55+ years | Liver cancer | Drug use | Number | 2021 | 117.9045 | 232.9839 | 21.95807 |
| DALYs (Disability-Adjusted Life Years) | Republic of Iceland | Both | 55+ years | Liver cancer | Drug use | Number | 2021 | 109.3745 | 168.827 | 21.46418 |
| DALYs (Disability-Adjusted Life Years) | Republic of Malawi | Both | 55+ years | Liver cancer | Drug use | Number | 2021 | 96.94564 | 224.4282 | 9.252422 |
| DALYs (Disability-Adjusted Life Years) | Hashemite Kingdom of Jordan | Both | 55+ years | Liver cancer | Drug use | Number | 2021 | 93.20912 | 228.474 | 12.90372 |
| DALYs (Disability-Adjusted Life Years) | Republic of Trinidad and Tobago | Both | 55+ years | Liver cancer | Drug use | Number | 2021 | 90.70248 | 187.1745 | 13.18459 |
| DALYs (Disability-Adjusted Life Years) | Republic of C么te d'Ivoire | Both | 55+ years | Liver cancer | Drug use | Number | 2021 | 90.54509 | 215.553 | 7.805479 |
| DALYs (Disability-Adjusted Life Years) | Islamic Republic of Afghanistan | Both | 55+ years | Liver cancer | Drug use | Number | 2021 | 87.99693 | 197.1162 | 12.88038 |
| DALYs (Disability-Adjusted Life Years) | Islamic Republic of Mauritania | Both | 55+ years | Liver cancer | Drug use | Number | 2021 | 84.17295 | 210.2837 | 8.814661 |
| DALYs (Disability-Adjusted Life Years) | Brunei Darussalam | Both | 55+ years | Liver cancer | Drug use | Number | 2021 | 83.96485 | 164.703 | 10.15365 |
| DALYs (Disability-Adjusted Life Years) | Palestine | Both | 55+ years | Liver cancer | Drug use | Number | 2021 | 82.78437 | 191.261 | 5.825711 |
| DALYs (Disability-Adjusted Life Years) | Lebanese Republic | Both | 55+ years | Liver cancer | Drug use | Number | 2021 | 80.61368 | 185.4762 | 10.69863 |
| DALYs (Disability-Adjusted Life Years) | Republic of Cyprus | Both | 55+ years | Liver cancer | Drug use | Number | 2021 | 76.05371 | 163.4758 | 3.891492 |
| DALYs (Disability-Adjusted Life Years) | Republic of South Sudan | Both | 55+ years | Liver cancer | Drug use | Number | 2021 | 73.84227 | 179.587 | 8.390645 |
| DALYs (Disability-Adjusted Life Years) | Republic of Zambia | Both | 55+ years | Liver cancer | Drug use | Number | 2021 | 72.17963 | 227.8943 | 6.428067 |
| DALYs (Disability-Adjusted Life Years) | State of Qatar | Both | 55+ years | Liver cancer | Drug use | Number | 2021 | 72.09318 | 165.1006 | 6.660795 |
| DALYs (Disability-Adjusted Life Years) | Republic of the Congo | Both | 55+ years | Liver cancer | Drug use | Number | 2021 | 72.03409 | 195.0354 | 5.280409 |
| DALYs (Disability-Adjusted Life Years) | Republic of Sierra Leone | Both | 55+ years | Liver cancer | Drug use | Number | 2021 | 71.83956 | 168.5448 | 7.467739 |
| DALYs (Disability-Adjusted Life Years) | Central African Republic | Both | 55+ years | Liver cancer | Drug use | Number | 2021 | 70.46583 | 202.0149 | 7.01593 |
| DALYs (Disability-Adjusted Life Years) | Democratic Republic of Timor-Leste | Both | 55+ years | Liver cancer | Drug use | Number | 2021 | 68.95191 | 137.9535 | 8.73805 |
| DALYs (Disability-Adjusted Life Years) | Togolese Republic | Both | 55+ years | Liver cancer | Drug use | Number | 2021 | 66.12449 | 165.1979 | 7.095026 |
| DALYs (Disability-Adjusted Life Years) | Republic of the Gambia | Both | 55+ years | Liver cancer | Drug use | Number | 2021 | 65.41102 | 156.5845 | 11.0356 |
| DALYs (Disability-Adjusted Life Years) | Republic of Maldives | Both | 55+ years | Liver cancer | Drug use | Number | 2021 | 64.64074 | 102.3809 | 22.11344 |
| DALYs (Disability-Adjusted Life Years) | Republic of Mauritius | Both | 55+ years | Liver cancer | Drug use | Number | 2021 | 63.19177 | 94.34107 | 15.30829 |
| DALYs (Disability-Adjusted Life Years) | Republic of Rwanda | Both | 55+ years | Liver cancer | Drug use | Number | 2021 | 60.70975 | 142.372 | 5.448412 |
| DALYs (Disability-Adjusted Life Years) | Republic of Liberia | Both | 55+ years | Liver cancer | Drug use | Number | 2021 | 58.68019 | 139.3323 | 4.831263 |
| DALYs (Disability-Adjusted Life Years) | Principality of Andorra | Both | 55+ years | Liver cancer | Drug use | Number | 2021 | 51.27499 | 100.2307 | 7.808027 |
| DALYs (Disability-Adjusted Life Years) | Kingdom of Lesotho | Both | 55+ years | Liver cancer | Drug use | Number | 2021 | 50.71827 | 153.1682 | 3.647004 |
| DALYs (Disability-Adjusted Life Years) | Kingdom of Tonga | Both | 55+ years | Liver cancer | Drug use | Number | 2021 | 49.69321 | 86.17663 | 6.623445 |
| DALYs (Disability-Adjusted Life Years) | Sultanate of Oman | Both | 55+ years | Liver cancer | Drug use | Number | 2021 | 48.90771 | 113.9745 | 4.174404 |
| DALYs (Disability-Adjusted Life Years) | Solomon Islands | Both | 55+ years | Liver cancer | Drug use | Number | 2021 | 45.79626 | 94.19884 | 3.511389 |
| DALYs (Disability-Adjusted Life Years) | Kingdom of Bhutan | Both | 55+ years | Liver cancer | Drug use | Number | 2021 | 44.37946 | 108.3113 | 1.912914 |
| DALYs (Disability-Adjusted Life Years) | Republic of Suriname | Both | 55+ years | Liver cancer | Drug use | Number | 2021 | 35.01617 | 72.53349 | 4.977618 |
| DALYs (Disability-Adjusted Life Years) | Guam | Both | 55+ years | Liver cancer | Drug use | Number | 2021 | 34.23225 | 53.32969 | 5.429177 |
| DALYs (Disability-Adjusted Life Years) | Gabonese Republic | Both | 55+ years | Liver cancer | Drug use | Number | 2021 | 33.3257 | 84.30557 | 2.231034 |
| DALYs (Disability-Adjusted Life Years) | Republic of Burundi | Both | 55+ years | Liver cancer | Drug use | Number | 2021 | 30.81027 | 79.5715 | 3.746208 |
| DALYs (Disability-Adjusted Life Years) | Kingdom of Eswatini | Both | 55+ years | Liver cancer | Drug use | Number | 2021 | 29.61654 | 91.0156 | 3.222912 |
| DALYs (Disability-Adjusted Life Years) | Principality of Monaco | Both | 55+ years | Liver cancer | Drug use | Number | 2021 | 29.32361 | 53.90455 | 1.869318 |
| DALYs (Disability-Adjusted Life Years) | Republic of Guinea-Bissau | Both | 55+ years | Liver cancer | Drug use | Number | 2021 | 28.15595 | 62.90699 | 3.405043 |
| DALYs (Disability-Adjusted Life Years) | Kingdom of Bahrain | Both | 55+ years | Liver cancer | Drug use | Number | 2021 | 27.2385 | 67.44973 | 1.843985 |
| DALYs (Disability-Adjusted Life Years) | State of Eritrea | Both | 55+ years | Liver cancer | Drug use | Number | 2021 | 26.73114 | 66.64284 | 3.747301 |
| DALYs (Disability-Adjusted Life Years) | Commonwealth of the Bahamas | Both | 55+ years | Liver cancer | Drug use | Number | 2021 | 25.92211 | 53.8767 | 3.557802 |
| DALYs (Disability-Adjusted Life Years) | Barbados | Both | 55+ years | Liver cancer | Drug use | Number | 2021 | 25.41029 | 52.61992 | 4.667205 |
| DALYs (Disability-Adjusted Life Years) | Republic of Botswana | Both | 55+ years | Liver cancer | Drug use | Number | 2021 | 24.44496 | 68.11361 | 3.270871 |
| DALYs (Disability-Adjusted Life Years) | State of Kuwait | Both | 55+ years | Liver cancer | Drug use | Number | 2021 | 24.28907 | 52.92484 | 2.478674 |
| DALYs (Disability-Adjusted Life Years) | Republic of Guyana | Both | 55+ years | Liver cancer | Drug use | Number | 2021 | 24.24521 | 55.32793 | 2.6648 |
| DALYs (Disability-Adjusted Life Years) | Republic of Vanuatu | Both | 55+ years | Liver cancer | Drug use | Number | 2021 | 22.95542 | 45.53526 | 2.498922 |
| DALYs (Disability-Adjusted Life Years) | Independent State of Samoa | Both | 55+ years | Liver cancer | Drug use | Number | 2021 | 22.38351 | 39.97635 | 2.444413 |
| DALYs (Disability-Adjusted Life Years) | Republic of Uganda | Both | 55+ years | Liver cancer | Drug use | Number | 2021 | 16.4363 | 42.6333 | 1.873788 |
| DALYs (Disability-Adjusted Life Years) | Republic of Kiribati | Both | 55+ years | Liver cancer | Drug use | Number | 2021 | 16.26545 | 31.1032 | 1.75339 |
| DALYs (Disability-Adjusted Life Years) | Republic of Namibia | Both | 55+ years | Liver cancer | Drug use | Number | 2021 | 13.57647 | 32.12258 | 1.436724 |
| DALYs (Disability-Adjusted Life Years) | Belize | Both | 55+ years | Liver cancer | Drug use | Number | 2021 | 13.12588 | 26.34829 | 2.281125 |
| DALYs (Disability-Adjusted Life Years) | Federated States of Micronesia | Both | 55+ years | Liver cancer | Drug use | Number | 2021 | 12.81214 | 24.71857 | 1.632564 |
| DALYs (Disability-Adjusted Life Years) | Republic of Seychelles | Both | 55+ years | Liver cancer | Drug use | Number | 2021 | 12.28843 | 24.17847 | 1.323757 |
| DALYs (Disability-Adjusted Life Years) | Northern Mariana Islands | Both | 55+ years | Liver cancer | Drug use | Number | 2021 | 11.41664 | 18.78058 | 2.067704 |
| DALYs (Disability-Adjusted Life Years) | Republic of Cabo Verde | Both | 55+ years | Liver cancer | Drug use | Number | 2021 | 10.99808 | 26.65019 | 1.549715 |
| DALYs (Disability-Adjusted Life Years) | American Samoa | Both | 55+ years | Liver cancer | Drug use | Number | 2021 | 9.844659 | 16.64516 | 1.705757 |
| DALYs (Disability-Adjusted Life Years) | United States Virgin Islands | Both | 55+ years | Liver cancer | Drug use | Number | 2021 | 9.754062 | 19.90625 | 1.121327 |
| DALYs (Disability-Adjusted Life Years) | Republic of Equatorial Guinea | Both | 55+ years | Liver cancer | Drug use | Number | 2021 | 9.588737 | 23.41868 | 0.857773 |
| DALYs (Disability-Adjusted Life Years) | Republic of Djibouti | Both | 55+ years | Liver cancer | Drug use | Number | 2021 | 9.169309 | 23.3405 | 0.902142 |
| DALYs (Disability-Adjusted Life Years) | Greenland | Both | 55+ years | Liver cancer | Drug use | Number | 2021 | 8.528361 | 16.98672 | 1.243083 |
| DALYs (Disability-Adjusted Life Years) | Cook Islands | Both | 55+ years | Liver cancer | Drug use | Number | 2021 | 8.117582 | 13.76502 | 1.480557 |
| DALYs (Disability-Adjusted Life Years) | Grenada | Both | 55+ years | Liver cancer | Drug use | Number | 2021 | 7.876798 | 15.91345 | 1.340642 |
| DALYs (Disability-Adjusted Life Years) | Saint Vincent and the Grenadines | Both | 55+ years | Liver cancer | Drug use | Number | 2021 | 7.569731 | 14.55903 | 0.939661 |
| DALYs (Disability-Adjusted Life Years) | Saint Lucia | Both | 55+ years | Liver cancer | Drug use | Number | 2021 | 6.689174 | 13.37092 | 0.995485 |
| DALYs (Disability-Adjusted Life Years) | Union of the Comoros | Both | 55+ years | Liver cancer | Drug use | Number | 2021 | 6.650669 | 16.0017 | 0.574738 |
| DALYs (Disability-Adjusted Life Years) | Republic of San Marino | Both | 55+ years | Liver cancer | Drug use | Number | 2021 | 6.416128 | 12.20749 | 0.565044 |
| DALYs (Disability-Adjusted Life Years) | Antigua and Barbuda | Both | 55+ years | Liver cancer | Drug use | Number | 2021 | 6.172591 | 12.66283 | 0.837409 |
| DALYs (Disability-Adjusted Life Years) | Commonwealth of Dominica | Both | 55+ years | Liver cancer | Drug use | Number | 2021 | 6.046334 | 13.15191 | 0.979701 |
| DALYs (Disability-Adjusted Life Years) | Saint Kitts and Nevis | Both | 55+ years | Liver cancer | Drug use | Number | 2021 | 5.271008 | 11.01922 | 0.982667 |
| DALYs (Disability-Adjusted Life Years) | Republic of Palau | Both | 55+ years | Liver cancer | Drug use | Number | 2021 | 4.71992 | 8.22674 | 1.093624 |
| DALYs (Disability-Adjusted Life Years) | Republic of the Marshall Islands | Both | 55+ years | Liver cancer | Drug use | Number | 2021 | 4.435515 | 9.393442 | 0.455832 |
| DALYs (Disability-Adjusted Life Years) | Bermuda | Both | 55+ years | Liver cancer | Drug use | Number | 2021 | 4.064934 | 7.597077 | 0.723898 |
| DALYs (Disability-Adjusted Life Years) | Tuvalu | Both | 55+ years | Liver cancer | Drug use | Number | 2021 | 1.682337 | 3.213434 | 0.284009 |
| DALYs (Disability-Adjusted Life Years) | Republic of Nauru | Both | 55+ years | Liver cancer | Drug use | Number | 2021 | 0.903737 | 1.687908 | 0.143683 |
| DALYs (Disability-Adjusted Life Years) | Democratic Republic of Sao Tome and Principe | Both | 55+ years | Liver cancer | Drug use | Number | 2021 | 0.461924 | 1.205485 | 0.048931 |
| DALYs (Disability-Adjusted Life Years) | Republic of Niue | Both | 55+ years | Liver cancer | Drug use | Number | 2021 | 0.366555 | 0.64731 | 0.077442 |
| DALYs (Disability-Adjusted Life Years) | Tokelau | Both | 55+ years | Liver cancer | Drug use | Number | 2021 | 0.227531 | 0.453921 | 0.019146 |

| measure | location | sex | age | cause | rei | metric | year | val | upper | lower |
| --- | --- | --- | --- | --- | --- | --- | --- | --- | --- | --- |
| DALYs (Disability-Adjusted Life Years) | Mongolia | Both | Age-standardized | Liver cancer | Drug use | Rate | 2021 | 312.4264 | 599.8936 | 20.67862 |
| DALYs (Disability-Adjusted Life Years) | Kingdom of Tonga | Both | Age-standardized | Liver cancer | Drug use | Rate | 2021 | 62.54151 | 108.4578 | 8.335952 |
| DALYs (Disability-Adjusted Life Years) | Kingdom of Spain | Both | Age-standardized | Liver cancer | Drug use | Rate | 2021 | 39.51003 | 55.69932 | 9.118514 |
| DALYs (Disability-Adjusted Life Years) | Republic of Serbia | Both | Age-standardized | Liver cancer | Drug use | Rate | 2021 | 37.64166 | 58.75163 | 10.64974 |
| DALYs (Disability-Adjusted Life Years) | Republic of Azerbaijan | Both | Age-standardized | Liver cancer | Drug use | Rate | 2021 | 37.24462 | 80.23901 | 3.54955 |
| DALYs (Disability-Adjusted Life Years) | French Republic | Both | Age-standardized | Liver cancer | Drug use | Rate | 2021 | 35.14762 | 53.20919 | 3.63627 |
| DALYs (Disability-Adjusted Life Years) | Principality of Andorra | Both | Age-standardized | Liver cancer | Drug use | Rate | 2021 | 32.75297 | 64.02448 | 4.987539 |
| DALYs (Disability-Adjusted Life Years) | Principality of Monaco | Both | Age-standardized | Liver cancer | Drug use | Rate | 2021 | 31.68078 | 58.23765 | 2.019582 |
| DALYs (Disability-Adjusted Life Years) | United States of America | Both | Age-standardized | Liver cancer | Drug use | Rate | 2021 | 31.46611 | 36.76662 | 26.44946 |
| DALYs (Disability-Adjusted Life Years) | Republic of Kazakhstan | Both | Age-standardized | Liver cancer | Drug use | Rate | 2021 | 31.38514 | 47.05566 | 5.470522 |
| DALYs (Disability-Adjusted Life Years) | Turkmenistan | Both | Age-standardized | Liver cancer | Drug use | Rate | 2021 | 29.11678 | 53.64376 | 2.803927 |
| DALYs (Disability-Adjusted Life Years) | Cook Islands | Both | Age-standardized | Liver cancer | Drug use | Rate | 2021 | 29.11381 | 49.36841 | 5.310037 |
| DALYs (Disability-Adjusted Life Years) | Japan | Both | Age-standardized | Liver cancer | Drug use | Rate | 2021 | 28.57659 | 39.6574 | 18.73898 |
| DALYs (Disability-Adjusted Life Years) | Republic of Uzbekistan | Both | Age-standardized | Liver cancer | Drug use | Rate | 2021 | 28.20397 | 46.72676 | 1.968553 |
| DALYs (Disability-Adjusted Life Years) | United Kingdom of Great Britain and Northern Ireland | Both | Age-standardized | Liver cancer | Drug use | Rate | 2021 | 26.54434 | 32.77162 | 20.46639 |
| DALYs (Disability-Adjusted Life Years) | Republic of Tajikistan | Both | Age-standardized | Liver cancer | Drug use | Rate | 2021 | 25.96684 | 51.03889 | 2.604232 |
| DALYs (Disability-Adjusted Life Years) | Republic of Italy | Both | Age-standardized | Liver cancer | Drug use | Rate | 2021 | 25.09324 | 33.35327 | 17.15445 |
| DALYs (Disability-Adjusted Life Years) | North Macedonia | Both | Age-standardized | Liver cancer | Drug use | Rate | 2021 | 24.4458 | 42.51304 | 1.848181 |
| DALYs (Disability-Adjusted Life Years) | Brunei Darussalam | Both | Age-standardized | Liver cancer | Drug use | Rate | 2021 | 23.70384 | 46.49676 | 2.866444 |
| DALYs (Disability-Adjusted Life Years) | Socialist Republic of Viet Nam | Both | Age-standardized | Liver cancer | Drug use | Rate | 2021 | 23.67281 | 46.59234 | 2.554043 |
| DALYs (Disability-Adjusted Life Years) | Kyrgyz Republic | Both | Age-standardized | Liver cancer | Drug use | Rate | 2021 | 23.53649 | 37.76627 | 3.601165 |
| DALYs (Disability-Adjusted Life Years) | Republic of Mozambique | Both | Age-standardized | Liver cancer | Drug use | Rate | 2021 | 22.88753 | 63.58625 | 1.570642 |
| DALYs (Disability-Adjusted Life Years) | Republic of Albania | Both | Age-standardized | Liver cancer | Drug use | Rate | 2021 | 22.40923 | 43.18017 | 2.230894 |
| DALYs (Disability-Adjusted Life Years) | Republic of Kiribati | Both | Age-standardized | Liver cancer | Drug use | Rate | 2021 | 21.8604 | 41.80201 | 2.356518 |
| DALYs (Disability-Adjusted Life Years) | Australia | Both | Age-standardized | Liver cancer | Drug use | Rate | 2021 | 21.41169 | 34.09975 | 0.960905 |
| DALYs (Disability-Adjusted Life Years) | Swiss Confederation | Both | Age-standardized | Liver cancer | Drug use | Rate | 2021 | 21.27966 | 31.91518 | 4.617545 |
| DALYs (Disability-Adjusted Life Years) | Grand Duchy of Luxembourg | Both | Age-standardized | Liver cancer | Drug use | Rate | 2021 | 20.99145 | 31.53739 | 1.689202 |
| DALYs (Disability-Adjusted Life Years) | Republic of Maldives | Both | Age-standardized | Liver cancer | Drug use | Rate | 2021 | 20.0863 | 31.81359 | 6.871475 |
| DALYs (Disability-Adjusted Life Years) | Georgia | Both | Age-standardized | Liver cancer | Drug use | Rate | 2021 | 19.97962 | 32.07565 | 2.810061 |
| DALYs (Disability-Adjusted Life Years) | Kingdom of Norway | Both | Age-standardized | Liver cancer | Drug use | Rate | 2021 | 19.90459 | 25.25515 | 14.62053 |
| DALYs (Disability-Adjusted Life Years) | Northern Mariana Islands | Both | Age-standardized | Liver cancer | Drug use | Rate | 2021 | 19.85274 | 32.65812 | 3.595593 |
| DALYs (Disability-Adjusted Life Years) | American Samoa | Both | Age-standardized | Liver cancer | Drug use | Rate | 2021 | 19.70165 | 33.31116 | 3.413652 |
| DALYs (Disability-Adjusted Life Years) | Montenegro | Both | Age-standardized | Liver cancer | Drug use | Rate | 2021 | 19.2627 | 33.9509 | 3.334042 |
| DALYs (Disability-Adjusted Life Years) | Republic of Estonia | Both | Age-standardized | Liver cancer | Drug use | Rate | 2021 | 19.22293 | 29.57743 | 5.349048 |
| DALYs (Disability-Adjusted Life Years) | Republic of Palau | Both | Age-standardized | Liver cancer | Drug use | Rate | 2021 | 19.05283 | 33.20876 | 4.414618 |
| DALYs (Disability-Adjusted Life Years) | Republic of Iceland | Both | Age-standardized | Liver cancer | Drug use | Rate | 2021 | 18.98378 | 29.30275 | 3.725467 |
| DALYs (Disability-Adjusted Life Years) | Taiwan (Province of China) | Both | Age-standardized | Liver cancer | Drug use | Rate | 2021 | 18.29693 | 39.2605 | 1.292445 |
| DALYs (Disability-Adjusted Life Years) | Republic of Fiji | Both | Age-standardized | Liver cancer | Drug use | Rate | 2021 | 17.12528 | 28.99132 | 2.799351 |
| DALYs (Disability-Adjusted Life Years) | People's Republic of China | Both | Age-standardized | Liver cancer | Drug use | Rate | 2021 | 17.11503 | 23.79682 | 10.97921 |
| DALYs (Disability-Adjusted Life Years) | Kingdom of Thailand | Both | Age-standardized | Liver cancer | Drug use | Rate | 2021 | 16.97032 | 30.71473 | 1.885236 |
| DALYs (Disability-Adjusted Life Years) | Lao People's Democratic Republic | Both | Age-standardized | Liver cancer | Drug use | Rate | 2021 | 16.92349 | 33.65879 | 2.51762 |
| DALYs (Disability-Adjusted Life Years) | Republic of Nauru | Both | Age-standardized | Liver cancer | Drug use | Rate | 2021 | 16.52735 | 30.86811 | 2.627638 |
| DALYs (Disability-Adjusted Life Years) | Federated States of Micronesia | Both | Age-standardized | Liver cancer | Drug use | Rate | 2021 | 16.45725 | 31.75111 | 2.097036 |
| DALYs (Disability-Adjusted Life Years) | Portuguese Republic | Both | Age-standardized | Liver cancer | Drug use | Rate | 2021 | 16.215 | 28.51435 | 1.3725 |
| DALYs (Disability-Adjusted Life Years) | Republic of Slovenia | Both | Age-standardized | Liver cancer | Drug use | Rate | 2021 | 16.09599 | 25.8697 | 2.012699 |
| DALYs (Disability-Adjusted Life Years) | Republic of Belarus | Both | Age-standardized | Liver cancer | Drug use | Rate | 2021 | 16.00255 | 24.87372 | 6.697639 |
| DALYs (Disability-Adjusted Life Years) | Republic of Niue | Both | Age-standardized | Liver cancer | Drug use | Rate | 2021 | 15.83555 | 27.96451 | 3.34558 |
| DALYs (Disability-Adjusted Life Years) | Republic of Korea | Both | Age-standardized | Liver cancer | Drug use | Rate | 2021 | 15.76941 | 31.13498 | 2.727519 |
| DALYs (Disability-Adjusted Life Years) | Tuvalu | Both | Age-standardized | Liver cancer | Drug use | Rate | 2021 | 15.60466 | 29.80648 | 2.63435 |
| DALYs (Disability-Adjusted Life Years) | Independent State of Samoa | Both | Age-standardized | Liver cancer | Drug use | Rate | 2021 | 15.36657 | 27.44428 | 1.678121 |
| DALYs (Disability-Adjusted Life Years) | Guam | Both | Age-standardized | Liver cancer | Drug use | Rate | 2021 | 15.35415 | 23.9199 | 2.435142 |
| DALYs (Disability-Adjusted Life Years) | New Zealand | Both | Age-standardized | Liver cancer | Drug use | Rate | 2021 | 15.27047 | 20.39647 | 6.352131 |
| DALYs (Disability-Adjusted Life Years) | Tokelau | Both | Age-standardized | Liver cancer | Drug use | Rate | 2021 | 15.21702 | 30.35768 | 1.28049 |
| DALYs (Disability-Adjusted Life Years) | Federal Republic of Germany | Both | Age-standardized | Liver cancer | Drug use | Rate | 2021 | 15.02569 | 24.87648 | 3.097825 |
| DALYs (Disability-Adjusted Life Years) | Republic of Moldova | Both | Age-standardized | Liver cancer | Drug use | Rate | 2021 | 14.58503 | 23.18938 | 3.329929 |
| DALYs (Disability-Adjusted Life Years) | Bosnia and Herzegovina | Both | Age-standardized | Liver cancer | Drug use | Rate | 2021 | 14.42782 | 27.50665 | 2.330344 |
| DALYs (Disability-Adjusted Life Years) | Solomon Islands | Both | Age-standardized | Liver cancer | Drug use | Rate | 2021 | 14.27014 | 29.35242 | 1.094151 |
| DALYs (Disability-Adjusted Life Years) | Republic of Malta | Both | Age-standardized | Liver cancer | Drug use | Rate | 2021 | 14.20047 | 22.49205 | 1.399596 |
| DALYs (Disability-Adjusted Life Years) | Republic of the Philippines | Both | Age-standardized | Liver cancer | Drug use | Rate | 2021 | 13.78028 | 19.22588 | 9.114935 |
| DALYs (Disability-Adjusted Life Years) | Republic of Vanuatu | Both | Age-standardized | Liver cancer | Drug use | Rate | 2021 | 13.53163 | 26.84186 | 1.47305 |
| DALYs (Disability-Adjusted Life Years) | Democratic People's Republic of Korea | Both | Age-standardized | Liver cancer | Drug use | Rate | 2021 | 13.34857 | 26.66812 | 0.924553 |
| DALYs (Disability-Adjusted Life Years) | Republic of Costa Rica | Both | Age-standardized | Liver cancer | Drug use | Rate | 2021 | 13.25426 | 25.5558 | 1.793079 |
| DALYs (Disability-Adjusted Life Years) | Ireland | Both | Age-standardized | Liver cancer | Drug use | Rate | 2021 | 13.12265 | 21.4436 | 1.819762 |
| DALYs (Disability-Adjusted Life Years) | Kingdom of Belgium | Both | Age-standardized | Liver cancer | Drug use | Rate | 2021 | 12.94924 | 23.85702 | 0.844498 |
| DALYs (Disability-Adjusted Life Years) | Republic of the Marshall Islands | Both | Age-standardized | Liver cancer | Drug use | Rate | 2021 | 12.79632 | 27.09977 | 1.31506 |
| DALYs (Disability-Adjusted Life Years) | Islamic Republic of Pakistan | Both | Age-standardized | Liver cancer | Drug use | Rate | 2021 | 12.78578 | 25.44094 | 4.169991 |
| DALYs (Disability-Adjusted Life Years) | State of Israel | Both | Age-standardized | Liver cancer | Drug use | Rate | 2021 | 12.01301 | 19.59043 | 1.19905 |
| DALYs (Disability-Adjusted Life Years) | Republic of Chile | Both | Age-standardized | Liver cancer | Drug use | Rate | 2021 | 11.93232 | 22.31466 | 0.843448 |
| DALYs (Disability-Adjusted Life Years) | Russian Federation | Both | Age-standardized | Liver cancer | Drug use | Rate | 2021 | 11.56867 | 14.48837 | 8.749806 |
| DALYs (Disability-Adjusted Life Years) | Republic of the Union of Myanmar | Both | Age-standardized | Liver cancer | Drug use | Rate | 2021 | 11.23743 | 26.38409 | 0.658849 |
| DALYs (Disability-Adjusted Life Years) | Republic of Paraguay | Both | Age-standardized | Liver cancer | Drug use | Rate | 2021 | 10.7795 | 20.82293 | 1.645366 |
| DALYs (Disability-Adjusted Life Years) | Greenland | Both | Age-standardized | Liver cancer | Drug use | Rate | 2021 | 10.61837 | 21.14958 | 1.547719 |
| DALYs (Disability-Adjusted Life Years) | Slovak Republic | Both | Age-standardized | Liver cancer | Drug use | Rate | 2021 | 10.24867 | 18.95698 | 1.398564 |
| DALYs (Disability-Adjusted Life Years) | Malaysia | Both | Age-standardized | Liver cancer | Drug use | Rate | 2021 | 10.17846 | 19.25822 | 1.021693 |
| DALYs (Disability-Adjusted Life Years) | Republic of Seychelles | Both | Age-standardized | Liver cancer | Drug use | Rate | 2021 | 10.01359 | 19.70254 | 1.078702 |
| DALYs (Disability-Adjusted Life Years) | Republic of Singapore | Both | Age-standardized | Liver cancer | Drug use | Rate | 2021 | 9.77616 | 19.20306 | 1.615457 |
| DALYs (Disability-Adjusted Life Years) | United Arab Emirates | Both | Age-standardized | Liver cancer | Drug use | Rate | 2021 | 9.471422 | 23.70742 | 0.844953 |
| DALYs (Disability-Adjusted Life Years) | Republic of Finland | Both | Age-standardized | Liver cancer | Drug use | Rate | 2021 | 9.356399 | 18.83301 | 1.089654 |
| DALYs (Disability-Adjusted Life Years) | Republic of San Marino | Both | Age-standardized | Liver cancer | Drug use | Rate | 2021 | 9.169853 | 17.4468 | 0.807555 |
| DALYs (Disability-Adjusted Life Years) | Republic of Guatemala | Both | Age-standardized | Liver cancer | Drug use | Rate | 2021 | 8.843554 | 20.11657 | 0.718745 |
| DALYs (Disability-Adjusted Life Years) | Republic of Mali | Both | Age-standardized | Liver cancer | Drug use | Rate | 2021 | 8.805851 | 21.18689 | 1.065849 |
| DALYs (Disability-Adjusted Life Years) | Republic of Austria | Both | Age-standardized | Liver cancer | Drug use | Rate | 2021 | 8.801243 | 17.0295 | 1.121115 |
| DALYs (Disability-Adjusted Life Years) | Republic of Latvia | Both | Age-standardized | Liver cancer | Drug use | Rate | 2021 | 8.788493 | 16.44975 | 1.286464 |
| DALYs (Disability-Adjusted Life Years) | Independent State of Papua New Guinea | Both | Age-standardized | Liver cancer | Drug use | Rate | 2021 | 8.633728 | 23.11442 | 0.454958 |
| DALYs (Disability-Adjusted Life Years) | Republic of Bulgaria | Both | Age-standardized | Liver cancer | Drug use | Rate | 2021 | 8.519843 | 15.89983 | 1.510739 |
| DALYs (Disability-Adjusted Life Years) | United Mexican States | Both | Age-standardized | Liver cancer | Drug use | Rate | 2021 | 8.406214 | 12.59354 | 4.978128 |
| DALYs (Disability-Adjusted Life Years) | Democratic Republic of Timor-Leste | Both | Age-standardized | Liver cancer | Drug use | Rate | 2021 | 8.205909 | 16.41774 | 1.039908 |
| DALYs (Disability-Adjusted Life Years) | Federal Democratic Republic of Nepal | Both | Age-standardized | Liver cancer | Drug use | Rate | 2021 | 7.963146 | 16.68939 | 1.044307 |
| DALYs (Disability-Adjusted Life Years) | State of Qatar | Both | Age-standardized | Liver cancer | Drug use | Rate | 2021 | 7.955077 | 18.21792 | 0.734981 |
| DALYs (Disability-Adjusted Life Years) | Kingdom of Denmark | Both | Age-standardized | Liver cancer | Drug use | Rate | 2021 | 7.779782 | 14.58316 | 0.652099 |
| DALYs (Disability-Adjusted Life Years) | Kingdom of Bhutan | Both | Age-standardized | Liver cancer | Drug use | Rate | 2021 | 7.532334 | 18.38322 | 0.324671 |
| DALYs (Disability-Adjusted Life Years) | Republic of India | Both | Age-standardized | Liver cancer | Drug use | Rate | 2021 | 7.530609 | 10.06559 | 5.407564 |
| DALYs (Disability-Adjusted Life Years) | Republic of Armenia | Both | Age-standardized | Liver cancer | Drug use | Rate | 2021 | 7.482048 | 15.74629 | 0.725465 |
| DALYs (Disability-Adjusted Life Years) | Republic of Panama | Both | Age-standardized | Liver cancer | Drug use | Rate | 2021 | 7.379669 | 14.18317 | 0.730948 |
| DALYs (Disability-Adjusted Life Years) | Republic of Lithuania | Both | Age-standardized | Liver cancer | Drug use | Rate | 2021 | 7.207124 | 14.03932 | 1.129284 |
| DALYs (Disability-Adjusted Life Years) | Republic of the Gambia | Both | Age-standardized | Liver cancer | Drug use | Rate | 2021 | 7.149771 | 17.11551 | 1.206249 |
| DALYs (Disability-Adjusted Life Years) | Kingdom of Cambodia | Both | Age-standardized | Liver cancer | Drug use | Rate | 2021 | 7.105425 | 19.37934 | 0.385107 |
| DALYs (Disability-Adjusted Life Years) | Saint Kitts and Nevis | Both | Age-standardized | Liver cancer | Drug use | Rate | 2021 | 6.917721 | 14.46173 | 1.289662 |
| DALYs (Disability-Adjusted Life Years) | United Republic of Tanzania | Both | Age-standardized | Liver cancer | Drug use | Rate | 2021 | 6.89963 | 15.35613 | 0.804023 |
| DALYs (Disability-Adjusted Life Years) | Commonwealth of Dominica | Both | Age-standardized | Liver cancer | Drug use | Rate | 2021 | 6.776008 | 14.73909 | 1.097931 |
| DALYs (Disability-Adjusted Life Years) | Kingdom of the Netherlands | Both | Age-standardized | Liver cancer | Drug use | Rate | 2021 | 6.734904 | 11.8848 | 0.710911 |
| DALYs (Disability-Adjusted Life Years) | Federative Republic of Brazil | Both | Age-standardized | Liver cancer | Drug use | Rate | 2021 | 6.663909 | 9.782735 | 3.920591 |
| DALYs (Disability-Adjusted Life Years) | Grenada | Both | Age-standardized | Liver cancer | Drug use | Rate | 2021 | 6.628197 | 13.39091 | 1.128128 |
| DALYs (Disability-Adjusted Life Years) | Republic of Honduras | Both | Age-standardized | Liver cancer | Drug use | Rate | 2021 | 6.592927 | 14.40291 | 0.442027 |
| DALYs (Disability-Adjusted Life Years) | Kingdom of Sweden | Both | Age-standardized | Liver cancer | Drug use | Rate | 2021 | 6.564236 | 11.50321 | 2.165693 |
| DALYs (Disability-Adjusted Life Years) | Arab Republic of Egypt | Both | Age-standardized | Liver cancer | Drug use | Rate | 2021 | 6.270975 | 13.99531 | 0.455876 |
| DALYs (Disability-Adjusted Life Years) | Commonwealth of the Bahamas | Both | Age-standardized | Liver cancer | Drug use | Rate | 2021 | 6.089078 | 12.65558 | 0.835724 |
| DALYs (Disability-Adjusted Life Years) | Romania | Both | Age-standardized | Liver cancer | Drug use | Rate | 2021 | 6.033972 | 12.0923 | 0.403892 |
| DALYs (Disability-Adjusted Life Years) | Czech Republic | Both | Age-standardized | Liver cancer | Drug use | Rate | 2021 | 6.016139 | 10.8258 | 0.996348 |
| DALYs (Disability-Adjusted Life Years) | Hellenic Republic | Both | Age-standardized | Liver cancer | Drug use | Rate | 2021 | 5.933719 | 11.26041 | 0.888039 |
| DALYs (Disability-Adjusted Life Years) | Bolivarian Republic of Venezuela | Both | Age-standardized | Liver cancer | Drug use | Rate | 2021 | 5.737989 | 11.58639 | 0.371996 |
| DALYs (Disability-Adjusted Life Years) | Antigua and Barbuda | Both | Age-standardized | Liver cancer | Drug use | Rate | 2021 | 5.529858 | 11.34429 | 0.750212 |
| DALYs (Disability-Adjusted Life Years) | Kingdom of Eswatini | Both | Age-standardized | Liver cancer | Drug use | Rate | 2021 | 5.529178 | 16.99191 | 0.601693 |
| DALYs (Disability-Adjusted Life Years) | Republic of Suriname | Both | Age-standardized | Liver cancer | Drug use | Rate | 2021 | 5.298694 | 10.97586 | 0.75322 |
| DALYs (Disability-Adjusted Life Years) | Republic of Nicaragua | Both | Age-standardized | Liver cancer | Drug use | Rate | 2021 | 5.28473 | 11.57296 | 0.425955 |
| DALYs (Disability-Adjusted Life Years) | Puerto Rico | Both | Age-standardized | Liver cancer | Drug use | Rate | 2021 | 5.200667 | 10.76783 | 0.777591 |
| DALYs (Disability-Adjusted Life Years) | United States Virgin Islands | Both | Age-standardized | Liver cancer | Drug use | Rate | 2021 | 5.121987 | 10.45304 | 0.588824 |
| DALYs (Disability-Adjusted Life Years) | Ukraine | Both | Age-standardized | Liver cancer | Drug use | Rate | 2021 | 5.099501 | 8.635561 | 1.681353 |
| DALYs (Disability-Adjusted Life Years) | Islamic Republic of Iran | Both | Age-standardized | Liver cancer | Drug use | Rate | 2021 | 5.083206 | 7.066713 | 3.477236 |
| DALYs (Disability-Adjusted Life Years) | Republic of Poland | Both | Age-standardized | Liver cancer | Drug use | Rate | 2021 | 5.074835 | 6.670176 | 3.60036 |
| DALYs (Disability-Adjusted Life Years) | Saint Vincent and the Grenadines | Both | Age-standardized | Liver cancer | Drug use | Rate | 2021 | 5.032361 | 9.678849 | 0.624687 |
| DALYs (Disability-Adjusted Life Years) | Kingdom of Lesotho | Both | Age-standardized | Liver cancer | Drug use | Rate | 2021 | 4.748102 | 14.33918 | 0.341422 |
| DALYs (Disability-Adjusted Life Years) | Barbados | Both | Age-standardized | Liver cancer | Drug use | Rate | 2021 | 4.713736 | 9.761257 | 0.86579 |
| DALYs (Disability-Adjusted Life Years) | Republic of Indonesia | Both | Age-standardized | Liver cancer | Drug use | Rate | 2021 | 4.610272 | 8.892229 | 2.01773 |
| DALYs (Disability-Adjusted Life Years) | Belize | Both | Age-standardized | Liver cancer | Drug use | Rate | 2021 | 4.429682 | 8.891939 | 0.769827 |
| DALYs (Disability-Adjusted Life Years) | Republic of Trinidad and Tobago | Both | Age-standardized | Liver cancer | Drug use | Rate | 2021 | 4.401302 | 9.08257 | 0.639777 |
| DALYs (Disability-Adjusted Life Years) | Republic of Zimbabwe | Both | Age-standardized | Liver cancer | Drug use | Rate | 2021 | 4.397092 | 10.01651 | 0.246038 |
| DALYs (Disability-Adjusted Life Years) | Republic of Guinea-Bissau | Both | Age-standardized | Liver cancer | Drug use | Rate | 2021 | 4.143107 | 9.256671 | 0.501047 |
| DALYs (Disability-Adjusted Life Years) | Republic of Guinea | Both | Age-standardized | Liver cancer | Drug use | Rate | 2021 | 4.030217 | 9.863145 | 0.657894 |
| DALYs (Disability-Adjusted Life Years) | Republic of Croatia | Both | Age-standardized | Liver cancer | Drug use | Rate | 2021 | 3.97291 | 7.240249 | 0.538658 |
| DALYs (Disability-Adjusted Life Years) | Islamic Republic of Mauritania | Both | Age-standardized | Liver cancer | Drug use | Rate | 2021 | 3.965839 | 9.907594 | 0.415306 |
| DALYs (Disability-Adjusted Life Years) | Eastern Republic of Uruguay | Both | Age-standardized | Liver cancer | Drug use | Rate | 2021 | 3.838603 | 8.839688 | 0.470194 |
| DALYs (Disability-Adjusted Life Years) | State of Libya | Both | Age-standardized | Liver cancer | Drug use | Rate | 2021 | 3.83345 | 8.604439 | 0.355492 |
| DALYs (Disability-Adjusted Life Years) | Jamaica | Both | Age-standardized | Liver cancer | Drug use | Rate | 2021 | 3.768141 | 7.445994 | 0.701764 |
| DALYs (Disability-Adjusted Life Years) | Republic of Cyprus | Both | Age-standardized | Liver cancer | Drug use | Rate | 2021 | 3.678197 | 7.906206 | 0.188205 |
| DALYs (Disability-Adjusted Life Years) | Republic of Guyana | Both | Age-standardized | Liver cancer | Drug use | Rate | 2021 | 3.639017 | 8.304292 | 0.399966 |
| DALYs (Disability-Adjusted Life Years) | Plurinational State of Bolivia | Both | Age-standardized | Liver cancer | Drug use | Rate | 2021 | 3.620848 | 7.9996 | 0.643747 |
| DALYs (Disability-Adjusted Life Years) | Canada | Both | Age-standardized | Liver cancer | Drug use | Rate | 2021 | 3.522825 | 7.059599 | 0.496182 |
| DALYs (Disability-Adjusted Life Years) | Democratic Socialist Republic of Sri Lanka | Both | Age-standardized | Liver cancer | Drug use | Rate | 2021 | 3.462038 | 7.064283 | 0.321725 |
| DALYs (Disability-Adjusted Life Years) | Central African Republic | Both | Age-standardized | Liver cancer | Drug use | Rate | 2021 | 3.329854 | 9.546188 | 0.331537 |
| DALYs (Disability-Adjusted Life Years) | Republic of Colombia | Both | Age-standardized | Liver cancer | Drug use | Rate | 2021 | 3.305431 | 6.797995 | 0.275881 |
| DALYs (Disability-Adjusted Life Years) | Syrian Arab Republic | Both | Age-standardized | Liver cancer | Drug use | Rate | 2021 | 3.292946 | 7.684893 | 0.451923 |
| DALYs (Disability-Adjusted Life Years) | Republic of Cuba | Both | Age-standardized | Liver cancer | Drug use | Rate | 2021 | 3.267747 | 6.239255 | 0.558941 |
| DALYs (Disability-Adjusted Life Years) | Palestine | Both | Age-standardized | Liver cancer | Drug use | Rate | 2021 | 3.252226 | 7.513785 | 0.228866 |
| DALYs (Disability-Adjusted Life Years) | Republic of El Salvador | Both | Age-standardized | Liver cancer | Drug use | Rate | 2021 | 3.188793 | 6.484126 | 0.537607 |
| DALYs (Disability-Adjusted Life Years) | Republic of Ecuador | Both | Age-standardized | Liver cancer | Drug use | Rate | 2021 | 3.173313 | 6.477787 | 0.44729 |
| DALYs (Disability-Adjusted Life Years) | Republic of Mauritius | Both | Age-standardized | Liver cancer | Drug use | Rate | 2021 | 3.157049 | 4.713263 | 0.764799 |
| DALYs (Disability-Adjusted Life Years) | Gabonese Republic | Both | Age-standardized | Liver cancer | Drug use | Rate | 2021 | 3.151318 | 7.972035 | 0.210969 |
| DALYs (Disability-Adjusted Life Years) | Dominican Republic | Both | Age-standardized | Liver cancer | Drug use | Rate | 2021 | 3.138471 | 6.60614 | 0.505401 |
| DALYs (Disability-Adjusted Life Years) | Burkina Faso | Both | Age-standardized | Liver cancer | Drug use | Rate | 2021 | 3.113735 | 8.028584 | 0.362114 |
| DALYs (Disability-Adjusted Life Years) | Republic of Liberia | Both | Age-standardized | Liver cancer | Drug use | Rate | 2021 | 3.004563 | 7.134141 | 0.247372 |
| DALYs (Disability-Adjusted Life Years) | Republic of Iraq | Both | Age-standardized | Liver cancer | Drug use | Rate | 2021 | 2.952746 | 6.73139 | 0.333341 |
| DALYs (Disability-Adjusted Life Years) | Republic of Angola | Both | Age-standardized | Liver cancer | Drug use | Rate | 2021 | 2.950884 | 9.741491 | 0.169514 |
| DALYs (Disability-Adjusted Life Years) | Bermuda | Both | Age-standardized | Liver cancer | Drug use | Rate | 2021 | 2.950066 | 5.513467 | 0.525358 |
| DALYs (Disability-Adjusted Life Years) | Kingdom of Saudi Arabia | Both | Age-standardized | Liver cancer | Drug use | Rate | 2021 | 2.918526 | 7.09677 | 0.266172 |
| DALYs (Disability-Adjusted Life Years) | Argentine Republic | Both | Age-standardized | Liver cancer | Drug use | Rate | 2021 | 2.885566 | 5.717147 | 0.254376 |
| DALYs (Disability-Adjusted Life Years) | Kingdom of Bahrain | Both | Age-standardized | Liver cancer | Drug use | Rate | 2021 | 2.835534 | 7.021533 | 0.191959 |
| DALYs (Disability-Adjusted Life Years) | Republic of the Congo | Both | Age-standardized | Liver cancer | Drug use | Rate | 2021 | 2.802101 | 7.586808 | 0.205406 |
| DALYs (Disability-Adjusted Life Years) | Republic of Chad | Both | Age-standardized | Liver cancer | Drug use | Rate | 2021 | 2.781243 | 7.079052 | 0.471733 |
| DALYs (Disability-Adjusted Life Years) | Saint Lucia | Both | Age-standardized | Liver cancer | Drug use | Rate | 2021 | 2.686837 | 5.370688 | 0.399856 |
| DALYs (Disability-Adjusted Life Years) | Sultanate of Oman | Both | Age-standardized | Liver cancer | Drug use | Rate | 2021 | 2.646631 | 6.167706 | 0.225897 |
| DALYs (Disability-Adjusted Life Years) | Republic of Benin | Both | Age-standardized | Liver cancer | Drug use | Rate | 2021 | 2.624085 | 6.323157 | 0.348957 |
| DALYs (Disability-Adjusted Life Years) | Republic of Cabo Verde | Both | Age-standardized | Liver cancer | Drug use | Rate | 2021 | 2.425437 | 5.877241 | 0.341763 |
| DALYs (Disability-Adjusted Life Years) | Republic of Peru | Both | Age-standardized | Liver cancer | Drug use | Rate | 2021 | 2.389108 | 5.098626 | 0.403014 |
| DALYs (Disability-Adjusted Life Years) | Federal Republic of Somalia | Both | Age-standardized | Liver cancer | Drug use | Rate | 2021 | 2.349973 | 6.694347 | 0.185849 |
| DALYs (Disability-Adjusted Life Years) | Republic of Haiti | Both | Age-standardized | Liver cancer | Drug use | Rate | 2021 | 2.261373 | 6.165694 | 0.219824 |
| DALYs (Disability-Adjusted Life Years) | Republic of South Africa | Both | Age-standardized | Liver cancer | Drug use | Rate | 2021 | 2.260777 | 3.760585 | 1.244475 |
| DALYs (Disability-Adjusted Life Years) | Republic of Cameroon | Both | Age-standardized | Liver cancer | Drug use | Rate | 2021 | 2.207853 | 5.720807 | 0.316771 |
| DALYs (Disability-Adjusted Life Years) | Republic of the Niger | Both | Age-standardized | Liver cancer | Drug use | Rate | 2021 | 2.098555 | 5.385433 | 0.224425 |
| DALYs (Disability-Adjusted Life Years) | Republic of Tunisia | Both | Age-standardized | Liver cancer | Drug use | Rate | 2021 | 2.062059 | 5.234422 | 0.186377 |
| DALYs (Disability-Adjusted Life Years) | Republic of Equatorial Guinea | Both | Age-standardized | Liver cancer | Drug use | Rate | 2021 | 2.049111 | 5.004567 | 0.183306 |
| DALYs (Disability-Adjusted Life Years) | Republic of Sierra Leone | Both | Age-standardized | Liver cancer | Drug use | Rate | 2021 | 2.010159 | 4.716089 | 0.208957 |
| DALYs (Disability-Adjusted Life Years) | Republic of South Sudan | Both | Age-standardized | Liver cancer | Drug use | Rate | 2021 | 2.004354 | 4.874658 | 0.227753 |
| DALYs (Disability-Adjusted Life Years) | Togolese Republic | Both | Age-standardized | Liver cancer | Drug use | Rate | 2021 | 1.789218 | 4.469979 | 0.19198 |
| DALYs (Disability-Adjusted Life Years) | Hungary | Both | Age-standardized | Liver cancer | Drug use | Rate | 2021 | 1.781529 | 4.068267 | 0.132104 |
| DALYs (Disability-Adjusted Life Years) | Republic of Senegal | Both | Age-standardized | Liver cancer | Drug use | Rate | 2021 | 1.767624 | 3.973426 | 0.252288 |
| DALYs (Disability-Adjusted Life Years) | Republic of Sudan | Both | Age-standardized | Liver cancer | Drug use | Rate | 2021 | 1.744522 | 4.442358 | 0.142403 |
| DALYs (Disability-Adjusted Life Years) | Republic of Turkey | Both | Age-standardized | Liver cancer | Drug use | Rate | 2021 | 1.733938 | 4.111211 | 0.187852 |
| DALYs (Disability-Adjusted Life Years) | Republic of Botswana | Both | Age-standardized | Liver cancer | Drug use | Rate | 2021 | 1.704428 | 4.749229 | 0.228062 |
| DALYs (Disability-Adjusted Life Years) | Republic of Djibouti | Both | Age-standardized | Liver cancer | Drug use | Rate | 2021 | 1.494533 | 3.804337 | 0.147043 |
| DALYs (Disability-Adjusted Life Years) | Republic of Malawi | Both | Age-standardized | Liver cancer | Drug use | Rate | 2021 | 1.401619 | 3.244735 | 0.13377 |
| DALYs (Disability-Adjusted Life Years) | Union of the Comoros | Both | Age-standardized | Liver cancer | Drug use | Rate | 2021 | 1.390765 | 3.346219 | 0.120187 |
| DALYs (Disability-Adjusted Life Years) | Lebanese Republic | Both | Age-standardized | Liver cancer | Drug use | Rate | 2021 | 1.390381 | 3.198994 | 0.184524 |
| DALYs (Disability-Adjusted Life Years) | Republic of Ghana | Both | Age-standardized | Liver cancer | Drug use | Rate | 2021 | 1.387822 | 3.318748 | 0.160712 |
| DALYs (Disability-Adjusted Life Years) | Democratic Republic of the Congo | Both | Age-standardized | Liver cancer | Drug use | Rate | 2021 | 1.274658 | 3.695329 | 0.056149 |
| DALYs (Disability-Adjusted Life Years) | Hashemite Kingdom of Jordan | Both | Age-standardized | Liver cancer | Drug use | Rate | 2021 | 1.253151 | 3.071721 | 0.173484 |
| DALYs (Disability-Adjusted Life Years) | Islamic Republic of Afghanistan | Both | Age-standardized | Liver cancer | Drug use | Rate | 2021 | 1.210507 | 2.711579 | 0.177186 |
| DALYs (Disability-Adjusted Life Years) | People's Democratic Republic of Algeria | Both | Age-standardized | Liver cancer | Drug use | Rate | 2021 | 1.186988 | 2.707575 | 0.152818 |
| DALYs (Disability-Adjusted Life Years) | Republic of Madagascar | Both | Age-standardized | Liver cancer | Drug use | Rate | 2021 | 1.159406 | 2.782042 | 0.136649 |
| DALYs (Disability-Adjusted Life Years) | Republic of Zambia | Both | Age-standardized | Liver cancer | Drug use | Rate | 2021 | 1.129512 | 3.566235 | 0.10059 |
| DALYs (Disability-Adjusted Life Years) | Republic of Yemen | Both | Age-standardized | Liver cancer | Drug use | Rate | 2021 | 1.122878 | 2.801608 | 0.151571 |
| DALYs (Disability-Adjusted Life Years) | Federal Republic of Nigeria | Both | Age-standardized | Liver cancer | Drug use | Rate | 2021 | 1.040519 | 1.82751 | 0.561997 |
| DALYs (Disability-Adjusted Life Years) | State of Eritrea | Both | Age-standardized | Liver cancer | Drug use | Rate | 2021 | 1.025482 | 2.556607 | 0.143757 |
| DALYs (Disability-Adjusted Life Years) | Republic of Namibia | Both | Age-standardized | Liver cancer | Drug use | Rate | 2021 | 1.015651 | 2.403078 | 0.107481 |
| DALYs (Disability-Adjusted Life Years) | Republic of Rwanda | Both | Age-standardized | Liver cancer | Drug use | Rate | 2021 | 0.970411 | 2.275736 | 0.08709 |
| DALYs (Disability-Adjusted Life Years) | State of Kuwait | Both | Age-standardized | Liver cancer | Drug use | Rate | 2021 | 0.880603 | 1.918797 | 0.089865 |
| DALYs (Disability-Adjusted Life Years) | Republic of C么te d'Ivoire | Both | Age-standardized | Liver cancer | Drug use | Rate | 2021 | 0.853501 | 2.031858 | 0.073576 |
| DALYs (Disability-Adjusted Life Years) | Kingdom of Morocco | Both | Age-standardized | Liver cancer | Drug use | Rate | 2021 | 0.775253 | 1.713761 | 0.078989 |
| DALYs (Disability-Adjusted Life Years) | Republic of Kenya | Both | Age-standardized | Liver cancer | Drug use | Rate | 2021 | 0.744978 | 1.26895 | 0.402578 |
| DALYs (Disability-Adjusted Life Years) | Federal Democratic Republic of Ethiopia | Both | Age-standardized | Liver cancer | Drug use | Rate | 2021 | 0.735884 | 1.448886 | 0.330653 |
| DALYs (Disability-Adjusted Life Years) | Republic of Burundi | Both | Age-standardized | Liver cancer | Drug use | Rate | 2021 | 0.650553 | 1.680137 | 0.0791 |
| DALYs (Disability-Adjusted Life Years) | People's Republic of Bangladesh | Both | Age-standardized | Liver cancer | Drug use | Rate | 2021 | 0.615043 | 1.407778 | 0.018638 |
| DALYs (Disability-Adjusted Life Years) | Democratic Republic of Sao Tome and Principe | Both | Age-standardized | Liver cancer | Drug use | Rate | 2021 | 0.427038 | 1.114442 | 0.045236 |
| DALYs (Disability-Adjusted Life Years) | Republic of Uganda | Both | Age-standardized | Liver cancer | Drug use | Rate | 2021 | 0.117151 | 0.303872 | 0.013356 |

|  | location | EAPC | LCI | UCI | EAPC_CI |
| --- | --- | --- | --- | --- | --- |
| 1 | Taiwan (Province of China) | 3.72 | 0.38 | 7.16 | 3.72  (0.38 to 7.16) |
| 2 | People's Republic of China | 1.26 | -0.13 | 2.66 | 1.26  (-0.13 to 2.66) |
| 3 | Kingdom of Cambodia | 1.01 | -0.15 | 2.17 | 1.01  (-0.15 to 2.17) |
| 4 | Lao People's Democratic Republic | 0.64 | -1.93 | 3.28 | 0.64  (-1.93 to 3.28) |
| 5 | Republic of Indonesia | 1.57 | -0.26 | 3.43 | 1.57  (-0.26 to 3.43) |
| 6 | Democratic People's Republic of Korea | 1.23 | -0.81 | 3.32 | 1.23  (-0.81 to 3.32) |
| 7 | Republic of Maldives | 0.07 | -3.58 | 3.86 | 0.07  (-3.58 to 3.86) |
| 8 | Republic of the Philippines | 2.31 | -0.66 | 5.37 | 2.31  (-0.66 to 5.37) |
| 9 | Malaysia | 3.55 | 1.32 | 5.83 | 3.55  (1.32 to 5.83) |
| 10 | Kingdom of Thailand | 0.39 | -2.45 | 3.31 | 0.39  (-2.45 to 3.31) |
| 11 | Republic of the Union of Myanmar | 1.87 | 0.39 | 3.36 | 1.87  (0.39 to 3.36) |
| 12 | Democratic Socialist Republic of Sri Lanka | 1.37 | -1.14 | 3.94 | 1.37  (-1.14 to 3.94) |
| 13 | Socialist Republic of Viet Nam | 1.3 | -3.32 | 6.15 | 1.3  (-3.32 to 6.15) |
| 14 | Republic of Fiji | 2.05 | 0.66 | 3.45 | 2.05  (0.66 to 3.45) |
| 15 | Republic of the Marshall Islands | 2.03 | 1.42 | 2.65 | 2.03  (1.42 to 2.65) |
| 16 | Democratic Republic of Timor-Leste | 2.02 | 1.32 | 2.71 | 2.02  (1.32 to 2.71) |
| 17 | Independent State of Papua New Guinea | 0.21 | -1.1 | 1.54 | 0.21  (-1.1 to 1.54) |
| 18 | Republic of Kiribati | 1.35 | 0.39 | 2.31 | 1.35  (0.39 to 2.31) |
| 19 | Federated States of Micronesia | 1.63 | 0.41 | 2.86 | 1.63  (0.41 to 2.86) |
| 20 | Solomon Islands | 0.65 | -1.64 | 2.99 | 0.65  (-1.64 to 2.99) |
| 21 | Republic of Vanuatu | 1.69 | 0.01 | 3.4 | 1.69  (0.01 to 3.4) |
| 22 | Independent State of Samoa | 1.57 | -1.02 | 4.23 | 1.57  (-1.02 to 4.23) |
| 23 | Republic of Azerbaijan | 2.7 | 0.7 | 4.73 | 2.7  (0.7 to 4.73) |
| 24 | Kingdom of Tonga | 1.84 | -0.9 | 4.65 | 1.84  (-0.9 to 4.65) |
| 25 | Republic of Kazakhstan | -1.12 | -3.14 | 0.94 | -1.12  (-3.14 to 0.94) |
| 26 | Republic of Armenia | 2.33 | -0.09 | 4.81 | 2.33  (-0.09 to 4.81) |
| 27 | Georgia | 1.41 | -1.18 | 4.08 | 1.41  (-1.18 to 4.08) |
| 28 | Kyrgyz Republic | -0.19 | -1.99 | 1.64 | -0.19  (-1.99 to 1.64) |
| 29 | Republic of Tajikistan | 1.46 | 0.43 | 2.51 | 1.46  (0.43 to 2.51) |
| 30 | Mongolia | 2.51 | 0.31 | 4.75 | 2.51  (0.31 to 4.75) |
| 31 | Republic of Uzbekistan | 2.62 | 1.33 | 3.93 | 2.62  (1.33 to 3.93) |
| 32 | Republic of Albania | 1.59 | -0.71 | 3.94 | 1.59  (-0.71 to 3.94) |
| 33 | Turkmenistan | 2.12 | -0.97 | 5.3 | 2.12  (-0.97 to 5.3) |
| 34 | Republic of Bulgaria | -0.01 | -1.2 | 1.19 | -0.01  (-1.2 to 1.19) |
| 35 | Czech Republic | 1.23 | -0.43 | 2.92 | 1.23  (-0.43 to 2.92) |
| 36 | Bosnia and Herzegovina | 2.49 | -0.12 | 5.16 | 2.49  (-0.12 to 5.16) |
| 37 | Republic of Croatia | 3.69 | 2.15 | 5.26 | 3.69  (2.15 to 5.26) |
| 38 | North Macedonia | 1.88 | 0.35 | 3.42 | 1.88  (0.35 to 3.42) |
| 39 | Hungary | 2.42 | 0.47 | 4.42 | 2.42  (0.47 to 4.42) |
| 40 | Republic of Poland | 6.6 | 5.57 | 7.65 | 6.6  (5.57 to 7.65) |
| 41 | Montenegro | 3.22 | 1.6 | 4.87 | 3.22  (1.6 to 4.87) |
| 42 | Republic of Serbia | 1.69 | 0.77 | 2.62 | 1.69  (0.77 to 2.62) |
| 43 | Romania | 6.18 | 3.93 | 8.49 | 6.18  (3.93 to 8.49) |
| 44 | Republic of Slovenia | 3.03 | 1.09 | 5.01 | 3.03  (1.09 to 5.01) |
| 45 | Slovak Republic | 1.73 | 0.14 | 3.35 | 1.73  (0.14 to 3.35) |
| 46 | Republic of Belarus | 1.07 | -0.89 | 3.08 | 1.07  (-0.89 to 3.08) |
| 47 | Republic of Estonia | 1.99 | 0.62 | 3.39 | 1.99  (0.62 to 3.39) |
| 48 | Republic of Latvia | 3.09 | 2.35 | 3.83 | 3.09  (2.35 to 3.83) |
| 49 | Republic of Lithuania | 3.85 | 1.15 | 6.62 | 3.85  (1.15 to 6.62) |
| 50 | Republic of Moldova | 1.52 | -0.49 | 3.57 | 1.52  (-0.49 to 3.57) |
| 51 | Russian Federation | 3.13 | 1.17 | 5.13 | 3.13  (1.17 to 5.13) |
| 52 | Ukraine | 0.33 | -2.4 | 3.14 | 0.33  (-2.4 to 3.14) |
| 53 | Brunei Darussalam | 2.14 | -3.27 | 7.84 | 2.14  (-3.27 to 7.84) |
| 54 | Japan | 2.69 | -4.66 | 10.61 | 2.69  (-4.66 to 10.61) |
| 55 | Republic of Korea | 1.27 | -3.29 | 6.05 | 1.27  (-3.29 to 6.05) |
| 56 | Republic of Singapore | 2.39 | -3.64 | 8.79 | 2.39  (-3.64 to 8.79) |
| 57 | Australia | 7.01 | 3.89 | 10.21 | 7.01  (3.89 to 10.21) |
| 58 | New Zealand | 4.56 | 1.15 | 8.09 | 4.56  (1.15 to 8.09) |
| 59 | Principality of Andorra | 2.85 | 1.34 | 4.38 | 2.85  (1.34 to 4.38) |
| 60 | Republic of Austria | 6.33 | 2.35 | 10.46 | 6.33  (2.35 to 10.46) |
| 61 | Kingdom of Belgium | 4.57 | 1.31 | 7.94 | 4.57  (1.31 to 7.94) |
| 62 | Republic of Cyprus | 3.82 | 0.06 | 7.71 | 3.82  (0.06 to 7.71) |
| 63 | Kingdom of Denmark | 6.49 | 2.62 | 10.5 | 6.49  (2.62 to 10.5) |
| 64 | Republic of Finland | 5.01 | 1.78 | 8.33 | 5.01  (1.78 to 8.33) |
| 65 | French Republic | 4.1 | -1.49 | 10 | 4.1  (-1.49 to 10) |
| 66 | Federal Republic of Germany | 5.28 | 2.28 | 8.37 | 5.28  (2.28 to 8.37) |
| 67 | Hellenic Republic | 6.72 | 4.34 | 9.15 | 6.72  (4.34 to 9.15) |
| 68 | Republic of Iceland | 4.26 | 0.53 | 8.13 | 4.26  (0.53 to 8.13) |
| 69 | Ireland | 5.13 | 2.35 | 7.99 | 5.13  (2.35 to 7.99) |
| 70 | State of Israel | 2.99 | -0.25 | 6.32 | 2.99  (-0.25 to 6.32) |
| 71 | Republic of Italy | 1.77 | -3.51 | 7.33 | 1.77  (-3.51 to 7.33) |
| 72 | Grand Duchy of Luxembourg | 3.44 | 1.44 | 5.47 | 3.44  (1.44 to 5.47) |
| 73 | Republic of Malta | 4 | -0.08 | 8.24 | 4  (-0.08 to 8.24) |
| 74 | Kingdom of the Netherlands | 6.25 | 3.02 | 9.57 | 6.25  (3.02 to 9.57) |
| 75 | Kingdom of Norway | 4.65 | 3.17 | 6.16 | 4.65  (3.17 to 6.16) |
| 76 | Portuguese Republic | 5.74 | 1.28 | 10.39 | 5.74  (1.28 to 10.39) |
| 77 | Kingdom of Spain | 4.03 | 0.25 | 7.95 | 4.03  (0.25 to 7.95) |
| 78 | Kingdom of Sweden | 3.24 | 0.19 | 6.39 | 3.24  (0.19 to 6.39) |
| 79 | Swiss Confederation | 2.33 | -1.29 | 6.09 | 2.33  (-1.29 to 6.09) |
| 80 | United Kingdom of Great Britain and Northern Ireland | 5.56 | 3.19 | 7.98 | 5.56  (3.19 to 7.98) |
| 81 | Argentine Republic | 6.21 | 1.04 | 11.65 | 6.21  (1.04 to 11.65) |
| 82 | Republic of Chile | 6.05 | 3.9 | 8.24 | 6.05  (3.9 to 8.24) |
| 83 | Eastern Republic of Uruguay | 6.98 | -1.77 | 16.52 | 6.98  (-1.77 to 16.52) |
| 84 | Canada | 13.82 | 9.01 | 18.84 | 13.82  (9.01 to 18.84) |
| 85 | United States of America | 4.07 | 2.36 | 5.81 | 4.07  (2.36 to 5.81) |
| 86 | Antigua and Barbuda | 2.73 | -0.68 | 6.27 | 2.73  (-0.68 to 6.27) |
| 87 | Commonwealth of the Bahamas | 2.6 | -0.72 | 6.03 | 2.6  (-0.72 to 6.03) |
| 88 | Barbados | 3.41 | 1.76 | 5.09 | 3.41  (1.76 to 5.09) |
| 89 | Belize | 3.83 | 2.16 | 5.52 | 3.83  (2.16 to 5.52) |
| 90 | Republic of Cuba | 2.1 | 0.45 | 3.77 | 2.1  (0.45 to 3.77) |
| 91 | Commonwealth of Dominica | 4.06 | 0.64 | 7.58 | 4.06  (0.64 to 7.58) |
| 92 | Dominican Republic | 4.56 | 2.52 | 6.64 | 4.56  (2.52 to 6.64) |
| 93 | Grenada | 4.96 | 2.78 | 7.17 | 4.96  (2.78 to 7.17) |
| 94 | Republic of Guyana | 2.6 | 1.32 | 3.89 | 2.6  (1.32 to 3.89) |
| 95 | Republic of Haiti | 2.42 | 2.35 | 2.5 | 2.42  (2.35 to 2.5) |
| 96 | Jamaica | 4.17 | 2.4 | 5.97 | 4.17  (2.4 to 5.97) |
| 97 | Saint Lucia | 1.94 | -0.7 | 4.64 | 1.94  (-0.7 to 4.64) |
| 98 | Saint Vincent and the Grenadines | 2.02 | -0.87 | 4.98 | 2.02  (-0.87 to 4.98) |
| 99 | Republic of Suriname | 3.78 | 1.23 | 6.39 | 3.78  (1.23 to 6.39) |
| 100 | Republic of Trinidad and Tobago | 2.96 | 1.54 | 4.41 | 2.96  (1.54 to 4.41) |
| 101 | Plurinational State of Bolivia | 3.37 | 3.07 | 3.67 | 3.37  (3.07 to 3.67) |
| 102 | Republic of Ecuador | 2.13 | 1.95 | 2.31 | 2.13  (1.95 to 2.31) |
| 103 | Republic of Peru | 4.17 | 4.02 | 4.31 | 4.17  (4.02 to 4.31) |
| 104 | Republic of Colombia | 2.82 | 0.22 | 5.48 | 2.82  (0.22 to 5.48) |
| 105 | Republic of Costa Rica | 4.1 | -0.08 | 8.45 | 4.1  (-0.08 to 8.45) |
| 106 | Republic of El Salvador | 2.6 | -0.45 | 5.74 | 2.6  (-0.45 to 5.74) |
| 107 | Republic of Guatemala | 1.98 | -1.25 | 5.32 | 1.98  (-1.25 to 5.32) |
| 108 | Republic of Honduras | 5.1 | 2.83 | 7.43 | 5.1  (2.83 to 7.43) |
| 109 | United Mexican States | 4.26 | 1.19 | 7.42 | 4.26  (1.19 to 7.42) |
| 110 | Republic of Nicaragua | 2.68 | -0.42 | 5.88 | 2.68  (-0.42 to 5.88) |
| 111 | Republic of Panama | 2.47 | -1.01 | 6.07 | 2.47  (-1.01 to 6.07) |
| 112 | Bolivarian Republic of Venezuela | 0.61 | -2.62 | 3.96 | 0.61  (-2.62 to 3.96) |
| 113 | Federative Republic of Brazil | 3.7 | 2.4 | 5.02 | 3.7  (2.4 to 5.02) |
| 114 | Republic of Paraguay | 5.31 | 4.56 | 6.06 | 5.31  (4.56 to 6.06) |
| 115 | People's Democratic Republic of Algeria | 4.51 | 1.13 | 8 | 4.51  (1.13 to 8) |
| 116 | Kingdom of Bahrain | 1.42 | -1.94 | 4.89 | 1.42  (-1.94 to 4.89) |
| 117 | Arab Republic of Egypt | 4.13 | -0.07 | 8.5 | 4.13  (-0.07 to 8.5) |
| 118 | Islamic Republic of Iran | 3.02 | -0.28 | 6.43 | 3.02  (-0.28 to 6.43) |
| 119 | Republic of Iraq | 3.86 | -0.18 | 8.07 | 3.86  (-0.18 to 8.07) |
| 120 | Hashemite Kingdom of Jordan | 2.35 | -1.36 | 6.21 | 2.35  (-1.36 to 6.21) |
| 121 | State of Kuwait | -1.46 | -5.04 | 2.26 | -1.46  (-5.04 to 2.26) |
| 122 | Lebanese Republic | 2.82 | -0.12 | 5.86 | 2.82  (-0.12 to 5.86) |
| 123 | State of Libya | 5.27 | 3.43 | 7.14 | 5.27  (3.43 to 7.14) |
| 124 | Kingdom of Morocco | 5.48 | 2.12 | 8.94 | 5.48  (2.12 to 8.94) |
| 125 | Palestine | 2.73 | -0.44 | 5.99 | 2.73  (-0.44 to 5.99) |
| 126 | Sultanate of Oman | 3.52 | -0.84 | 8.07 | 3.52  (-0.84 to 8.07) |
| 127 | State of Qatar | 3.21 | -0.55 | 7.11 | 3.21  (-0.55 to 7.11) |
| 128 | Kingdom of Saudi Arabia | 2.74 | -1.87 | 7.58 | 2.74  (-1.87 to 7.58) |
| 129 | Syrian Arab Republic | 2.8 | -0.57 | 6.29 | 2.8  (-0.57 to 6.29) |
| 130 | Republic of Tunisia | 4.28 | 0.41 | 8.29 | 4.28  (0.41 to 8.29) |
| 131 | Republic of Turkey | 3.66 | 0.55 | 6.86 | 3.66  (0.55 to 6.86) |
| 132 | United Arab Emirates | 3.02 | 0.17 | 5.95 | 3.02  (0.17 to 5.95) |
| 133 | Republic of Yemen | 2.68 | -0.8 | 6.29 | 2.68  (-0.8 to 6.29) |
| 134 | Islamic Republic of Afghanistan | 2.14 | -1.02 | 5.39 | 2.14  (-1.02 to 5.39) |
| 135 | People's Republic of Bangladesh | 3.91 | -0.02 | 7.99 | 3.91  (-0.02 to 7.99) |
| 136 | Kingdom of Bhutan | 3.15 | -0.97 | 7.43 | 3.15  (-0.97 to 7.43) |
| 137 | Republic of India | 3.07 | 0.45 | 5.75 | 3.07  (0.45 to 5.75) |
| 138 | Federal Democratic Republic of Nepal | 4.48 | 1.7 | 7.34 | 4.48  (1.7 to 7.34) |
| 139 | Islamic Republic of Pakistan | 2.2 | -0.3 | 4.77 | 2.2  (-0.3 to 4.77) |
| 140 | Republic of Angola | 1.28 | -2.46 | 5.16 | 1.28  (-2.46 to 5.16) |
| 141 | Central African Republic | 1.39 | -2.03 | 4.93 | 1.39  (-2.03 to 4.93) |
| 142 | Republic of the Congo | 0.92 | -1.04 | 2.93 | 0.92  (-1.04 to 2.93) |
| 143 | Democratic Republic of the Congo | 1.91 | 0.91 | 2.92 | 1.91  (0.91 to 2.92) |
| 144 | Republic of Equatorial Guinea | 4.1 | 2.14 | 6.09 | 4.1  (2.14 to 6.09) |
| 145 | Gabonese Republic | 2.36 | -0.52 | 5.31 | 2.36  (-0.52 to 5.31) |
| 146 | Republic of Burundi | 1.25 | 0.71 | 1.79 | 1.25  (0.71 to 1.79) |
| 147 | Union of the Comoros | 2.51 | 2.11 | 2.92 | 2.51  (2.11 to 2.92) |
| 148 | Republic of Djibouti | 3.69 | 2.83 | 4.56 | 3.69  (2.83 to 4.56) |
| 149 | State of Eritrea | 2.69 | 2.2 | 3.18 | 2.69  (2.2 to 3.18) |
| 150 | Federal Democratic Republic of Ethiopia | 1.79 | 0.6 | 2.99 | 1.79  (0.6 to 2.99) |
| 151 | Republic of Kenya | 3.69 | 1.17 | 6.27 | 3.69  (1.17 to 6.27) |
| 152 | Republic of Madagascar | 3.5 | 2.6 | 4.41 | 3.5  (2.6 to 4.41) |
| 153 | Republic of Malawi | 3.97 | 1.77 | 6.22 | 3.97  (1.77 to 6.22) |
| 154 | Republic of Mauritius | -4.44 | -4.68 | -4.2 | -4.44  (-4.68 to -4.2) |
| 155 | Republic of Mozambique | 3.65 | 1.62 | 5.72 | 3.65  (1.62 to 5.72) |
| 156 | Republic of Rwanda | 1.89 | 1.54 | 2.23 | 1.89  (1.54 to 2.23) |
| 157 | Republic of Seychelles | -0.35 | -3.52 | 2.92 | -0.35  (-3.52 to 2.92) |
| 158 | Federal Republic of Somalia | 2.61 | 0.8 | 4.45 | 2.61  (0.8 to 4.45) |
| 159 | United Republic of Tanzania | 1.97 | 1.66 | 2.29 | 1.97  (1.66 to 2.29) |
| 160 | Republic of Uganda | 1.52 | 0.77 | 2.28 | 1.52  (0.77 to 2.28) |
| 161 | Republic of Zambia | 0.76 | -0.67 | 2.22 | 0.76  (-0.67 to 2.22) |
| 162 | Republic of Botswana | 3.02 | 1.64 | 4.41 | 3.02  (1.64 to 4.41) |
| 163 | Kingdom of Lesotho | 5.68 | 3.32 | 8.09 | 5.68  (3.32 to 8.09) |
| 164 | Republic of Namibia | 3.89 | 2.96 | 4.82 | 3.89  (2.96 to 4.82) |
| 165 | Republic of South Africa | 4.43 | 2.82 | 6.06 | 4.43  (2.82 to 6.06) |
| 166 | Kingdom of Eswatini | 4.68 | 1.65 | 7.8 | 4.68  (1.65 to 7.8) |
| 167 | Republic of Zimbabwe | 3.56 | 2.83 | 4.31 | 3.56  (2.83 to 4.31) |
| 168 | Republic of Benin | 1.18 | 0.39 | 1.98 | 1.18  (0.39 to 1.98) |
| 169 | Burkina Faso | 1.57 | 0.05 | 3.11 | 1.57  (0.05 to 3.11) |
| 170 | Republic of Cameroon | 0.92 | 0.43 | 1.4 | 0.92  (0.43 to 1.4) |
| 171 | Republic of Cabo Verde | 3.66 | 2.93 | 4.39 | 3.66  (2.93 to 4.39) |
| 172 | Republic of Chad | 2.39 | 1.62 | 3.17 | 2.39  (1.62 to 3.17) |
| 173 | Republic of C么te d'Ivoire | 1.08 | 0.04 | 2.13 | 1.08  (0.04 to 2.13) |
| 174 | Republic of the Gambia | 3.54 | 2.53 | 4.56 | 3.54  (2.53 to 4.56) |
| 175 | Republic of Ghana | 1.5 | 0.41 | 2.59 | 1.5  (0.41 to 2.59) |
| 176 | Republic of Guinea | 2.16 | 1.85 | 2.46 | 2.16  (1.85 to 2.46) |
| 177 | Republic of Guinea-Bissau | 1.32 | -0.71 | 3.39 | 1.32  (-0.71 to 3.39) |
| 178 | Republic of Liberia | 1.69 | 0.7 | 2.69 | 1.69  (0.7 to 2.69) |
| 179 | Republic of Mali | 2.62 | 0.87 | 4.4 | 2.62  (0.87 to 4.4) |
| 180 | Islamic Republic of Mauritania | 0.9 | -0.57 | 2.4 | 0.9  (-0.57 to 2.4) |
| 181 | Republic of the Niger | 0.78 | -0.15 | 1.71 | 0.78  (-0.15 to 1.71) |
| 182 | Federal Republic of Nigeria | 1.73 | -0.63 | 4.15 | 1.73  (-0.63 to 4.15) |
| 183 | Democratic Republic of Sao Tome and Principe | 2.27 | 1.44 | 3.1 | 2.27  (1.44 to 3.1) |
| 184 | Republic of Senegal | 1.89 | 1.12 | 2.67 | 1.89  (1.12 to 2.67) |
| 185 | Republic of Sierra Leone | 0.92 | -0.27 | 2.12 | 0.92  (-0.27 to 2.12) |
| 186 | Togolese Republic | 2.3 | 1.83 | 2.77 | 2.3  (1.83 to 2.77) |
| 187 | American Samoa | 2.95 | 1.94 | 3.97 | 2.95  (1.94 to 3.97) |
| 188 | Bermuda | 1.18 | -1.58 | 4.02 | 1.18  (-1.58 to 4.02) |
| 189 | Cook Islands | 1.51 | 0.09 | 2.96 | 1.51  (0.09 to 2.96) |
| 190 | Guam | 2.89 | 0.48 | 5.35 | 2.89  (0.48 to 5.35) |
| 191 | Greenland | 7.45 | 0.27 | 15.15 | 7.45  (0.27 to 15.15) |
| 192 | Principality of Monaco | 5.26 | 1.2 | 9.48 | 5.26  (1.2 to 9.48) |
| 193 | Republic of Nauru | 1.03 | -0.51 | 2.58 | 1.03  (-0.51 to 2.58) |
| 194 | Republic of Niue | 2.29 | 0.25 | 4.37 | 2.29  (0.25 to 4.37) |
| 195 | Northern Mariana Islands | 2.5 | 1.17 | 3.85 | 2.5  (1.17 to 3.85) |
| 196 | Republic of Palau | 1.15 | -16.66 | 22.76 | 1.15  (-16.66 to 22.76) |
| 197 | Puerto Rico | 3.24 | 0.55 | 6.01 | 3.24  (0.55 to 6.01) |
| 198 | Saint Kitts and Nevis | 2.83 | -0.06 | 5.81 | 2.83  (-0.06 to 5.81) |
| 199 | Republic of San Marino | 3.61 | -0.6 | 8 | 3.61  (-0.6 to 8) |
| 200 | Tokelau | 2.53 | 1.69 | 3.37 | 2.53  (1.69 to 3.37) |
| 201 | Tuvalu | 1.75 | 0.46 | 3.06 | 1.75  (0.46 to 3.06) |
| 202 | United States Virgin Islands | 3.94 | 0.76 | 7.21 | 3.94  (0.76 to 7.21) |
| 203 | Republic of South Sudan | 3.76 | 2.68 | 4.86 | 3.76  (2.68 to 4.86) |
| 204 | Republic of Sudan | 3.85 | 0.42 | 7.4 | 3.85  (0.42 to 7.4) |

|  | location | EAPC | LCI | UCI | EAPC_CI |
| --- | --- | --- | --- | --- | --- |
| 1 | Republic of Indonesia | 1.27 | -0.78 | 3.36 | 1.27  (-0.78 to 3.36) |
| 2 | People's Republic of China | 0.78 | -0.69 | 2.27 | 0.78  (-0.69 to 2.27) |
| 3 | Democratic People's Republic of Korea | 0.84 | -1.41 | 3.14 | 0.84  (-1.41 to 3.14) |
| 4 | Taiwan (Province of China) | 3.15 | -0.4 | 6.83 | 3.15  (-0.4 to 6.83) |
| 5 | Kingdom of Cambodia | 0.77 | -0.55 | 2.1 | 0.77  (-0.55 to 2.1) |
| 6 | Lao People's Democratic Republic | 0.38 | -2.27 | 3.1 | 0.38  (-2.27 to 3.1) |
| 7 | Democratic Socialist Republic of Sri Lanka | 1.07 | -1.53 | 3.73 | 1.07  (-1.53 to 3.73) |
| 8 | Malaysia | 3.31 | 1.02 | 5.65 | 3.31  (1.02 to 5.65) |
| 9 | Republic of Maldives | -0.72 | -4.42 | 3.12 | -0.72  (-4.42 to 3.12) |
| 10 | Kingdom of Thailand | -0.23 | -3.16 | 2.79 | -0.23  (-3.16 to 2.79) |
| 11 | Republic of the Union of Myanmar | 1.57 | -0.03 | 3.18 | 1.57  (-0.03 to 3.18) |
| 12 | Republic of the Philippines | 2.08 | -1.06 | 5.33 | 2.08  (-1.06 to 5.33) |
| 13 | Democratic Republic of Timor-Leste | 1.52 | 0.79 | 2.26 | 1.52  (0.79 to 2.26) |
| 14 | Socialist Republic of Viet Nam | 1.09 | -3.81 | 6.25 | 1.09  (-3.81 to 6.25) |
| 15 | Republic of Fiji | 1.78 | 0.47 | 3.11 | 1.78  (0.47 to 3.11) |
| 16 | Republic of Kiribati | 1.23 | 0.34 | 2.14 | 1.23  (0.34 to 2.14) |
| 17 | Republic of the Marshall Islands | 2.02 | 1.41 | 2.65 | 2.02  (1.41 to 2.65) |
| 18 | Federated States of Micronesia | 1.61 | 0.4 | 2.84 | 1.61  (0.4 to 2.84) |
| 19 | Independent State of Samoa | 1.3 | -1.32 | 3.99 | 1.3  (-1.32 to 3.99) |
| 20 | Solomon Islands | 0.44 | -1.74 | 2.67 | 0.44  (-1.74 to 2.67) |
| 21 | Independent State of Papua New Guinea | 0.01 | -1.19 | 1.22 | 0.01  (-1.19 to 1.22) |
| 22 | Kingdom of Tonga | 1.37 | -1.32 | 4.12 | 1.37  (-1.32 to 4.12) |
| 23 | Republic of Vanuatu | 1.41 | -0.19 | 3.04 | 1.41  (-0.19 to 3.04) |
| 24 | Republic of Armenia | 2.01 | -0.59 | 4.67 | 2.01  (-0.59 to 4.67) |
| 25 | Republic of Azerbaijan | 2.46 | 0.38 | 4.59 | 2.46  (0.38 to 4.59) |
| 26 | Georgia | 0.96 | -1.87 | 3.88 | 0.96  (-1.87 to 3.88) |
| 27 | Mongolia | 2.34 | 0.07 | 4.67 | 2.34  (0.07 to 4.67) |
| 28 | Republic of Kazakhstan | -1.47 | -3.62 | 0.73 | -1.47  (-3.62 to 0.73) |
| 29 | Kyrgyz Republic | -0.41 | -2.35 | 1.57 | -0.41  (-2.35 to 1.57) |
| 30 | Republic of Tajikistan | 1.33 | 0.29 | 2.37 | 1.33  (0.29 to 2.37) |
| 31 | Turkmenistan | 2.02 | -1.17 | 5.31 | 2.02  (-1.17 to 5.31) |
| 32 | Republic of Uzbekistan | 2.49 | 1.16 | 3.84 | 2.49  (1.16 to 3.84) |
| 33 | Republic of Albania | 1.03 | -1.27 | 3.37 | 1.03  (-1.27 to 3.37) |
| 34 | Bosnia and Herzegovina | 1.89 | -0.79 | 4.63 | 1.89  (-0.79 to 4.63) |
| 35 | Republic of Bulgaria | -0.55 | -1.81 | 0.72 | -0.55  (-1.81 to 0.72) |
| 36 | Republic of Croatia | 2.91 | 1.15 | 4.7 | 2.91  (1.15 to 4.7) |
| 37 | Czech Republic | 0.78 | -0.98 | 2.58 | 0.78  (-0.98 to 2.58) |
| 38 | North Macedonia | 1.39 | -0.07 | 2.87 | 1.39  (-0.07 to 2.87) |
| 39 | Hungary | 2.15 | 0.03 | 4.31 | 2.15  (0.03 to 4.31) |
| 40 | Montenegro | 2.69 | 1.02 | 4.38 | 2.69  (1.02 to 4.38) |
| 41 | Republic of Poland | 6.07 | 4.94 | 7.22 | 6.07  (4.94 to 7.22) |
| 42 | Romania | 5.8 | 3.4 | 8.26 | 5.8  (3.4 to 8.26) |
| 43 | Slovak Republic | 1.28 | -0.43 | 3.02 | 1.28  (-0.43 to 3.02) |
| 44 | Republic of Serbia | 0.99 | 0.03 | 1.96 | 0.99  (0.03 to 1.96) |
| 45 | Republic of Slovenia | 2.32 | 0.22 | 4.47 | 2.32  (0.22 to 4.47) |
| 46 | Republic of Belarus | 0.75 | -1.59 | 3.15 | 0.75  (-1.59 to 3.15) |
| 47 | Republic of Estonia | 1.31 | -0.38 | 3.03 | 1.31  (-0.38 to 3.03) |
| 48 | Republic of Latvia | 2.67 | 1.64 | 3.71 | 2.67  (1.64 to 3.71) |
| 49 | Republic of Lithuania | 3.65 | 0.59 | 6.8 | 3.65  (0.59 to 6.8) |
| 50 | Republic of Moldova | 1.12 | -0.96 | 3.25 | 1.12  (-0.96 to 3.25) |
| 51 | Russian Federation | 2.64 | 0.45 | 4.88 | 2.64  (0.45 to 4.88) |
| 52 | Ukraine | 0.02 | -2.87 | 3 | 0.02  (-2.87 to 3) |
| 53 | Brunei Darussalam | 2.05 | -3.48 | 7.89 | 2.05  (-3.48 to 7.89) |
| 54 | Japan | 1.57 | -6.02 | 9.77 | 1.57  (-6.02 to 9.77) |
| 55 | Republic of Korea | 0.78 | -3.92 | 5.71 | 0.78  (-3.92 to 5.71) |
| 56 | Australia | 6.48 | 3.41 | 9.65 | 6.48  (3.41 to 9.65) |
| 57 | Republic of Singapore | 1.97 | -4.15 | 8.49 | 1.97  (-4.15 to 8.49) |
| 58 | New Zealand | 4.04 | 0.65 | 7.53 | 4.04  (0.65 to 7.53) |
| 59 | Republic of Austria | 5.97 | 1.81 | 10.31 | 5.97  (1.81 to 10.31) |
| 60 | Principality of Andorra | 2.37 | 0.82 | 3.93 | 2.37  (0.82 to 3.93) |
| 61 | Kingdom of Belgium | 4.32 | 0.87 | 7.89 | 4.32  (0.87 to 7.89) |
| 62 | Republic of Cyprus | 3.39 | -0.56 | 7.5 | 3.39  (-0.56 to 7.5) |
| 63 | Kingdom of Denmark | 6.18 | 2.25 | 10.27 | 6.18  (2.25 to 10.27) |
| 64 | Republic of Finland | 4.7 | 1.33 | 8.18 | 4.7  (1.33 to 8.18) |
| 65 | Federal Republic of Germany | 4.7 | 1.57 | 7.93 | 4.7  (1.57 to 7.93) |
| 66 | French Republic | 3.37 | -2.35 | 9.42 | 3.37  (-2.35 to 9.42) |
| 67 | Hellenic Republic | 6.09 | 3.57 | 8.67 | 6.09  (3.57 to 8.67) |
| 68 | Republic of Iceland | 3.64 | -0.09 | 7.5 | 3.64  (-0.09 to 7.5) |
| 69 | Ireland | 4.57 | 1.78 | 7.44 | 4.57  (1.78 to 7.44) |
| 70 | Republic of Italy | 1 | -4.42 | 6.73 | 1  (-4.42 to 6.73) |
| 71 | State of Israel | 2.47 | -0.75 | 5.79 | 2.47  (-0.75 to 5.79) |
| 72 | Grand Duchy of Luxembourg | 2.79 | 0.67 | 4.95 | 2.79  (0.67 to 4.95) |
| 73 | Republic of Malta | 3.38 | -0.75 | 7.68 | 3.38  (-0.75 to 7.68) |
| 74 | Kingdom of the Netherlands | 5.68 | 2.42 | 9.05 | 5.68  (2.42 to 9.05) |
| 75 | Portuguese Republic | 5.22 | 0.54 | 10.12 | 5.22  (0.54 to 10.12) |
| 76 | Kingdom of Norway | 4.2 | 2.67 | 5.74 | 4.2  (2.67 to 5.74) |
| 77 | Kingdom of Spain | 3.17 | -0.71 | 7.2 | 3.17  (-0.71 to 7.2) |
| 78 | Kingdom of Sweden | 2.9 | -0.28 | 6.18 | 2.9  (-0.28 to 6.18) |
| 79 | Swiss Confederation | 1.65 | -1.89 | 5.31 | 1.65  (-1.89 to 5.31) |
| 80 | United Kingdom of Great Britain and Northern Ireland | 4.84 | 2.58 | 7.15 | 4.84  (2.58 to 7.15) |
| 81 | Argentine Republic | 5.9 | 0.71 | 11.37 | 5.9  (0.71 to 11.37) |
| 82 | Republic of Chile | 5.65 | 3.5 | 7.83 | 5.65  (3.5 to 7.83) |
| 83 | Eastern Republic of Uruguay | 6.74 | -2.21 | 16.52 | 6.74  (-2.21 to 16.52) |
| 84 | United States of America | 3.75 | 1.89 | 5.64 | 3.75  (1.89 to 5.64) |
| 85 | Canada | 14.27 | 9.35 | 19.42 | 14.27  (9.35 to 19.42) |
| 86 | Antigua and Barbuda | 2.62 | -0.87 | 6.23 | 2.62  (-0.87 to 6.23) |
| 87 | Barbados | 3.21 | 1.46 | 5 | 3.21  (1.46 to 5) |
| 88 | Commonwealth of the Bahamas | 2.39 | -1.04 | 5.92 | 2.39  (-1.04 to 5.92) |
| 89 | Belize | 3.75 | 2.01 | 5.52 | 3.75  (2.01 to 5.52) |
| 90 | Republic of Cuba | 1.89 | 0.23 | 3.58 | 1.89  (0.23 to 3.58) |
| 91 | Commonwealth of Dominica | 3.84 | 0.34 | 7.45 | 3.84  (0.34 to 7.45) |
| 92 | Dominican Republic | 4.24 | 2.13 | 6.4 | 4.24  (2.13 to 6.4) |
| 93 | Republic of Guyana | 2.47 | 1.11 | 3.86 | 2.47  (1.11 to 3.86) |
| 94 | Grenada | 4.8 | 2.54 | 7.11 | 4.8  (2.54 to 7.11) |
| 95 | Republic of Haiti | 2.19 | 2.08 | 2.3 | 2.19  (2.08 to 2.3) |
| 96 | Jamaica | 3.97 | 2.13 | 5.84 | 3.97  (2.13 to 5.84) |
| 97 | Saint Lucia | 1.77 | -0.95 | 4.56 | 1.77  (-0.95 to 4.56) |
| 98 | Republic of Suriname | 3.55 | 0.9 | 6.28 | 3.55  (0.9 to 6.28) |
| 99 | Republic of Trinidad and Tobago | 2.82 | 1.35 | 4.32 | 2.82  (1.35 to 4.32) |
| 100 | Saint Vincent and the Grenadines | 1.9 | -1.02 | 4.9 | 1.9  (-1.02 to 4.9) |
| 101 | Plurinational State of Bolivia | 2.98 | 2.72 | 3.24 | 2.98  (2.72 to 3.24) |
| 102 | Republic of Peru | 3.66 | 3.53 | 3.78 | 3.66  (3.53 to 3.78) |
| 103 | Republic of Ecuador | 1.63 | 1.48 | 1.79 | 1.63  (1.48 to 1.79) |
| 104 | Republic of Colombia | 2.16 | -0.39 | 4.77 | 2.16  (-0.39 to 4.77) |
| 105 | Republic of Costa Rica | 3.71 | -0.48 | 8.07 | 3.71  (-0.48 to 8.07) |
| 106 | Republic of El Salvador | 2.15 | -0.92 | 5.31 | 2.15  (-0.92 to 5.31) |
| 107 | Republic of Honduras | 4.71 | 2.44 | 7.03 | 4.71  (2.44 to 7.03) |
| 108 | Republic of Guatemala | 1.51 | -1.69 | 4.81 | 1.51  (-1.69 to 4.81) |
| 109 | United Mexican States | 3.95 | 0.86 | 7.13 | 3.95  (0.86 to 7.13) |
| 110 | Republic of Nicaragua | 2.32 | -0.79 | 5.52 | 2.32  (-0.79 to 5.52) |
| 111 | Republic of Panama | 1.98 | -1.53 | 5.61 | 1.98  (-1.53 to 5.61) |
| 112 | Bolivarian Republic of Venezuela | 0.18 | -3.06 | 3.53 | 0.18  (-3.06 to 3.53) |
| 113 | Federative Republic of Brazil | 3.31 | 1.92 | 4.73 | 3.31  (1.92 to 4.73) |
| 114 | People's Democratic Republic of Algeria | 4.3 | 0.84 | 7.87 | 4.3  (0.84 to 7.87) |
| 115 | Republic of Paraguay | 5.01 | 4.21 | 5.82 | 5.01  (4.21 to 5.82) |
| 116 | Kingdom of Bahrain | 1.39 | -2.05 | 4.95 | 1.39  (-2.05 to 4.95) |
| 117 | Arab Republic of Egypt | 4.12 | -0.23 | 8.66 | 4.12  (-0.23 to 8.66) |
| 118 | State of Kuwait | -1.87 | -5.64 | 2.04 | -1.87  (-5.64 to 2.04) |
| 119 | Islamic Republic of Iran | 2.31 | -0.92 | 5.66 | 2.31  (-0.92 to 5.66) |
| 120 | Lebanese Republic | 2.51 | -0.63 | 5.75 | 2.51  (-0.63 to 5.75) |
| 121 | Republic of Iraq | 3.72 | -0.43 | 8.04 | 3.72  (-0.43 to 8.04) |
| 122 | Hashemite Kingdom of Jordan | 2.17 | -1.68 | 6.16 | 2.17  (-1.68 to 6.16) |
| 123 | State of Libya | 5.17 | 3.28 | 7.09 | 5.17  (3.28 to 7.09) |
| 124 | Sultanate of Oman | 3.44 | -1.16 | 8.24 | 3.44  (-1.16 to 8.24) |
| 125 | Kingdom of Morocco | 5.48 | 2.05 | 9.03 | 5.48  (2.05 to 9.03) |
| 126 | State of Qatar | 3.02 | -0.93 | 7.13 | 3.02  (-0.93 to 7.13) |
| 127 | Palestine | 2.72 | -0.59 | 6.15 | 2.72  (-0.59 to 6.15) |
| 128 | Kingdom of Saudi Arabia | 2.74 | -1.92 | 7.63 | 2.74  (-1.92 to 7.63) |
| 129 | Syrian Arab Republic | 2.67 | -0.81 | 6.27 | 2.67  (-0.81 to 6.27) |
| 130 | Republic of Tunisia | 4.08 | 0.08 | 8.25 | 4.08  (0.08 to 8.25) |
| 131 | Republic of Turkey | 3.26 | -0.03 | 6.66 | 3.26  (-0.03 to 6.66) |
| 132 | People's Republic of Bangladesh | 3.78 | -0.22 | 7.95 | 3.78  (-0.22 to 7.95) |
| 133 | United Arab Emirates | 3.13 | 0.09 | 6.27 | 3.13  (0.09 to 6.27) |
| 134 | Kingdom of Bhutan | 2.75 | -1.44 | 7.12 | 2.75  (-1.44 to 7.12) |
| 135 | Republic of Yemen | 2.49 | -1.14 | 6.25 | 2.49  (-1.14 to 6.25) |
| 136 | Islamic Republic of Afghanistan | 1.83 | -1.37 | 5.13 | 1.83  (-1.37 to 5.13) |
| 137 | Republic of India | 2.68 | 0.08 | 5.34 | 2.68  (0.08 to 5.34) |
| 138 | Republic of Angola | 1.18 | -2.67 | 5.18 | 1.18  (-2.67 to 5.18) |
| 139 | Federal Democratic Republic of Nepal | 4.17 | 1.35 | 7.07 | 4.17  (1.35 to 7.07) |
| 140 | Democratic Republic of the Congo | 1.83 | 0.78 | 2.89 | 1.83  (0.78 to 2.89) |
| 141 | Islamic Republic of Pakistan | 2.07 | -0.56 | 4.78 | 2.07  (-0.56 to 4.78) |
| 142 | Republic of Equatorial Guinea | 3.92 | 1.89 | 5.99 | 3.92  (1.89 to 5.99) |
| 143 | Central African Republic | 1.38 | -2.19 | 5.08 | 1.38  (-2.19 to 5.08) |
| 144 | Republic of the Congo | 0.88 | -1.19 | 2.98 | 0.88  (-1.19 to 2.98) |
| 145 | Gabonese Republic | 2.32 | -0.76 | 5.5 | 2.32  (-0.76 to 5.5) |
| 146 | Republic of Djibouti | 3.53 | 2.54 | 4.54 | 3.53  (2.54 to 4.54) |
| 147 | State of Eritrea | 2.5 | 1.87 | 3.13 | 2.5  (1.87 to 3.13) |
| 148 | Federal Democratic Republic of Ethiopia | 1.5 | 0.42 | 2.59 | 1.5  (0.42 to 2.59) |
| 149 | Republic of Burundi | 1.25 | 0.83 | 1.68 | 1.25  (0.83 to 1.68) |
| 150 | Republic of Kenya | 3.6 | 0.9 | 6.37 | 3.6  (0.9 to 6.37) |
| 151 | Union of the Comoros | 2.36 | 1.83 | 2.89 | 2.36  (1.83 to 2.89) |
| 152 | Republic of Madagascar | 3.58 | 2.62 | 4.55 | 3.58  (2.62 to 4.55) |
| 153 | Republic of Malawi | 3.85 | 1.46 | 6.29 | 3.85  (1.46 to 6.29) |
| 154 | Republic of Mauritius | -4.66 | -5.08 | -4.24 | -4.66  (-5.08 to -4.24) |
| 155 | Republic of Mozambique | 3.53 | 1.24 | 5.86 | 3.53  (1.24 to 5.86) |
| 156 | Republic of Rwanda | 1.76 | 1.28 | 2.23 | 1.76  (1.28 to 2.23) |
| 157 | Federal Republic of Somalia | 2.42 | 0.48 | 4.39 | 2.42  (0.48 to 4.39) |
| 158 | Republic of Seychelles | -0.52 | -3.96 | 3.03 | -0.52  (-3.96 to 3.03) |
| 159 | United Republic of Tanzania | 1.8 | 1.53 | 2.07 | 1.8  (1.53 to 2.07) |
| 160 | Republic of Uganda | 1.47 | 0.49 | 2.46 | 1.47  (0.49 to 2.46) |
| 161 | Republic of Zambia | 0.51 | -1.01 | 2.06 | 0.51  (-1.01 to 2.06) |
| 162 | Kingdom of Lesotho | 5.71 | 3.17 | 8.32 | 5.71  (3.17 to 8.32) |
| 163 | Republic of Botswana | 2.91 | 1.42 | 4.43 | 2.91  (1.42 to 4.43) |
| 164 | Republic of Namibia | 3.8 | 2.78 | 4.83 | 3.8  (2.78 to 4.83) |
| 165 | Republic of South Africa | 4.33 | 2.6 | 6.09 | 4.33  (2.6 to 6.09) |
| 166 | Kingdom of Eswatini | 4.59 | 1.37 | 7.91 | 4.59  (1.37 to 7.91) |
| 167 | Republic of Benin | 1.06 | 0.18 | 1.95 | 1.06  (0.18 to 1.95) |
| 168 | Republic of Zimbabwe | 3.48 | 2.7 | 4.26 | 3.48  (2.7 to 4.26) |
| 169 | Burkina Faso | 1.38 | -0.31 | 3.11 | 1.38  (-0.31 to 3.11) |
| 170 | Republic of Cameroon | 0.74 | 0.08 | 1.41 | 0.74  (0.08 to 1.41) |
| 171 | Republic of Chad | 2.34 | 1.52 | 3.17 | 2.34  (1.52 to 3.17) |
| 172 | Republic of Cabo Verde | 3.63 | 2.86 | 4.4 | 3.63  (2.86 to 4.4) |
| 173 | Republic of C么te d'Ivoire | 0.89 | -0.29 | 2.09 | 0.89  (-0.29 to 2.09) |
| 174 | Republic of the Gambia | 3.36 | 2.12 | 4.61 | 3.36  (2.12 to 4.61) |
| 175 | Republic of Ghana | 1.27 | 0.11 | 2.44 | 1.27  (0.11 to 2.44) |
| 176 | Republic of Guinea | 2.05 | 1.61 | 2.49 | 2.05  (1.61 to 2.49) |
| 177 | Republic of Liberia | 1.62 | 0.54 | 2.72 | 1.62  (0.54 to 2.72) |
| 178 | Republic of Guinea-Bissau | 1.21 | -0.99 | 3.45 | 1.21  (-0.99 to 3.45) |
| 179 | Republic of Mali | 2.48 | 0.65 | 4.34 | 2.48  (0.65 to 4.34) |
| 180 | Islamic Republic of Mauritania | 0.65 | -0.9 | 2.23 | 0.65  (-0.9 to 2.23) |
| 181 | Republic of the Niger | 0.58 | -0.44 | 1.61 | 0.58  (-0.44 to 1.61) |
| 182 | Democratic Republic of Sao Tome and Principe | 2.23 | 1.38 | 3.1 | 2.23  (1.38 to 3.1) |
| 183 | Federal Republic of Nigeria | 1.6 | -0.59 | 3.84 | 1.6  (-0.59 to 3.84) |
| 184 | Republic of Senegal | 1.68 | 0.8 | 2.57 | 1.68  (0.8 to 2.57) |
| 185 | Republic of Sierra Leone | 0.83 | -0.44 | 2.11 | 0.83  (-0.44 to 2.11) |
| 186 | Togolese Republic | 2.19 | 1.9 | 2.48 | 2.19  (1.9 to 2.48) |
| 187 | American Samoa | 2.59 | 1.6 | 3.6 | 2.59  (1.6 to 3.6) |
| 188 | Cook Islands | 1.02 | -0.43 | 2.49 | 1.02  (-0.43 to 2.49) |
| 189 | Bermuda | 0.64 | -2.3 | 3.67 | 0.64  (-2.3 to 3.67) |
| 190 | Greenland | 7.39 | -0.14 | 15.48 | 7.39  (-0.14 to 15.48) |
| 191 | Guam | 2.47 | 0.03 | 4.98 | 2.47  (0.03 to 4.98) |
| 192 | Principality of Monaco | 4.79 | 0.62 | 9.13 | 4.79  (0.62 to 9.13) |
| 193 | Republic of Nauru | 0.98 | -0.54 | 2.53 | 0.98  (-0.54 to 2.53) |
| 194 | Republic of Niue | 2.06 | -0.13 | 4.3 | 2.06  (-0.13 to 4.3) |
| 195 | Northern Mariana Islands | 2.29 | 0.93 | 3.66 | 2.29  (0.93 to 3.66) |
| 196 | Republic of Palau | 1.1 | -16.81 | 22.87 | 1.1  (-16.81 to 22.87) |
| 197 | Saint Kitts and Nevis | 2.97 | -0.02 | 6.05 | 2.97  (-0.02 to 6.05) |
| 198 | Republic of San Marino | 3.01 | -1.24 | 7.44 | 3.01  (-1.24 to 7.44) |
| 199 | Puerto Rico | 2.74 | -0.03 | 5.59 | 2.74  (-0.03 to 5.59) |
| 200 | Tokelau | 2.33 | 1.65 | 3.01 | 2.33  (1.65 to 3.01) |
| 201 | Tuvalu | 1.42 | 0.15 | 2.7 | 1.42  (0.15 to 2.7) |
| 202 | United States Virgin Islands | 3.45 | 0.12 | 6.89 | 3.45  (0.12 to 6.89) |
| 203 | Republic of South Sudan | 3.8 | 2.58 | 5.03 | 3.8  (2.58 to 5.03) |
| 204 | Republic of Sudan | 3.73 | 0.24 | 7.33 | 3.73  (0.24 to 7.33) |
